# EMMAs: Implementation and Assessment of a Suite of Cross-Disciplinary, Case-Based High School Activities to Explore Three-Dimensional Molecular Structure, Noncovalent Interactions, and Molecular Dynamics

**DOI:** 10.1021/acs.jchemed.4c00036

**Published:** 2024-05-10

**Authors:** Parthena E. Kotsalidis, Shelby N. Kranc, Martin Berryman, Mala L. Radhakrishnan, Donald E. Elmore

**Affiliations:** † Biochemistry Program, Wellesley College, Wellesley, Massachusetts 02481, United States; ‡ Chemistry Department, Wellesley College, Wellesley, Massachusetts 02481, United States; § Lincoln-Sudbury Regional High School, Sudbury, Massachusetts 01776, United States

**Keywords:** High School/Introductory Chemistry, Curriculum, Interdisciplinary/Multidisciplinary, Computer-Based Learning, Molecular Dynamics, Molecular Modeling, Noncovalent Interactions

## Abstract

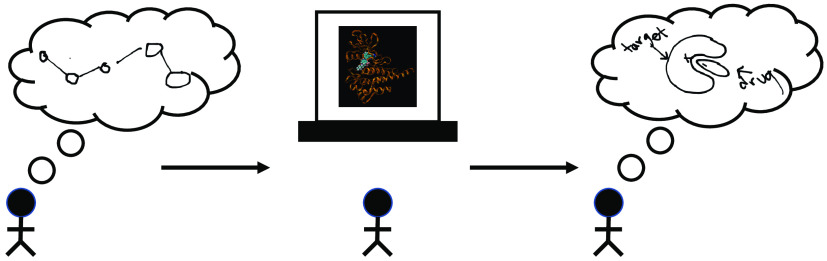

Students frequently develop misconceptions about noncovalent interactions that make it challenging for them to appropriately interpret aspects of molecular structure and interactions critical to myriad applications. We hypothesized that computational molecular modeling and visualization could provide a valuable approach to help address these core misconceptions when students are first exposed to these concepts in secondary school chemistry courses. Here, we present a series of activities exploring biomolecular drug–target interactions using molecular visualization software and an introduction to molecular dynamics methods that were implemented in secondary school classrooms. A pre- and postsurvey approach that incorporated Likert response type, written free response, and drawing-based items demonstrated that students gained an enhanced conceptualization of intermolecular interactions, particularly related to aspects of shape complementarity, after completing the activities. Students also expressed increased comfort with and facility in utilizing different three-dimensional representations of molecules in their postsurvey responses. The activities led to an increased appreciation of interdisciplinary connections of chemistry with mathematics and physics. Overall, the modular activities presented provide a relatively time-efficient and accessible manner to help promote an understanding of a traditionally challenging topic for beginning chemistry students while introducing them to contemporary research tools.

Noncovalent forces, which are responsible for intermolecular interactions, are crucial to understanding and predicting key physical processes across disciplines, from biology to materials science to engineering.^1−4^ However, the study of such interactions is often challenging, with students at the undergraduate level often confusing them with covalent bonds within a molecule^5^ or confusing the concept of “interaction” with “reaction”.^6^ Moreover, students can often demonstrate an inadequate applied understanding of noncovalent interactions—for example, hydrogen bonds—when conducting practical analyses, such as predicting relative boiling points.^7^ Such confusions are likely not helped by (1) the fact that the noncovalent forces responsible for *intermolecular* interactions can *also* occur *within* a larger molecule, such as a protein, and (2) terminology such as “hydrogen bond” suggests a parity with other, covalent “bonds”.^5^ Given the deep-seated confusion regarding noncovalent interactions and how they differ in strength, character, and function from covalent bonds, it is important to engage students when they initially learn this content in effective activities that can help clarify these distinctions.

Understanding noncovalent interactions is particularly challenging because their command requires student facility with conceptualizing the 3D shapes of molecules and predicting their consequent physical properties, thus necessitating a student to connect causal concepts from multiple points in a curriculum in scaffolded ways.^8,9^ Predicting noncovalent interactions often requires translating from commonly used static, 2D representations such as Lewis structures to the potentially dynamic, 3D “realities” that determine them. Also, there are many ways to represent the same molecular system, with each representation having its own strengths and weaknesses in terms of the information it can convey. Concrete and/or tactile models and corresponding activities can benefit students’ abilities to move between and interpret both 2D and 3D representations,^10^ and such models could also engage students and increase understanding.^11^ Easily transferable and engaging activities could greatly benefit students in understanding noncovalent interactions during their first formal introduction to them at the high school level. An early emphasis of these topics may be especially important because it appears that student representations of intermolecular forces do not seem to change after their experiences in general chemistry.^8^

Computer-based visualization tools can effectively and efficiently serve this purpose, and they can increase the ability of high school students to develop an understanding of chemical representations.^12^ For example, previous research has emphasized the value of integrating animations, such as those available in VisChem and Connected Chemistry, to help secondary school students understand molecular processes.^13−15^ Other examples have shown the value of using visualization approaches to help secondary students integrate knowledge when considering topics in chemical reactivity.^16,17^ While they are clearly valuable for student learning, animations can also involve simplifications, meaning that there can be additional value if students engage with other visual molecular models or simulations.^13,14^

Software packages like PyMOL (Schrodinger, LLC), UCSF-Chimera,^18^ Odyssey (Wave function, Inc., Irvine, CA), Avogadro,^19^ IQMol (iqmol.org), and Visual Molecular Dynamics (VMD)^20^ can allow students to manipulate and move between multiple 3D molecular representations. Many of these tools could be used as a starting point to incorporate computer visualizing into a classroom setting,^21^ and there are several existing resources and activities using computational or virtual reality models to facilitate student understanding of 3D molecular structure, dynamics, and/or interactions at the undergraduate level.^21−31^ There are also some noteworthy examples at the high school level, many of which focus on students considering aspects of protein secondary and tertiary structure.^32−34^ Our activities add uniquely to this literature by focusing more specifically on atomic-level aspects of noncovalent interactions and three-dimensional molecular structure that are a central aspect of many high school chemistry courses. Many previous activities for high school students effectively used adapted/simplified versions of modern computational technologies^33^ or web-based viewers.^35^ Although we recognize the potential technical hurdles for high school students using potentially complex software primarily designed for researchers, such as VMD,^36^ here we aimed to create activities that would give students an on-ramp to using these packages. Similar to Burgin et al.,^32^ we felt that exposing students to the “full” versions of computational technologies used by career scientists would help emphasize the real-world utility of these tools beyond the classroom. Moreover, the ability for students to play a more active role in creating representations may help them better appreciate that molecular representations are not an end goal for chemists but are merely imperfect tools to make sense of and understand how underlying chemistry concepts apply to particular systems.^37^

Finally, the importance of noncovalent interactions and 3D molecular structures can become especially apparent to students in an interdisciplinary context. Indeed, many of the computational activities noted above used biological examples to reinforce or highlight concepts related to chemistry. Making cross-disciplinary connections within STEM can enable students to move beyond disciplinary “silos” and to understand multifaceted approaches needed to tackle real-world problems.^38,39^ Although the assessment of interdisciplinary STEM engagement can be challenging, such experiences can both help students learn disciplinary content and increase their positive attitude toward science.^40^ They can also help fill in gaps in conceptual understanding between disciplines.^41^ Finally, case-based activities that connect learning goals with real-world applications can increase student performance.^42^

We have developed EMMAs (Exploring Molecular Modeling Activities), which engage high school students in exploring 3D molecular structure, molecular representations, noncovalent interactions, and molecular dynamics through modern biomolecular visualization/simulation technologies and interdisciplinary content connections. The series of activities reinforces each of these topics as students explore drug–target interactions in the context of a fictitious “case study” of a patient with chronic myeloid leukemia while also gaining skills in using the Visual Molecular Dynamics (VMD) software package. Each of the activities is discussed in more detail below. These activities were implemented with multiple sections of high school chemistry students, and pre- and postactivity surveys including both Likert response type and free-response items were implemented to assess how student understanding of intermolecular interactions and molecular dynamics, perceived comfort with molecular visualization, and recognition of science as interdisciplinary changed after engaging with the activities. We also queried student satisfaction and interest in completing future activities to consider the level of student engagement and potential frustrations with the activities. In this work, we describe the development, implementation, and assessment of these activities as well as an analysis of the resulting assessment data.

## Description of Activities

**Figure 1:**
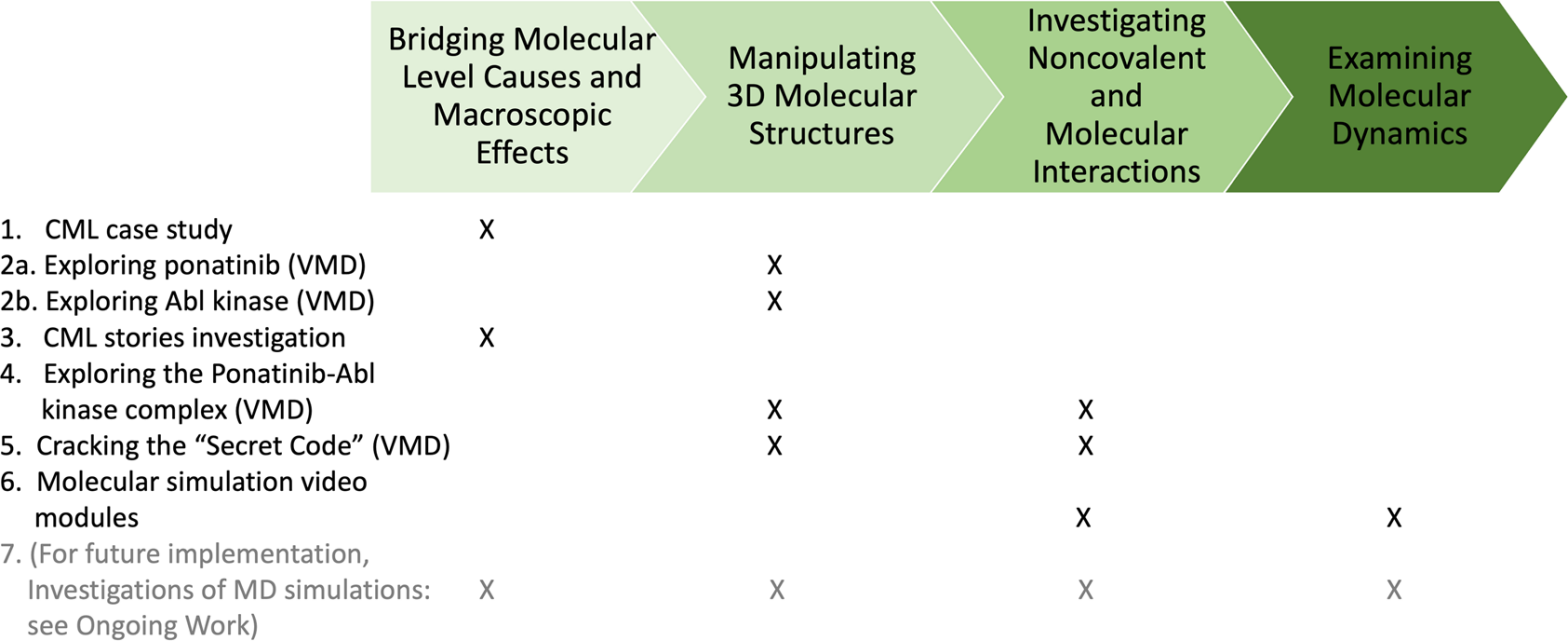
Summary of the series of activities within the EMMAs. There are 6 activities in total (with a 7th activity developed for future implementation). Each activity is listed under its corresponding aim(s), with the aims shown in progression at the top.

Figure 1 shows a flowchart of the suite of activities that comprise the EMMAs. Activities were designed to mesh with both Massachusetts state standards and the Next Generation Science Standards (NGSS), which is a set of standards developed in a collaboration between 26 states, the National Research Council (NRC), the National Science Teachers Association (NSTA), and the American Association for the Advancement of Sciences (AAAS). For example, the considerations of intermolecular interactions that connect the activities reflect aspects of NGSS standards HS-PS1–3, such as Structure and Properties of Matter (PS1.A) and Types of Interactions (PS2.B). The activities also involve elements that bridge multiple levels of Bloom’s Taxonomy (remember, understand, apply, analyze, and evaluate), as shown in their associated learning goals (Table 1). In the Supporting Information, we provide the full text of activities (Files 00–07 and A–B), additional details about their alignment to learning standards (File D), learning goals with associated levels of Bloom’s Taxonomy (File C), and computational details related to the creation of files for activities (File II). The final versions of these activities described here were the result of an iterative design process in which they were first introduced during a pilot study in Spring 2022 and refined based on the observations of the implementing instructor (MB) and student responses.

**Table 1:** Summary of the Primary Learning Goals for Molecular Visualization and Molecular Dynamics Based Activities Described in this Work

Activities	Learning goals
1. CML Case Study	• Explain how a genetic mutation leads to a change in protein function.
	• Explain how a drug molecule, through physically interacting with a target, can alter the target’s function.
	• Explain how a biomolecular change can lead to a disease.
2. Exploring ponatinib and Abl kinase	• Recall that proteins are polymers of amino acids that can form secondary and tertiary structures.
	• Recognize that atoms are the basic building blocks of molecules.
	• Know how to rotate, translate, and zoom in/out and center the molecules of interest.
	• Compare and contrast different coloring and drawing methods in VMD.
	• Select relevant parts of the molecules using VMD.
	• Determine the distances between atoms using VMD.
	• Compare and contrast the value of using certain graphical representations to answer relevant questions.
	• Identify molecular geometries visually.
3. CML stories investigation	• Provide an example of a person with CML and summarize some of their experiences (symptoms, diagnosis, etc.)
	• Explain the connection between biomolecular processes and changes and disease.
	• Reflect and comment on aspects of the athlete’s story that make a personal impression on them.
4. Exploring the ponatinib–Abl kinase complex	• Manipulate the molecule through rotation, translation, and zooming in/out in order to answer other biochemical questions.
	• Compare and contrast the different drawing methods in VMD in the context of visualization.
	• Select relevant parts of the molecules using VMD.
	• Determine the distances between atoms using VMD.
	• Create multiple representations to visualize multiple molecules simultaneously.
	• Evaluate choices of representations when visualizing molecular interactions.
	• Identify hydrogen bonding within the ponatinib–Abl kinase complex.
5. Cracking the “secret code”	• Compare and contrast the different coloring and drawing methods in VMD.
	• Use VMD commands to aid in molecule selection.
	• Illustrate the difference between graphical representations by creating multiple representations.
	• Recognize amino acids.
	• Connect amino acid structure with their physical and chemical properties.
	• Identify hydrogen bonding within the ponatinib–Abl kinase complex.
	• Recognize important kinase residues that interact closely with the drug.
	• Determine the distance between two atoms.
	• Recall what constitutes covalent bonds, hydrogen bonding interactions, and hydrophobic interfaces.
	• Describe the structure of amino acids.
	• Explain the importance of biological interactions, both covalent and noncovalent, in protein–drug interactions.
6. Molecular simulation video modules	• Describe what an MD simulation is and what it shows.
	• Outline the important steps in creating an MD simulation.
	• Articulate the importance of MD simulations in biological systems.
	• Relate MD simulations to a biological system outside of those discussed.
	• Explain applications of Newton’s Laws to create an MD simulation.
	• Articulate basic physical concepts relating to electrostatic interactions and force.
	• Explain the importance of small time steps when creating a trajectory.
7. Investigations of MD simulations	• Summarize the significance of MD simulations in scientific research.
	• Apply VMD skills to a dynamic trajectory.
	• Predict how MD simulations can be used in drug design.
	• Compare and contrast the structural and binding aspects of ponatinib and imatinib.
	• Generate hydrogen bonding graphs.
	• Analyze hydrogen bonding graphs.
	• Analyze distance and fluctuation analysis graphs.
	• Justify the importance of data analysis in understanding drug–target interactions.
	• Recognize the dynamic nature of molecules.
	• Explain ways that science is interdisciplinary.
	• Identify the purpose and main findings from a primary literature article abstract.
	• Explain how science is an ongoing process.
	• Evaluate and weigh different and sometimes contradictory evidence in addressing scientific questions.

### Case Study

An initial case study was developed that introduced students to a patient with chronic myeloid leukemia (CML), with the goal of helping students see the connections between molecular-level changes and human-related macroscopic consequences. The case study first describes the clinical presentation and then explains the genetic underpinnings of the disease. It then introduces imatinib (Gleevec),^43,44^ a drug developed to competitively bind to the ATP-binding pocket of the Bcr–Abl kinase to treat CML. Two EdPuzzles (San Francisco, CA), linked to external resources, were created to accompany this case study. The first EdPuzzle links to a Khan Academy video^45^ that introduces students to CML. The second EdPuzzle links to a Howard Hughes Medical Institute (HHMI) BioInteractive video^46^ describing how imatinib physically inhibits the Bcr–Abl kinase. The web site associated with this resource also contains template files from which 3D models of ATP, imatinib, and the Abl kinase domain of Bcr–Abl kinase were 3D printed to use as tangible manipulatives in our classroom implementation.

### Introduction to VMD—Exploring Ponatinib and Abl Kinase Separately

In this two-part activity, students learn to use VMD as they first explore ponatinib,^47,48^ a drug molecule that treats CML via the same mechanism as imatinib, and subsequently consider its target protein domain Abl kinase through step-by-step, guided instructions and prompts. Ponatinib was chosen here rather than imatinib because it had a greater diversity of molecular geometries and atom types (e.g., a linear alkyne motif and fluorine atoms). For details on preparation of the drug/protein structure, see File II of the Supporting Information. The first part of this activity with ponatinib provides a scaffolded approach to guide students through learning how to manipulate (e.g., rotate, translate, zoom) a molecular system, change representations (e.g., ball-and-stick vs space-filling vs lines), and determine molecular-level distances using VMD. During this portion, students are asked questions that enable them to compare 2D vs 3D representations and reflect on the strengths and weaknesses of various representations. A challenge portion at the end of the first part asks students to identify central atoms within ponatinib with particular molecular geometries.

In the second part, students continue to build VMD skills by manipulating and exploring different representations and aspects of a protein molecule (the Abl kinase). This part also aims to reinforce and/or introduce basic concepts in protein structure and to demonstrate how molecules can be comprised of both fewer *and* larger numbers of atoms. Students are instructed to selectively show certain residues within the protein and challenged to identify them using an accompanying reference sheet showing all 20 amino acids. The two parts can be completed within one ∼75 min block or within two shorter class blocks. Prior to completing the second portion of the activity, students can refresh their knowledge of protein structure from a prior high school biology course with a mini-lesson as appropriate (as was done in our implementation).

### CML Stories Investigation

In this activity, students can further connect molecular-level processes with significant macroscopic consequences by choosing one of four athletes with CML to investigate. They close-read an article or watch a video interview to answer a series of questions. The activity seeks to engage students in learning about scientific topics through real-life stories. Student choice is a powerful tool for engagement, and in the activity, students can select an athlete’s story that interests them. Conceptually, the activity supports the case-study narrative, allowing more students an alternative entry point into engaging with challenging particulate-level science.

### Exploring the Ponatinib/Abl Kinase Complex

In this activity, students examine the bound state ponatinib/Abl kinase complex, exploring ways to represent each molecule and evaluating why it might be important to use different representations for each. In carrying out this activity, students see the drug binding within a pocket in the kinase and may appreciate the role of shape complementarity in this interaction. As a challenge activity, students are asked to apply their understanding of noncovalent interactions to identify and display four distinct hydrogen bonds formed between the drug and the target.

As tangible materials to accompany this activity, students in our implementation were also presented with 3D-printed models of a kinase protein, a drug molecule (in this case, imatinib), and an ATP molecule to reinforce ideas of shape complementarity and physical interactions in drug design. These models were created using freely available template files.^46^

### Cracking the “Secret Code”

**Figure 2:**
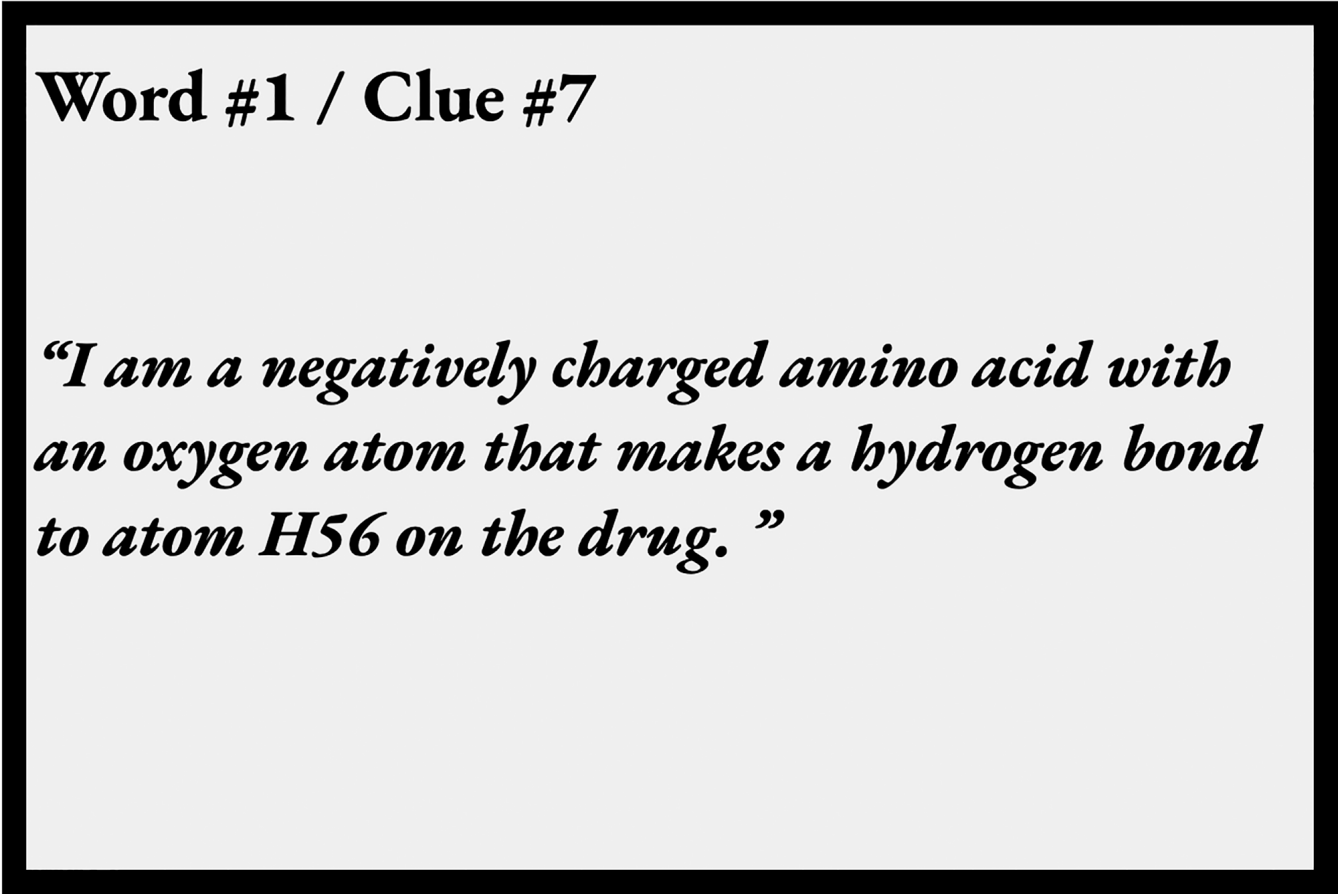
Sample clue card for the “Cracking the Secret Code” activity in which students use VMD to identify mystery amino acids in the ponatinib/Abl kinase complex, whose one-letter codes can be unscrambled to yield a message.

Students continue to practice their skills with molecular manipulation, representation, labeling, selection, and distance analysis using VMD by identifying elements of biomolecular structure and noncovalent (and covalent) interactions within a protein–drug complex to decipher a secret message using a series of clues. Each clue requires the student(s) to use VMD to identify an amino acid within the Abl kinase protein, and the one-letter amino acid code for that letter is the “solution” to the clue. The result of all of the clues can be unscrambled to yield a message. A sample clue is shown in Figure 2. This activity can be implemented in many ways, depending on the level of students. For students who need the most scaffolding, the teacher can guide the class through each clue. A less scaffolded version of this activity involves students or groups of students, each taking one or more “clue cards” and contributing one or more letters to the message that the class can collectively decode. A still less scaffolded version involves a Web site in which students can individually or collectively engage with as few or as many clues as they can or wish.

### Molecular Simulation Video Modules

We wished to augment students’ understanding of 3D molecular structure by reinforcing the idea that molecules are always dynamic, not static. To that end, we developed two brief (∼3–6 min) videos showing molecular dynamics (MD) simulation trajectories of the chronic myeloid leukemia system–Abl kinase bound to imatinib^49^ discussed in the earlier case study. These videos highlight the importance of molecular motion and explain the basic physical and mathematical ideas behind MD simulations, in which Newton’s Laws of motion in physics are numerically integrated to predict and simulate molecular motion over time, explicitly showcasing their cross-disciplinary nature.

## Study and Implementation Details

### Implementation of Activities and Relevant Student Population

Activities 1–6 listed above were carried out in Spring 2023 with students in chemistry classes from a four-year public high school located in Massachusetts. This high school had an enrollment of approximately 1500 students. After graduation, 90% of the students in the class of 2021 enrolled in a four-year college and 3% in a two-year college, while others’ plans included college prep, employment, and military service.

The school’s core science curriculum consists of a four-year sequence of earth science, biology, chemistry, and physics. All tenth graders are required to take a course in biology. Although not required, students typically continue into chemistry the following year after biology. At the school, there are four options for students to engage with chemistry primarily during their junior year: Accelerated Chemistry, Chemistry 1, Chemistry 2, and Practical Chemistry. Advanced Placement (AP) chemistry, which is generally equivalent in level and coverage to a college general chemistry course, is offered as an elective to seniors only after completing a first year chemistry course.

The chemistry students who carried out these activities were enrolled in either Chemistry 1 or Chemistry 2. Chemistry 1 meets for approximately 4 h per week, and students engage in inquiry-based learning, lab and discussion, problem-solving, and analysis. In Chemistry 2, class sizes are smaller (≈16 vs ≈24), pacing is slower, and more scaffolding and learning supports are provided than in Chemistry 1. Both courses generally cover the same topics over the course of the year, including the topics related to the activities in this study. Two sections of each course participated in these activities.

Activities 1–6 were carried out over the course of approximately a week near the end of the academic year after students had been introduced via their standard curriculum to relevant chemical concepts such as molecular geometries and intermolecular forces. Students worked in pairs or groups of three on activities 2, 4, and 5 and carried out activities 1 and 3 as homework. For activity 6, students worked through the EdPuzzle during class time independently and with whole class discussion.

### Assessment Implementation

For this study, we utilized an iterative process to develop a survey to assess learning gains and changes in the perceptions of science. A preliminary version of this survey incorporating Likert response type items on student learning experiences adapted from CURE^50^ and SURE^51^ along with items related to molecular motions was first introduced for a small secondary student cohort involved in earlier versions of molecular dynamics activities.^52^ The length of this survey proved difficult for reliable student completion, leading us to significantly revise it to focus on items most related to molecular behavior and the learning goals of activities related to visualizing molecules. We also added free-response written and molecular drawing questions to provide more complete insight into students’ perceptions of intermolecular interactions. Supplementing written responses with drawn images has been a useful approach to characterize student conceptualization of intermolecular forces in past work.^5,29^ The postsurvey also included questions related to student satisfaction, including free-response opportunities for students to express positive aspects of the activities and areas for improvement. In Spring 2022, this survey version was then utilized in a pilot study with an initial version of the activities described here. Our analysis of pilot study results led us to further refine questions to ensure that terminology in items was clear to our student population. The full text of final pre- and postsurveys utilized in this study is provided in the Supporting Information.

While all students in these sections of chemistry classes participated in activities, only students who had parental consent to participate in this study were included in analyses. Surveys were administered to students before and after completion of the suite of activities through the Qualtrics platform. Across the classes, 43 students with parental consent submitted both pre- and postsurveys. The demographics of student participants are given in Table 2 and are generally representative of the overall student population in the high school in terms of gender, racial/ethnic background, and previous and concurrent science courses. The study was determined to be IRB-exempt by the Institutional Review Board overseeing research at Wellesley College (IRB #22063R-E); participation of all students enrolled in the course with appropriate parental consent was approved. Student pre- and postsurvey responses were matched via a codename. Students chose their codename and provided it directly to a school administrator. This administrator was the only individual with access to the list of student names and associated codenames. The administrator provided the researchers with a list of the codenames for students who had parental consent to participate in the study. The file connecting actual student names to codenames was destroyed after completion of the surveys to protect student responses.

**Table 2:** Demographics of Students Who Completed Both Pre- and Postsurveys

Student characteristic	Number (percentage of sample)^a^
*Gender*	
Female	22 (51%)
Male	18 (42%)
Nonbinary/other	2 (5%)
*Race/ethnicity*^b^	
American Indian/Alaskan Native	2 (5%)
Asian-American	4 (9%)
Black/African-American	3 (7%)
Filipino	2 (5%)
Hawaiian/Pacific Islander	0 (0%)
Hispanic/Latino	3 (7%)
White	34 (80%)
*Class year*	
Junior	42 (98%)
*Current chemistry course*	
Chem 1	28 (65%)
Chem 2	15 (35%)
*Other previous or current high school science courses*^b^	
Anatomy and physiology	3 (7%)
Biology	43 (100%)
Environmental science	7 (16%)
Forensics	1 (2%)
Physics	1 (2%)
Other science	11 (26%)

a Data for 43 students; in some cases individual students may have chosen not to answer a particular demographic question leading to *n* < 43 in table.

b Students could choose more than one category.

Student responses were compared between pre- and postsurveys. Only students who had both pre- and postsurvey responses to a particular question were included for analysis. For Likert response type questions, mean pre- and postresponses were compared using Wilcoxon signed rank tests. Open-response questions with text or drawing responses were analyzed using a constant comparative type approach.^53^ First, pre- and postresponses were compiled together in a random order so scorers were unaware of whether a given response was submitted before or after activities were completed. Two investigators (MLR and DEE) did independent initial reads of responses and, based on that initial reading, independently proposed coding categories for responses. The investigators then met to discuss and formalize the names for categories as provided in Tables 4–6. They then independently scored whether each response included aspects related to each category; a given response could potentially correspond to more than one category. After this scoring, the two investigators met again to discuss any responses where their scores differed to determine a final consensus scoring. After this process, student pre- and postsurvey responses were compared for each scoring category using Fisher’s Exact Test with 2 × 2 contingency tables. All statistical analyses were performed using SPSS version 28.0.1.1.

**Table 3:** Comparison of Mean Pre- and Postsurvey Responses of Students to Likert Response Type Questions^a^

Question^b^	Presurvey mean (SD)^c^	Postsurvey mean (SD)^c^	*p*-value^d^
I understand different ways to visualize a molecule	1.88	1.35	<0.001**
	(0.697)	(0.529)	
Molecules interact with each other	1.51	1.33	0.029*
	(0.592)	(0.606)	
Molecules are not always moving	3.51	3.81	0.430
	(1.2)	(1.468)	
I can picture molecules interacting in my mind	2.37	1.95	0.002**
	(0.787)	(0.899)	
The shapes of molecules do not impact how they interact with each other	4.14	4.12	0.774
	(1.146)	(1.258)	
How well two molecules interact with each other can be influenced by the location of their charges	1.81	1.56	0.034*
	(0.764)	(0.548)	
I can explain how a drug molecule and its target molecule interact using pictures, words, or other representations	3.05	1.98	<0.001
	(1.09)	(0.963)	
I get personal satisfaction when I solve a scientific problem by figuring it out myself	2.12	1.84	0.014*
	(0.731)	(0.754)	
I consider myself a science person	2.44	2.4	0.591
	(0.934)	(0.929)	
Evaluate your overall sense of satisfaction with the molecular modeling activities by choosing one statement below^e^		1.83	N/A
		(0.692)	
Would you be interested in completing other molecular modeling activities in the future?^f^		1.73	N/A
		(0.549)	

a Analyses included pre- and postsurvey responses for 43 students.

b Unless otherwise noted student responses to each question were 1: strongly agree; 2: agree; 3: neither agree nor disagree; 4: disagree; 5: strongly disagree.

c Standard deviation values presented in parentheses.

d *p*-values from Wilcoxon signed rank test. * *p* < 0.05; ** *p* < 0.10.

e Student replies were 1: very satisfied; 2: satisfied; 3: neither satisfied nor dissatisfied; 4: dissatisfied; 5: very dissatisfied.

f Student replies were 1: very interested; 2: interested; 3: not interested.

**Table 4:** Comparison of Pre- and Postsurvey Responses of Students to the Prompt, “In no more than three sentences describe how you think a drug molecule and a target molecule can interact with one another.”^a^

Scoring category^b^	Number (%) of presurvey responses	Number (%) of postsurvey responses	*p*-value^c^
Refers to shape complementarity/fit between molecules	6 (16%)	18 (49%)	0.006**
Refers to charge, electrostatics, or intermolecular forces between molecules	6 (16%)	13 (35%)	0.109
Refers to molecules being attracted to or repelling one another	5 (14%)	4 (11%)	1.000
Refers to molecules bouncing, colliding, or touching	3 (8%)	2 (5%)	1.000
Refers to molecules reacting or molecular changes	4 (11%)	0 (0%)	0.115
Conflating of covalent bonds and intermolecular interactions	7 (19%)	5 (14%)	0.754
Refers to aspects of drug function	11 (30%)	14 (38%)	0.624

a Analyses included pre- and post-survey responses for 37 students.

b Each pre- and postsurvey response was independently scored according to whether or not it reflected each scoring category by two investigators. Responses were evaluated in a random order, and all discrepancies were discussed by the investigators after scoring.

c *p*-values from Fisher’s Exact Tests of 2 × 2 contingency tables comparing pre- versus postresponse scoring. * *p* < 0.05; ** *p* < 0.10

**Table 5:** Comparison of Pre- and Postsurvey Responses of Students to the Prompt, “Draw a sketch or picture in the box below of how you think a drug molecule and a target molecule can interact with one another.”^a^

Scoring category^b^	Number (%) of presurvey responses	Number (%) of postsurvey responses	*p*-value^c^
Shows more than one distinct object	37 (95%)	38 (97%)	1.000
Drawing has shape beyond a simple circle	28 (72%)	37 (95%)	0.013*
Depicts ball and stick/Lewis structures	8 (21%)	2 (5%)	0.087
Shows shape complementarity/molecular fit	19 (49%)	33 (85%)	0.002**
Shows charges or charge distributions consistent with interaction	3 (8%)	6 (15%)	0.481
Shows symbol for noncovalent interaction (e.g., dashed line)	8 (21%)	1 (3%)	0.029*
Shows motion/forces between molecules (e.g., arrows)	9 (23%)	5 (13%)	0.377
Drawing integrates drug function	4 (10%)	2 (5%)	0.675

a Analyses included pre- and postsurvey responses for 39 students.

b Each pre- and postsurvey response was independently scored according to whether or not it reflected each scoring category by two investigators. Responses were evaluated in a random order, and all discrepancies were discussed by the investigators after scoring.

c *p*-values from Fisher’s Exact Tests of 2 × 2 contingency tables comparing pre- versus postresponse scoring. * *p* < 0.05; ** *p* < 0.10.

**Table 6:** Comparison of Pre- and Postsurvey Responses of Students to the Prompt, “Describe one way in which understanding how two molecules interact relates to topics in math or other sciences, such as biology or physics.”^a^

Scoring category^b^	Number (%) of presurvey responses	Number (%) of postsurvey responses	*p*-value^c^
Mentions biology	19 (53%)	16 (44%)	0.638
Provides specific example of connection to biology	9 (25%)	11 (31%)	0.793
Mentions math or physics	6 (17%)	15 (42%)	0.037*
Provides specific example of connection to math or physics	5 (14%)	9 (25%)	0.372
Mentions other science (e.g., environmental science)	2 (6%)	0 (0%)	0.493
Provides specific example of other science (e.g., environmental science)	1 (3%)	0 (0%)	1.000
Relates to drugs/drug design	1 (3%)	8 (22%)	0.028*
Expresses “all things made of molecules”	5 (14%)	4 (11%)	1.000

a Scoring categories for responses with statistically significant differences (*p* < 0.05) between pre- and postresponses highlighted in yellow. Analyses included pre- and postsurvey responses for 36 students.

b Each pre- and postsurvey response was independently scored according to whether or not it reflected each scoring category by two investigators. Responses were evaluated in a random order, and all discrepancies were discussed by the investigators after scoring.

c *p*-values from Fisher’s Exact Tests of 2 × 2 contingency tables comparing pre- versus postresponse scoring. * *p* < 0.05; ** *p* < 0.10

## Results and Discussion from Assessment

### Evolution of How Students’ Conceptualize Intermolecular Interactions

Students showed interesting progressions in how they articulated and conceptualized molecular representations and intermolecular interactions in their responses to survey questions before and after completing the activities. In particular, students demonstrated an increased confidence in their ability to visualize molecules and explain interactions between molecules. Students had significantly higher levels of agreement in the postsurvey with three statements: “I understand different ways to visualize a molecule” (*p* < 0.001), “I can picture molecules interacting in my mind” (*p* = 0.002), and “I can explain how a drug molecule and its target molecule interact using pictures, words or other representations” (*p* < 0.001) (Table 3). This increased comfort with considering intermolecular interactions was also apparent in their replies to open-ended prompts asking them to explain drug–target interactions in either a short written response or a drawing. Both student written responses and drawings became notably more “sophisticated” in their descriptions of these interactions, particularly in expressing the importance of shape complementarity in molecular interactions (Tables 4 and 5).

Student written descriptions were given to the prompt, “In no more than three sentences describe how you think a drug molecule and a target molecule can interact with one another.” The percentage of responses to this prompt incorporating shape complementarity increased dramatically from 16% of responses in the presurvey to 49% of responses in the postsurvey (*p* = 0.006) (Table 4). A few representative examples of the progression students showed between their pre- and postactivity responses are seen here:

- Preactivity: They can attract or repel each other based on their charges.
- Postactivity: These two molecules have opposite charges and can fit into one another.

- Preactivity: I think the drug molecule will be attracted to the target molecule and one will destroy the other.
- Postactivity: I think that the drug molecule will fit into the target molecule. Also, I believe if the target has a positive charge the drug will have negative charges attracting to it.

- Preactivity: The drug forms strong bonds with target molecules.
- Postactivity: The drug molecule goes to the target molecule, and it is a shape that matches with an opening/indentation/thing in the target.

**Figure 3:**
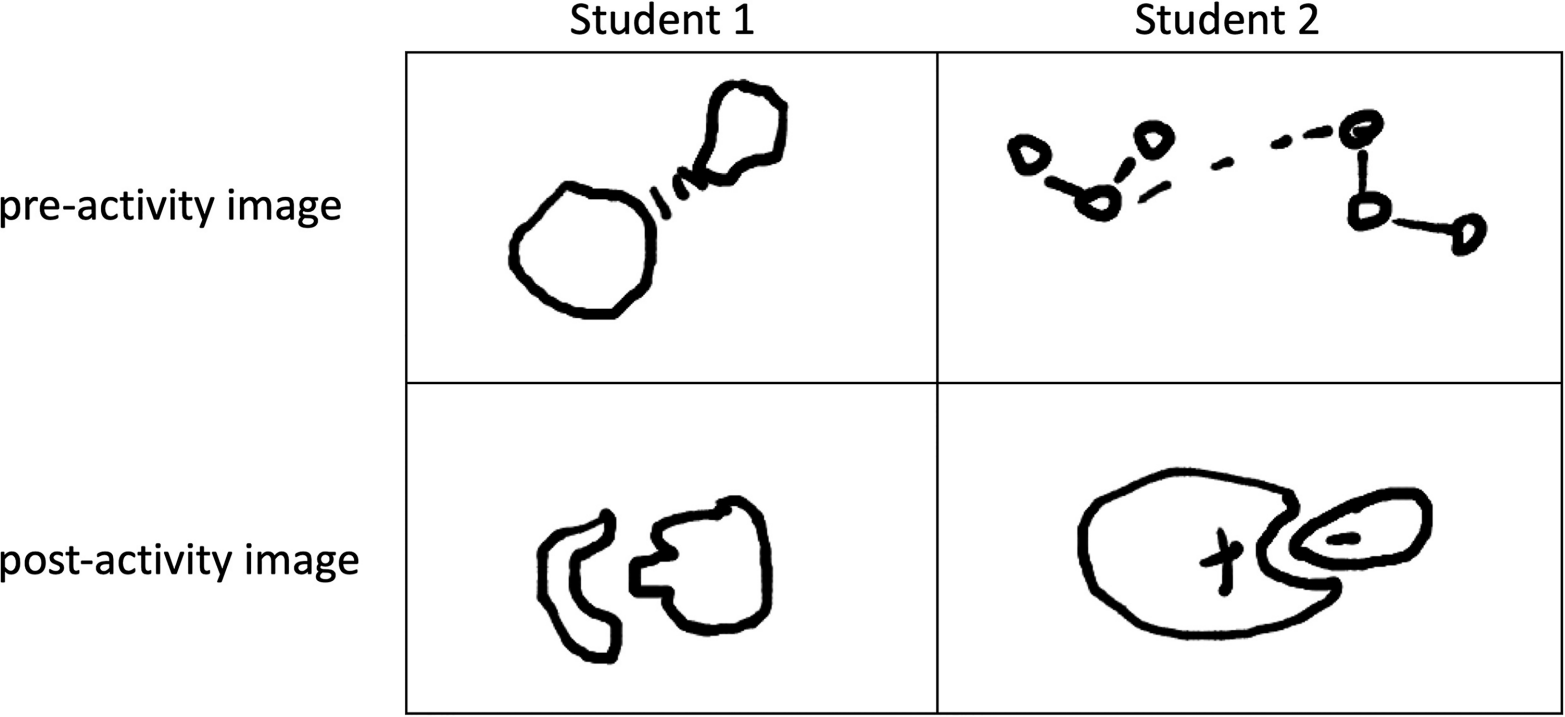
Representative pre- and postactivity images drawn by two students in response to the prompt, “Draw a sketch or picture in the box below of how you think a drug molecule and a target molecule can interact with one another.”

A similar progression was seen in student responses to the prompt, “Draw a sketch or picture in the box below of how you think a drug molecule and a target molecule can interact with one another.” As in written responses, significantly more drawings incorporated aspects of shape complementarity in the postactivity responses (85%) compared to those in preactivity responses (49%) (*p* = 0.002) (Table 5). In addition to the increased incidence of shape complementarity, we also observed a decrease from 21% to 3% of students using a “symbol”, such as a dashed line, to imply interactions in their drawings (*p* = 0.029). Figure 3 shows representative drawings provided by students that capture these types of transitions.

Interestingly, the Likert response type question related to shape complementarity (“The shapes of molecules do not impact how they interact with each other”) did not show any significant difference between pre- and postsurvey responses (Table 3) despite the clear and consistent progression seen in free responses. We hypothesize that this may have occurred because of student confusion related to the “negative” wording of this question designed as a reverse score item or because of inattentional blindness resulting from students focusing on the conceptual portion of the statement and not the presence of “not” in the item. The standard deviations of student responses were the highest for both negatively phrased Likert response type questions (this item and “Molecules are not always moving”), implying that the wording of the questions may have led to one of these effects. We would reconsider this aspect of our survey design before its use in subsequent studies.

While the progression in student responses was most pronounced in terms of considering intermolecular shape complementarity, there was also some evidence that students increased their consideration of electrostatics over the course of the activities. This was seen in part by the significantly greater student agreement with the statement, “How well two molecules interact with each other can be influenced by the location of their charges” (*p* = 0.034) (Table 3). Moreover, written responses showed an increased incidence of references to electrostatics, shifting from 16% of preactivity responses to 35% of postactivity responses, although this increase only showed borderline significance (*p* = 0.11) in statistical analyses (Table 4). We note that the use of the word “charge” in isolation, without discussing more subtle ideas such as net charge, partial atomic charge, and/or charge distributions, may lead students to have an oversimplified view of molecular interactions, and so it is important for these activities to be carried out in a curricular context in which varied models of conceptualized electronic properties of molecules—each with its limitations—are discussed.

### Student Interdisciplinary Connections

Students also showed an increased appreciation of the connections of intermolecular interactions with physics and mathematics upon activity completion. Prior to the activities, roughly half of the students mentioned a connection to biology when responding to the prompt, “Describe one way in which understanding how two molecules interact relates to topics in math or other sciences, such as biology or physics” (Table 6). While the percentage who mentioned connections to biology did not significantly decrease in the postactivity survey, the percentage of those who mentioned either math or physics significantly increased from 17% to 42% (*p* = 0.037). This increased appreciation for more quantitative or physics-based connections is not surprising given that essentially all students in the class had previously completed a high school biology course, while only a single student had experience with high school physics courses (Table 2). While the connections that students mentioned were often relatively “surface level” and did not necessarily cite a particular physics or mathematics principle or concept, this nonetheless highlights students having an emerging appreciation for those connections over these fairly brief activities. A few examples of how student responses progressed to incorporate these aspects is shown in the representative pre- and postactivity responses below:

- Preactivity: Applies to environmental science in how water does not mix with nonpolar oil.
- Postactivity: The way two molecules interact relates to how they are governed by the laws of physics and chemistry.

- Preactivity: Relates to like early sciences/all other sciences because basically everything is made up of molecules, it is integral to know how the world works and why/how things happen.
- Postactivity: It relates to those things because the interaction of the molecules is influenced by things like math with the timesteps and physics with the laws, etc.

- Preactivity: I could see it being useful in biology for interactions of molecules in the body to explain functions of the body in nature and humans.
- Postactivity: It involves forces which are seen in physics.

The focus on drug-related intermolecular interactions in the activities also led more students to make explicit connections with aspects of medicinal chemistry or drug design in their postactivity responses to this prompt. In fact, roughly one-third of students made these connections in their postactivity responses, compared to a single student before the activity (*p* = 0.028) (Table 6). A few representative examples of student responses evolving to incorporate aspects related to drug design were:

- Preactivity: Knowing how two molecules interact can explain proteins or enzymes react [sic].
- Postactivity: Understanding how molecules interact can relate to someone who is taking any sort of medication and how that reacts with the body.

- Preactivity: Everything living around us is made up of molecules, so biology directly relates to the interactions between molecules.
- Postactivity: Seeing how two molecules interact can help cure diseases that were known as incurable. The case we learned about was cancer.

- Preactivity: To study many topics in Biology like genetics and anatomy, one must have a decent knowledge of molecules and how they interact. For example, in genetics, AT and GC pairs fit together in that way because of the way their molecules interact. If you know that, you will better understand DNA and that sort of thing.
- Postactivity: The creation of new drugs requires a knowledge of how molecules interact with each other. For drugs that are inhibitors, the knowledge of the specific way that a molecule fits in with another molecule is very necessary for the drug to work properly.

### Student Satisfaction

While student satisfaction does not necessarily correlate to student learning,^54^ we were interested in assessing whether students had a positive experience performing activities with research-grade software that could have a potentially frustrating learning curve. Overall, students in both the Chemistry 1 and 2 courses appeared to be generally satisfied with their participation in these activities (Table 3). Over 90% of students reported that they found the experience satisfying or very satisfying on their postactivity survey, and 95% said that they would be very interested or interested in participating in a similar experience again.

In a postsurvey free response question asking students to note their favorite aspect of the activities, a majority (53%) specifically appreciated the ability to visualize molecules in different ways, particularly in three dimensions. The most common suggestions for improving the activities related to challenges in learning or using the visualization software (23% of students), which emphasized the necessity of making sure students have computers with the necessary software preinstalled and incorporating sufficient time to answer technical questions as they arise. While not a direct measure of satisfaction, we also found it heartening that students reported an increased agreement with the statement “I get personal satisfaction when I solve a scientific problem by figuring it out myself” after the activity (Table 3). In future work it would be interesting to further consider whether learning how to use molecular visualization software to explore molecules on their own might increase students’ sense of confidence in approaching chemical questions.

## Ongoing Work

Our goal is for these activities to be flexibly and easily adaptable for a variety of high school classrooms. Their linear progression creates opportunities for teachers to carry out any subset of the full progression depending on their preferences and constraints of limited class time. For example, students can carry out only Activity 1 along with the first half of Activity 2 (using VMD to manipulate ponatinib) in less than a class period while still enabling students to explore 3D molecular structure and gain exposure to using molecular visualization software. Alternatively, students can carry out activities 1–4 over roughly two class periods, potentially with Activities 1 and/or 3 assigned as homework.

These activities can also create opportunities for explicit in-class bridges between traditionally “siloed” high-school courses. For example, teachers from biology, physics, math, and even computer science can leverage aspects of these activities in collaboration with a chemistry teacher to showcase the connections to their disciplines, e.g., the genetic and protein regulatory aspects of CML (biology), the quantification of forces, accelerations, and velocity (physics), the calculus being demonstrated by numerical integration (math), and even brainstorming code elements needed for the implementation of an MD simulation (computer science). We look forward to broadening both the involved student and teacher populations in ongoing development and assessment of this suite of activities.

As we work toward broadening the adoption of these activities, one goal is to make their implementation as easy as possible for an instructor unfamiliar with the VMD software package. Although students in our cohort mostly reported positive experiences with VMD, we appreciate the potentially steeper learning curve for research-grade software compared to other packages, such as JMol or Mol*.^36^ The implementation of the activities in visualization software, such as MolView, which can be used on the Chromebook platform used in many schools, would also promote a broader use of these activities. Current work also aims to create more differentiated forms of the activities, including those geared toward students in a second-year high school chemistry course (i.e., Advanced Placement or International Baccalaureate Higher Level) or an introductory undergraduate level.

We have also developed a final activity (Activity 7 in parentheses in Figure 1; files prefixed with “07...” in the Supporting Information), through which students further explore and analyze MD simulations. Students use VMD to visualize previously generated MD simulation trajectories of (1) imatinib bound to wild-type Abl kinase and (2) imatinib bound to a mutant kinase known to cause significant imatinib drug resistance.^55^ The students then use VMD to generate data that enable them to compare hydrogen bonding patterns, drug/target distance metrics, and other structural metrics between the two systems to generate hypotheses about why drug resistance might occur. The activity leads the student to better understand the basis for the development of ponatinib to specifically combat resistance, and it engages students with an abstract from primary literature^56^ to help students see science as an ongoing, evolving dialogue. We are eager to implement this latest activity with students as a “capstone” in future work.

## Conclusions

In this work, we introduce and assess a suite of molecular modeling activities through which high school students learn to use modern molecular visualization software and enhance their understanding of molecular structure and interactions while gaining an awareness of interdisciplinary, applied science. We demonstrate that these activities significantly improved students’ perceived abilities to visualize molecules, changed their articulated understanding of molecular interactions to become more specific and/or sophisticated, and increased their awareness of molecular science involving math and physics in addition to chemistry and biology. We are eager to continue their ongoing development and partner with additional high school educators to broaden their effectiveness and to increase their accessibility and flexibility.

## Supporting Information Available

Files 00–07 contain the content associated with all the activities and include a zipped folder containing the structural .pdb files. Files prefixed with A–B are helpful resource sheets for students carrying out the activities. Files prefixed with C–D contain information about learning goals and related/applicable regional/national standards. Files prefixed with I, II, and III contain the survey instruments, the computational details behind the creation of structural files used, and SPSS output from statistical analyses, respectively. Trajectory files from MD related activities that were too large for supporting information can be obtained via links in documents or by email request from the authors. (ZIP)
