# Supplementary material for: EMMAs: Implementation and Assessment of a Suite of Cross-Disciplinary, Case-Based High School Activities to Explore Three-Dimensional Molecular Structure, Noncovalent Interactions, and Molecular Dynamics
Source: J Chem Educ. 2024 May 10;101(6):2436–47. doi: 10.1021/acs.jchemed.4c00036 (PMC11171454; doi:10.1021/acs.jchemed.4c00036)
Supplement: Supplementary file 1 — ed4c00036_si_001.zip [file ed4c00036_si_001.zip › Kotsalidis_supporting_info_revisions/III - SPSS Output for Kotsalidis et al.pdf]

Nonparametric Tests

Notes

Output Created		28-FEB-2024 15:05:29
Comments		
Input	Data	/Users/delmore/Documents/Research/Molecular Dynamics HS Project/Spring 2023 Data Analysis/Spring 2023 LSHS merged data for demo.sav
	Active Dataset	DataSet1
	Filter	<none>
	Weight	<none>
	Split File	<none>
	N of Rows in Working Data File	99
Syntax		NPTESTS /RELATED TEST (Perception_questions_1 Perception_questions_1_post) /MISSING SCOPE=ANALYSIS USERMISSING=EXCLUDE /CRITERIA ALPHA=0.05 CILEVEL=95.
Resources	Processor Time	00:00:01.24
	Elapsed Time	00:00:01.00

```
[DataSet1] /Users/delmore/Documents/Research/Molecular Dynamics HS Project/Spring 2023 Da
ta Analysis/Spring 2023 LSHS merged data for demo.sav
```

Hypothesis Test Summary

	Null Hypothesis	Test	Sig.[a,b]
1	The median of differences between In this section we present questions about science and questions about you. These will help us put learning in context. For each item below please rate your agreement with the item: - I understand different ways to visualize a molecule. and In this section we present questions about science and questions about you. These will help us put learning in context. For each item below please rate your agreement with the item: - I understand different ways to visualize a molecule. equals 0.	Related-Samples Wilcoxon Signed Rank Test	<.001

Hypothesis Test Summary

	Decision
1	Reject the null hypothesis.

a. The significance level is .050.

b. Asymptotic significance is displayed.

Related-Samples Wilcoxon Signed Rank Test

In this section we present questions about science and questions about you. These wi ll help us put learning in context. For each item below please rate your agreement wit h the item: - I understand different ways to visualize a molecule., In this section we p resent questions about science and questions about you. These will help us put learni ng in context. For each item below please rate your agreement with the item: - I unde rstand different ways to visualize a molecule.

Related-Samples Wilcoxon Signed Rank Test Summary

Total N	51
Test Statistic	54.000
Standard Error	44.686
Standardized Test Statistic	-3.995
Asymptotic Sig.(2-sided test)	<.001

Nonparametric Tests

Notes

Output Created		28-FEB-2024 15:08:15
Comments		
Input	Data	/Users/delmore/Documents/Research/Molecular Dynamics HS Project/Spring 2023 Data Analysis/Spring 2023 LSHS merged data for demo.sav
	Active Dataset	DataSet1
	Filter	<none>
	Weight	<none>
	Split File	<none>
	N of Rows in Working Data File	99
Syntax		NPTESTS<br>/RELATED TEST (Perception_questions_2 Perception_questions_2_post)<br>/MISSING SCOPE=ANALYSIS USERMISSING=EXCLUDE<br>/CRITERIA ALPHA=0.05 CILEVEL=95.
Resources	Processor Time	00:00:00.46
	Elapsed Time	00:00:01.00

Hypothesis Test Summary

	Null Hypothesis	Test	Sig.[a,b]
1	The median of differences between In this section we present questions about science and questions about you. These will help us put learning in context. For each item below please rate your agreement with the item: - Molecules interact with each other. and In this section we present questions about science and questions about you. These will help us put learning in context. For each item below please rate your agreement with the item: - Molecules interact with each other. equals 0.	Related-Samples Wilcoxon Signed Rank Test	.029

Hypothesis Test Summary

	Decision
1	Reject the null hypothesis.

a. The significance level is .050.

b. Asymptotic significance is displayed.

Related-Samples Wilcoxon Signed Rank Test

In this section we present questions about science and questions about you. These wi ll help us put learning in context. For each item below please rate your agreement wit h the item: - Molecules interact with each other., In this section we present questions about science and questions about you. These will help us put learning in context. Fo r each item below please rate your agreement with the item: - Molecules interact wit h each other.

Related-Samples Wilcoxon Signed Rank Test Summary

Total N	51
Test Statistic	21.000
Standard Error	14.431
Standardized Test Statistic	-2.183
Asymptotic Sig.(2-sided test)	.029

Nonparametric Tests

Notes

Output Created		28-FEB-2024 15:09:26
Comments		
Input	Data	/Users/delmore/Documents/Research/Molecular Dynamics HS Project/Spring 2023 Data Analysis/Spring 2023 LSHS merged data for demo.sav
	Active Dataset	DataSet1
	Filter	<none>
	Weight	<none>
	Split File	<none>
	N of Rows in Working Data File	99
Syntax		NPTESTS<br>/RELATED TEST (Perception_questions_3 Perception_questions_3_post)<br>/MISSING SCOPE=ANALYSIS USERMISSING=EXCLUDE<br>/CRITERIA ALPHA=0.05 CILEVEL=95.
Resources	Processor Time	00:00:00.43
	Elapsed Time	00:00:00.00

Hypothesis Test Summary

	Null Hypothesis	Test	Sig.[a,b]
1	The median of differences between In this section we present questions about science and questions about you. These will help us put learning in context. For each item below please rate your agreement with the item: - Molecules are not always moving. and In this section we present questions about science and questions about you. These will help us put learning in context. For each item below please rate your agreement with the item: - Molecules are not always moving. equals 0.	Related-Samples Wilcoxon Signed Rank Test	.430

Hypothesis Test Summary

	Decision
1	Retain the null hypothesis.

a. The significance level is .050.

b. Asymptotic significance is displayed.

Related-Samples Wilcoxon Signed Rank Test

In this section we present questions about science and questions about you. These will help us put learning in context. For each item below please rate your agreement with the item: - Molecules are not always moving., In this section we present questions about science and questions about you. These will help us put learning in context. For each item below please rate your agreement with the item: - Molecules are not always moving.

Related-Samples Wilcoxon Signed Rank Test Summary

Total N	51
Test Statistic	270.000
Standard Error	47.493
Standardized Test Statistic	.790
Asymptotic Sig.(2-sided test)	.430

Nonparametric Tests

Notes

Output Created		28-FEB-2024 15:09:57
Comments		
Input	Data	/Users/delmore/Documents/Research/Molecular Dynamics HS Project/Spring 2023 Data Analysis/Spring 2023 LSHS merged data for demo.sav
	Active Dataset	DataSet1
	Filter	<none>
	Weight	<none>
	Split File	<none>
	N of Rows in Working Data File	99
Syntax		NPTESTS /RELATED TEST (Perception_questions_4 Perception_questions_4_post) /MISSING SCOPE=ANALYSIS USERMISSING=EXCLUDE /CRITERIA ALPHA=0.05 CILEVEL=95.
Resources	Processor Time	00:00:00.38
	Elapsed Time	00:00:00.00

Hypothesis Test Summary

	Null Hypothesis	Test	Sig.[a,b]
1	The median of differences between In this section we present questions about science and questions about you. These will help us put learning in context. For each item below please rate your agreement with the item: - I can picture molecules interacting in my mind. and In this section we present questions about science and questions about you. These will help us put learning in context. For each item below please rate your agreement with the item: - I can picture molecules interacting in my mind. equals 0.	Related-Samples Wilcoxon Signed Rank Test	.002

Hypothesis Test Summary

	Decision
1	Reject the null hypothesis.

a. The significance level is .050.

b. Asymptotic significance is displayed.

Related-Samples Wilcoxon Signed Rank Test

In this section we present questions about science and questions about you. These wi ll help us put learning in context. For each item below please rate your agreement wit h the item: - I can picture molecules interacting in my mind., In this section we prese nt questions about science and questions about you. These will help us put learning i n context. For each item below please rate your agreement with the item: - I can pictu re molecules interacting in my mind.

Related-Samples Wilcoxon Signed Rank Test Summary

Total N	51
Test Statistic	74.000
Standard Error	41.287
Standardized Test Statistic	-3.124
Asymptotic Sig.(2-sided test)	.002

Nonparametric Tests

Notes

Output Created		28-FEB-2024 15:10:48
Comments		
Input	Data	/Users/delmore/Documents/Research/Molecular Dynamics HS Project/Spring 2023 Data Analysis/Spring 2023 LSHS merged data for demo.sav
	Active Dataset	DataSet1
	Filter	<none>
	Weight	<none>
	Split File	<none>
	N of Rows in Working Data File	99
Syntax		NPTESTS /RELATED TEST (Perception_questions_5 Perception_questions_5_post) /MISSING SCOPE=ANALYSIS USERMISSING=EXCLUDE /CRITERIA ALPHA=0.05 CILEVEL=95.
Resources	Processor Time	00:00:00.39
	Elapsed Time	00:00:00.00

Hypothesis Test Summary

	Null Hypothesis	Test	Sig.[a,b]
1	The median of differences between In this section we present questions about science and questions about you. These will help us put learning in context. For each item below please rate your agreement with the item: - The shapes of molecules do not impact how they interact with one another. and In this section we present questions about science and questions about you. These will help us put learning in context. For each item below please rate your agreement with the item: - The shapes of molecules do not impact how they interact with one another. equals 0.	Related-Samples Wilcoxon Signed Rank Test	.774

Hypothesis Test Summary

	Decision
1	Retain the null hypothesis.

a. The significance level is .050.

b. Asymptotic significance is displayed.

Related-Samples Wilcoxon Signed Rank Test

In this section we present questions about science and questions about you. These will help us put learning in context. For each item below please rate your agreement with the item: - The shapes of molecules do not impact how they interact with one another., In this section we present questions about science and questions about you. These will help us put learning in context. For each item below please rate your agreement with the item: - The shapes of molecules do not impact how they interact with one another.

Related-Samples Wilcoxon Signed Rank Test Summary

Total N	51
Test Statistic	123.500
Standard Error	27.922
Standardized Test Statistic	.287
Asymptotic Sig.(2-sided test)	.774

Nonparametric Tests

Notes

Output Created		28-FEB-2024 15:11:29
Comments		
Input	Data	/Users/delmore/Documents/Research/Molecular Dynamics HS Project/Spring 2023 Data Analysis/Spring 2023 LSHS merged data for demo.sav
	Active Dataset	DataSet1
	Filter	<none>
	Weight	<none>
	Split File	<none>
	N of Rows in Working Data File	99
Syntax		NPTESTS /RELATED TEST (Perception_questions_6 Perception_questions_6_post) /MISSING SCOPE=ANALYSIS USERMISSING=EXCLUDE /CRITERIA ALPHA=0.05 CILEVEL=95.
Resources	Processor Time	00:00:00.39
	Elapsed Time	00:00:01.00

Hypothesis Test Summary

	Null Hypothesis	Test	Sig.[a,b]
1	The median of differences between In this section we present questions about science and questions about you. These will help us put learning in context. For each item below please rate your agreement with the item: - How well two molecules interact with each other can be influenced by the locations of their charges. and In this section we present questions about science and questions about you. These will help us put learning in context. For each item below please rate your agreement with the item: - How well two molecules interact with each other can be influenced by the locations of their charges. equals 0.	Related-Samples Wilcoxon Signed Rank Test	.034

Hypothesis Test Summary

	Decision
1	Reject the null hypothesis.

a. The significance level is .050.

b. Asymptotic significance is displayed.

Related-Samples Wilcoxon Signed Rank Test

In this section we present questions about science and questions about you. These will help us put learning in context. For each item below please rate your agreement with the item: - How well two molecules interact with each other can be influenced by the locations of their charges., In this section we present questions about science and questions about you. These will help us put learning in context. For each item below please rate your agreement with the item: - How well two molecules interact with each other can be influenced by the locations of their charges.

Related-Samples Wilcoxon Signed Rank Test Summary

Total N	51
Test Statistic	60.000
Standard Error	26.175
Standardized Test Statistic	-2.120
Asymptotic Sig.(2-sided test)	.034

Nonparametric Tests

Notes

Output Created		28-FEB-2024 15:12:00
Comments		
Input	Data	/Users/delmore/Documents/Research/Molecular Dynamics HS Project/Spring 2023 Data Analysis/Spring 2023 LSHS merged data for demo.sav
	Active Dataset	DataSet1
	Filter	<none>
	Weight	<none>
	Split File	<none>
	N of Rows in Working Data File	99
Syntax		NPTESTS /RELATED TEST (Perception_questions_7 Perception_questions_7_post) /MISSING SCOPE=ANALYSIS USERMISSING=EXCLUDE /CRITERIA ALPHA=0.05 CILEVEL=95.
Resources	Processor Time	00:00:00.38
	Elapsed Time	00:00:00.00

Hypothesis Test Summary

	Null Hypothesis	Test	Sig.[a,b]
1	The median of differences between In this section we present questions about science and questions about you. These will help us put learning in context. For each item below please rate your agreement with the item: - I can explain how a drug molecule and its target molecule interact using pictures, words or other representations. and In this section we present questions about science and questions about you. These will help us put learning in context. For each item below please rate your agreement with the item: - I can explain how a drug molecule and its target molecule interact using pictures, words or other representations. equals 0.	Related-Samples Wilcoxon Signed Rank Test	<.001

Hypothesis Test Summary

	Decision
1	Reject the null hypothesis.

a. The significance level is .050.

b. Asymptotic significance is displayed.

Related-Samples Wilcoxon Signed Rank Test

In this section we present questions about science and questions about you. These will help us put learning in context. For each item below please rate your agreement with the item: - I can explain how a drug molecule and its target molecule interact using pictures, words or other representations., In this section we present questions about science and questions about you. These will help us put learning in context. For each item below please rate your agreement with the item: - I can explain how a drug molecule and its target molecule interact using pictures, words or other representations.

Related-Samples Wilcoxon Signed Rank Test Summary

Total N	51
Test Statistic	89.500
Standard Error	70.197
Standardized Test Statistic	-4.281
Asymptotic Sig.(2-sided test)	<.001

Nonparametric Tests

Notes

Output Created		28-FEB-2024 15:13:02
Comments		
Input	Data	/Users/delmore/Documents/Research/Molecular Dynamics HS Project/Spring 2023 Data Analysis/Spring 2023 LSHS merged data for demo.sav
	Active Dataset	DataSet1
	Filter	<none>
	Weight	<none>
	Split File	<none>
	N of Rows in Working Data File	99
Syntax		NPTESTS /RELATED TEST (Perception_questions_8 Perception_questions_8_post) /MISSING SCOPE=ANALYSIS USERMISSING=EXCLUDE /CRITERIA ALPHA=0.05 CILEVEL=95.
Resources	Processor Time	00:00:00.37
	Elapsed Time	00:00:00.00

Hypothesis Test Summary

	Null Hypothesis	Test	Sig.[a,b]
1	The median of differences between In this section we present questions about science and questions about you. These will help us put learning in context. For each item below please rate your agreement with the item: - I get personal satisfaction when I solve a scientific problem by figuring it out myself. and In this section we present questions about science and questions about you. These will help us put learning in context. For each item below please rate your agreement with the item: - I get personal satisfaction when I solve a scientific problem by figuring it out myself. equals 0.	Related-Samples Wilcoxon Signed Rank Test	.014

Hypothesis Test Summary

	Decision
1	Reject the null hypothesis.

a. The significance level is .050.

b. Asymptotic significance is displayed.

Related-Samples Wilcoxon Signed Rank Test

In this section we present questions about science and questions about you. These wi ll help us put learning in context. For each item below please rate your agreement wit h the item: - I get personal satisfaction when I solve a scientific problem by figuring it out myself., In this section we present questions about science and questions abou t you. These will help us put learning in context. For each item below please rate your agreement with the item: - I get personal satisfaction when I solve a scientific probl em by figuring it out myself.

Related-Samples Wilcoxon Signed Rank Test Summary

Total N	51
Test Statistic	75.000
Standard Error	30.619
Standardized Test Statistic	-2.449
Asymptotic Sig.(2-sided test)	.014

Nonparametric Tests

Notes

Output Created		28-FEB-2024 15:13:31
Comments		
Input	Data	/Users/delmore/Documents/Research/Molecular Dynamics HS Project/Spring 2023 Data Analysis/Spring 2023 LSHS merged data for demo.sav
	Active Dataset	DataSet1
	Filter	<none>
	Weight	<none>
	Split File	<none>
	N of Rows in Working Data File	99
Syntax		NPTESTS /RELATED TEST (Perception_questions_9 Perception_questions_9_post) /MISSING SCOPE=ANALYSIS USERMISSING=EXCLUDE /CRITERIA ALPHA=0.05 CILEVEL=95.
Resources	Processor Time	00:00:00.41
	Elapsed Time	00:00:00.00

Hypothesis Test Summary

	Null Hypothesis	Test	Sig.[a,b]
1	The median of differences between In this section we present questions about science and questions about you. These will help us put learning in context. For each item below please rate your agreement with the item: - I consider myself a science person. and In this section we present questions about science and questions about you. These will help us put learning in context. For each item below please rate your agreement with the item: - I consider myself a science person. equals 0.	Related-Samples Wilcoxon Signed Rank Test	.591

Hypothesis Test Summary

	Decision
1	Retain the null hypothesis.

a. The significance level is .050.

b. Asymptotic significance is displayed.

Related-Samples Wilcoxon Signed Rank Test

In this section we present questions about science and questions about you. These will help us put learning in context. For each item below please rate your agreement with the item: - I consider myself a science person., In this section we present questions about science and questions about you. These will help us put learning in context. For each item below please rate your agreement with the item: - I consider myself a science person.

Related-Samples Wilcoxon Signed Rank Test Summary

Total N	51
Test Statistic	74.000
Standard Error	21.372
Standardized Test Statistic	-.538
Asymptotic Sig.(2-sided test)	.591

Crosstabs

Notes

Output Created		08-AUG-2023 12:57:14
Comments		
Input	Data	/Users/delmore/Documents/Research/Molecular Dynamics HS Project/Spring 2023 Data Analysis/Spring 2023 Written Interaction Analyses.sav
	Active Dataset	DataSet2
	Filter	<none>
	Weight	<none>
	Split File	<none>
	N of Rows in Working Data File	74
Missing Value Handling	Definition of Missing	User-defined missing values are treated as missing.
	Cases Used	Statistics for each table are based on all the cases with valid data in the specified range(s) for all variables in each table.
Syntax		CROSSTABS<br><br>/TABLES=feelforcesoruseforcesnotnecessarilyspecific bondingconflatingbondsandinteractions shapefit chargeselectrostatics refertointermolecularinteractions<br><br>referstomoleculesreactingormolecularchanges referstomoleculesbouncingcollidingtouching<br><br>referstomoleculesmoving referstobeingattractedtoorrepelledbyeachother drugfunction BY Prepost<br>/FORMAT=AVALUE TABLES<br>/STATISTICS=CHISQ<br>/CELLS=COUNT<br>/COUNT ROUND CELL.

Notes

Resources	Processor Time	00:00:00.04
	Elapsed Time	00:00:00.00
	Dimensions Requested	2
	Cells Available	524245

"bonding" (conflating bonds and interactions) * Pre/post

Crosstab

Count

		Pre/post		Total
		1	2	
"bonding" (conflating bonds and interactions)	0	30	32	62
	2	7	5	12
Total		37	37	74

Chi-Square Tests

	Value	df	Asymptotic Significance (2-sided)	Exact Sig. (2-sided)	Exact Sig. (1-sided)
Pearson Chi-Square	.398[a]	1	.528		
Continuity Correction[b]	.099	1	.752		
Likelihood Ratio	.399	1	.527		
Fisher's Exact Test				.754	.377
Linear-by-Linear Association	.392	1	.531		
N of Valid Cases	74				

a. 0 cells (.0%) have expected count less than 5. The minimum expected count is 6.00.

b. Computed only for a 2x2 table

shape/fit * Pre/post

Crosstab

Count

		Pre/post		Total
		1	2	
shape/fit	0	31	19	50
	2	6	18	24
Total		37	37	74

Chi-Square Tests

	Value	df	Asymptotic Significance (2-sided)	Exact Sig. (2-sided)	Exact Sig. (1-sided)
Pearson Chi-Square	8.880[a]	1	.003		
Continuity Correction[b]	7.462	1	.006		
Likelihood Ratio	9.187	1	.002		
Fisher's Exact Test				.006	.003
Linear-by-Linear Association	8.760	1	.003		
N of Valid Cases	74				

a. 0 cells (.0%) have expected count less than 5. The minimum expected count is 12.00.

b. Computed only for a 2x2 table

refers to molecules "reacting" or molecular changes * Pre/post

Crosstab

Count

		Pre/post		Total
		1	2	
refers to molecules "reacting" or molecular changes	0	33	37	70
	2	4	0	4
Total		37	37	74

Chi-Square Tests

	Value	df	Asymptotic Significance (2-sided)	Exact Sig. (2-sided)	Exact Sig. (1-sided)
Pearson Chi-Square	4.229[a]	1	.040		
Continuity Correction[b]	2.379	1	.123		
Likelihood Ratio	5.774	1	.016		
Fisher's Exact Test				.115	.057
Linear-by-Linear Association	4.171	1	.041		
N of Valid Cases	74				

a. 2 cells (50.0%) have expected count less than 5. The minimum expected count is 2.00.

b. Computed only for a 2x2 table

refers to molecules bouncing/colliding/touching * Pre/post

Crosstab

Count

		Pre/post 1	Pre/post 2	Total
refers to molecules bouncing/colliding/touching	0	34	35	69
	2	3	2	5
Total		37	37	74

Chi-Square Tests

	Value	df	Asymptotic Significance (2-sided)	Exact Sig. (2-sided)	Exact Sig. (1-sided)
Pearson Chi-Square	.214[a]	1	.643		
Continuity Correction[b]	.000	1	1.000		
Likelihood Ratio	.216	1	.642		
Fisher's Exact Test				1.000	.500
Linear-by-Linear Association	.212	1	.646		
N of Valid Cases	74				

a. 2 cells (50.0%) have expected count less than 5. The minimum expected count is 2.50.

b. Computed only for a 2x2 table

refers to being attracted to or repelled by each other * Pre/post

Crosstab

Count

		Pre/post 1	Pre/post 2	Total
refers to being attracted to or repelled by each other	0	32	33	65
	2	5	4	9
Total		37	37	74

Chi-Square Tests

	Value	df	Asymptotic Significance (2-sided)	Exact Sig. (2-sided)	Exact Sig. (1-sided)
Pearson Chi-Square	.126[a]	1	.722		
Continuity Correction[b]	.000	1	1.000		
Likelihood Ratio	.127	1	.722		
Fisher's Exact Test				1.000	.500
Linear-by-Linear Association	.125	1	.724		
N of Valid Cases	74				

a. 2 cells (50.0%) have expected count less than 5. The minimum expected count is 4.50.

b. Computed only for a 2x2 table

drug "function" * Pre/post

Crosstab

Count

		Pre/post		Total
		1	2	
drug "function"	0	26	23	49
	2	11	14	25
Total		37	37	74

Chi-Square Tests

	Value	df	Asymptotic Significance (2-sided)	Exact Sig. (2-sided)	Exact Sig. (1-sided)
Pearson Chi-Square	.544[a]	1	.461		
Continuity Correction[b]	.242	1	.623		
Likelihood Ratio	.545	1	.461		
Fisher's Exact Test				.624	.312
Linear-by-Linear Association	.536	1	.464		
N of Valid Cases	74				

a. 0 cells (.0%) have expected count less than 5. The minimum expected count is 12.50.

b. Computed only for a 2x2 table

Crosstabs

Notes

Output Created		08-AUG-2023 13:06:28
Comments		
Input	Data	/Users/delmore/Documents/Research/Molecular Dynamics HS Project/Spring 2023 Data Analysis/Spring 2023 Written Interaction Analyses.sav
	Active Dataset	DataSet2
	Filter	<none>
	Weight	<none>
	Split File	<none>
	N of Rows in Working Data File	74
Missing Value Handling	Definition of Missing	User-defined missing values are treated as missing.
	Cases Used	Statistics for each table are based on all the cases with valid data in the specified range(s) for all variables in each table.
Syntax		CROSSTABS<br><br>/TABLES=electrostat_IMF_recode BY Prepost<br>/FORMAT=AVALUE TABLES<br>/STATISTICS=CHISQ<br>/CELLS=COUNT<br>/COUNT ROUND CELL.
Resources	Processor Time	00:00:00.02
	Elapsed Time	00:00:00.00
	Dimensions Requested	2
	Cells Available	524245

electrostat_IMF_recode * Pre/post Crosstabulation

Count

		Pre/post		Total
		1	2	
electrostat_IMF_recode	.00	31	24	55
	2.00	6	13	19
Total		37	37	74

Chi-Square Tests

	Value	df	Asymptotic Significance (2-sided)	Exact Sig. (2-sided)	Exact Sig. (1-sided)
Pearson Chi-Square	3.470[a]	1	.062		
Continuity Correction[b]	2.549	1	.110		
Likelihood Ratio	3.534	1	.060		
Fisher's Exact Test				.109	.055
Linear-by-Linear Association	3.423	1	.064		
N of Valid Cases	74				

a. 0 cells (.0%) have expected count less than 5. The minimum expected count is 9.50.

b. Computed only for a 2x2 table

Crosstabs

Notes

Output Created		21-AUG-2023 16:52:53
Comments		
Input	Data	/Users/delmore/Documents/Research/Molecular Dynamics HS Project/Spring 2023 Data Analysis/Image analysis data.sav
	Active Dataset	DataSet2
	Filter	<none>
	Weight	<none>
	Split File	<none>
	N of Rows in Working Data File	78
Missing Value Handling	Definition of Missing	User-defined missing values are treated as missing.
	Cases Used	Statistics for each table are based on all the cases with valid data in the specified range(s) for all variables in each table.
Syntax		CROSSTABS<br><br>/TABLES=Morethanonedistinctobject Drawinghasshapenotjustacircle Indicationofshapecomplementarity<br><br>BallandstickLewisstructure Showingreasonablechargesorchargedistributionsoneachmoleculepartn<br><br>Explicitlyindicatesaninteractionbetweentwoobjectsvianoncovalents Denotesmotionorforce<br><br>Integratesdrugfunction BY Prepost<br>/FORMAT=AVALUE TABLES<br>/STATISTICS=CHISQ<br>/CELLS=COUNT<br>/COUNT ROUND CELL.
Resources	Processor Time	00:00:00.03
	Elapsed Time	00:00:00.00

Notes

	Dimensions Requested	2
	Cells Available	524245

```
[DataSet2] /Users/delmore/Documents/Research/Molecular Dynamics HS Project/Spring 2023 Da
ta Analysis/Image analysis data.sav
```

Case Processing Summary

	Cases					
	Valid		Missing		Total	
	N	Percent	N	Percent	N	Percent
More than one distinct object * Pre post	78	100.0%	0	0.0%	78	100.0%
Drawing has shape (not just a circle) * Pre post	78	100.0%	0	0.0%	78	100.0%
Indication of shape complementarity * Pre post	78	100.0%	0	0.0%	78	100.0%
Ball and stick/ Lewis structure * Pre post	78	100.0%	0	0.0%	78	100.0%
Showing reasonable charges or charge distributions on each molecule/partner * Pre post	78	100.0%	0	0.0%	78	100.0%
Explicitly indicates an interaction between two objects via non-covalent symbol * Pre post	78	100.0%	0	0.0%	78	100.0%
Denotes motion or force * Pre post	78	100.0%	0	0.0%	78	100.0%
Integrates drug function * Pre post	78	100.0%	0	0.0%	78	100.0%

More than one distinct object * Pre post

Crosstab

Count

		Pre post		
		1	2	Total
More than one distinct object	0	2	1	3
	2	37	38	75
Total		39	39	78

Chi-Square Tests

	Value	df	Asymptotic Significance (2-sided)	Exact Sig. (2-sided)	Exact Sig. (1-sided)
Pearson Chi-Square	.347[a]	1	.556		
Continuity Correction[b]	.000	1	1.000		
Likelihood Ratio	.353	1	.552		
Fisher's Exact Test				1.000	.500
Linear-by-Linear Association	.342	1	.559		
N of Valid Cases	78				

a. 2 cells (50.0%) have expected count less than 5. The minimum expected count is 1.50.

b. Computed only for a 2x2 table

Drawing has shape (not just a circle) * Pre post

Crosstab

Count

		Pre post		Total
		1	2	
Drawing has shape (not just a circle)	0	11	2	13
	2	28	37	65
Total		39	39	78

Chi-Square Tests

	Value	df	Asymptotic Significance (2-sided)	Exact Sig. (2-sided)	Exact Sig. (1-sided)
Pearson Chi-Square	7.477[a]	1	.006		
Continuity Correction[b]	5.908	1	.015		
Likelihood Ratio	8.110	1	.004		
Fisher's Exact Test				.013	.006
Linear-by-Linear Association	7.381	1	.007		
N of Valid Cases	78				

a. 0 cells (.0%) have expected count less than 5. The minimum expected count is 6.50.

b. Computed only for a 2x2 table

Indication of shape complementarity * Pre post

Crosstab

Count

		Pre post		Total
		1	2	
Indication of shape complementarity	0	20	6	26
	2	19	33	52
Total		39	39	78

Chi-Square Tests

	Value	df	Asymptotic Significance (2-sided)	Exact Sig. (2-sided)	Exact Sig. (1-sided)
Pearson Chi-Square	11.308[a]	1	<.001		
Continuity Correction[b]	9.750	1	.002		
Likelihood Ratio	11.769	1	<.001		
Fisher's Exact Test				.002	<.001
Linear-by-Linear Association	11.163	1	<.001		
N of Valid Cases	78				

a. 0 cells (.0%) have expected count less than 5. The minimum expected count is 13.00.

b. Computed only for a 2x2 table

Ball and stick/ Lewis structure * Pre post

Crosstab

Count

		Pre post		Total
		1	2	
Ball and stick/ Lewis structure	0	31	37	68
	2	8	2	10
Total		39	39	78

Chi-Square Tests

	Value	df	Asymptotic Significance (2-sided)	Exact Sig. (2-sided)	Exact Sig. (1-sided)
Pearson Chi-Square	4.129[a]	1	.042		
Continuity Correction[b]	2.868	1	.090		
Likelihood Ratio	4.385	1	.036		
Fisher's Exact Test				.087	.043
Linear-by-Linear Association	4.076	1	.043		
N of Valid Cases	78				

a. 0 cells (.0%) have expected count less than 5. The minimum expected count is 5.00.

b. Computed only for a 2x2 table

Showing reasonable charges or charge distributions on each molecule/partner * Pre post

Crosstab

Count

		Pre post		Total
		1	2	
Showing reasonable charges or charge distributions on each molecule/partner	0	36	33	69
	2	3	6	9
Total		39	39	78

Chi-Square Tests

	Value	df	Asymptotic Significance (2-sided)	Exact Sig. (2-sided)	Exact Sig. (1-sided)
Pearson Chi-Square	1.130[a]	1	.288		
Continuity Correction[b]	.502	1	.478		
Likelihood Ratio	1.150	1	.284		
Fisher's Exact Test				.481	.240
Linear-by-Linear Association	1.116	1	.291		
N of Valid Cases	78				

a. 2 cells (50.0%) have expected count less than 5. The minimum expected count is 4.50.

b. Computed only for a 2x2 table

Explicitly indicates an interaction between two objects via non-covalent symbol * Pre post

Crosstab

Count

		Pre post		Total
		1	2	
Explicitly indicates an interaction between two objects via non-covalent symbol	0	31	38	69
	2	8	1	9
Total		39	39	78

Chi-Square Tests

	Value	df	Asymptotic Significance (2-sided)	Exact Sig. (2-sided)	Exact Sig. (1-sided)
Pearson Chi-Square	6.155[a]	1	.013		
Continuity Correction[b]	4.522	1	.033		
Likelihood Ratio	6.909	1	.009		
Fisher's Exact Test				.029	.014
Linear-by-Linear Association	6.076	1	.014		
N of Valid Cases	78				

a. 2 cells (50.0%) have expected count less than 5. The minimum expected count is 4.50.

b. Computed only for a 2x2 table

Denotes motion or force * Pre post

Crosstab

Count

		Pre post		Total
		1	2	
Denotes motion or force	0	30	34	64
	2	9	5	14
Total		39	39	78

Chi-Square Tests

	Value	df	Asymptotic Significance (2-sided)	Exact Sig. (2-sided)	Exact Sig. (1-sided)
Pearson Chi-Square	1.393[a]	1	.238		
Continuity Correction[b]	.783	1	.376		
Likelihood Ratio	1.409	1	.235		
Fisher's Exact Test				.377	.188
Linear-by-Linear Association	1.375	1	.241		
N of Valid Cases	78				

a. 0 cells (.0%) have expected count less than 5. The minimum expected count is 7.00.

b. Computed only for a 2x2 table

Integrates drug function * Pre post

Crosstab

Count

		Pre post		Total
		1	2	
Integrates drug function	0	35	37	72
	2	4	2	6
Total		39	39	78

Chi-Square Tests

	Value	df	Asymptotic Significance (2-sided)	Exact Sig. (2-sided)	Exact Sig. (1-sided)
Pearson Chi-Square	.722[a]	1	.395		
Continuity Correction[b]	.181	1	.671		
Likelihood Ratio	.735	1	.391		
Fisher's Exact Test				.675	.337
Linear-by-Linear Association	.713	1	.398		
N of Valid Cases	78				

a. 2 cells (50.0%) have expected count less than 5. The minimum expected count is 3.00.

b. Computed only for a 2x2 table

Crosstabs

Notes

Output Created		08-AUG-2023 13:59:58
Comments		
Input	Data	/Users/delmore/Documents/Research/Molecular Dynamics HS Project/Spring 2023 Data Analysis/Interdisicplinary analysis.sav
	Active Dataset	DataSet3
	Filter	<none>
	Weight	<none>
	Split File	<none>
	N of Rows in Working Data File	72
Missing Value Handling	Definition of Missing	User-defined missing values are treated as missing.
	Cases Used	Statistics for each table are based on all the cases with valid data in the specified range(s) for all variables in each table.
Syntax		CROSSTABS<br><br>/TABLES=Mentionbiology Specificbiology Mentionphysics Specificphysics Mentionmath Specificmath<br>Mentionotherscience Specificotherscience Allthingsmadesofmolecules Drugrelatedexample<br>specific_any_recode BY Prepost<br>/FORMAT=AVALUE TABLES<br>/STATISTICS=CHISQ<br>/CELLS=COUNT<br>/COUNT ROUND CELL.
Resources	Processor Time	00:00:00.05
	Elapsed Time	00:00:00.00
	Dimensions Requested	2
	Cells Available	524245

```
[DataSet3] /Users/delmore/Documents/Research/Molecular Dynamics HS Project/Spring 2023 Da
ta Analysis/Interdisicplinary analysis.sav
```

Mention biology * Pre/post

Crosstab

Count

		Pre/post 1	Pre/post 2	Total
Mention biology	0	17	20	37
	2	19	16	35
Total		36	36	72

Chi-Square Tests

	Value	df	Asymptotic Significance (2-sided)	Exact Sig. (2-sided)	Exact Sig. (1-sided)
Pearson Chi-Square	.500[a]	1	.479		
Continuity Correction[b]	.222	1	.637		
Likelihood Ratio	.501	1	.479		
Fisher's Exact Test				.638	.319
Linear-by-Linear Association	.493	1	.482		
N of Valid Cases	72				

a. 0 cells (.0%) have expected count less than 5. The minimum expected count is 17.50.

b. Computed only for a 2x2 table

Specific biology * Pre/post

Crosstab

Count

		Pre/post 1	Pre/post 2	Total
Specific biology	0	27	25	52
	2	9	11	20
Total		36	36	72

Chi-Square Tests

	Value	df	Asymptotic Significance (2-sided)	Exact Sig. (2-sided)	Exact Sig. (1-sided)
Pearson Chi-Square	.277[a]	1	.599		
Continuity Correction[b]	.069	1	.792		
Likelihood Ratio	.277	1	.598		
Fisher's Exact Test				.793	.396
Linear-by-Linear Association	.273	1	.601		
N of Valid Cases	72				

a. 0 cells (.0%) have expected count less than 5. The minimum expected count is 10.00.

b. Computed only for a 2x2 table

Mention other science * Pre/post

Crosstab

Count

		Pre/post		Total
		1	2	
Mention other science	0	34	36	70
	2	2	0	2
Total		36	36	72

Chi-Square Tests

	Value	df	Asymptotic Significance (2-sided)	Exact Sig. (2-sided)	Exact Sig. (1-sided)
Pearson Chi-Square	2.057[a]	1	.151		
Continuity Correction[b]	.514	1	.473		
Likelihood Ratio	2.830	1	.093		
Fisher's Exact Test				.493	.246
Linear-by-Linear Association	2.029	1	.154		
N of Valid Cases	72				

a. 2 cells (50.0%) have expected count less than 5. The minimum expected count is 1.00.

b. Computed only for a 2x2 table

Specific other science * Pre/post

Crosstab

Count

		Pre/post 1	Pre/post 2	Total
Specific other science	0	35	36	71
	2	1	0	1
Total		36	36	72

Chi-Square Tests

	Value	df	Asymptotic Significance (2-sided)	Exact Sig. (2-sided)	Exact Sig. (1-sided)
Pearson Chi-Square	1.014[a]	1	.314		
Continuity Correction[b]	.000	1	1.000		
Likelihood Ratio	1.400	1	.237		
Fisher's Exact Test				1.000	.500
Linear-by-Linear Association	1.000	1	.317		
N of Valid Cases	72				

a. 2 cells (50.0%) have expected count less than 5. The minimum expected count is .50.

b. Computed only for a 2x2 table

All things mades of molecules * Pre/post

Crosstab

Count

		Pre/post 1	Pre/post 2	Total
All things mades of molecules	0	31	32	63
	2	5	4	9
Total		36	36	72

Chi-Square Tests

	Value	df	Asymptotic Significance (2-sided)	Exact Sig. (2-sided)	Exact Sig. (1-sided)
Pearson Chi-Square	.127[a]	1	.722		
Continuity Correction[b]	.000	1	1.000		
Likelihood Ratio	.127	1	.721		
Fisher's Exact Test				1.000	.500
Linear-by-Linear Association	.125	1	.723		
N of Valid Cases	72				

a. 2 cells (50.0%) have expected count less than 5. The minimum expected count is 4.50.

b. Computed only for a 2x2 table

Drug-related example * Pre/post

Crosstab

Count

		Pre/post		
		1	2	Total
Drug-related example	0	35	28	63
	2	1	8	9
Total		36	36	72

Chi-Square Tests

	Value	df	Asymptotic Significance (2-sided)	Exact Sig. (2-sided)	Exact Sig. (1-sided)
Pearson Chi-Square	6.222[a]	1	.013		
Continuity Correction[b]	4.571	1	.033		
Likelihood Ratio	6.977	1	.008		
Fisher's Exact Test				.028	.014
Linear-by-Linear Association	6.136	1	.013		
N of Valid Cases	72				

a. 2 cells (50.0%) have expected count less than 5. The minimum expected count is 4.50.

b. Computed only for a 2x2 table

Crosstabs

Notes

Output Created		08-AUG-2023 14:13:28
Comments		
Input	Data	/Users/delmore/Documents/Research/Molecular Dynamics HS Project/Spring 2023 Data Analysis/Interdisicplinary analysis.sav
	Active Dataset	DataSet3
	Filter	<none>
	Weight	<none>
	Split File	<none>
	N of Rows in Working Data File	72
Missing Value Handling	Definition of Missing	User-defined missing values are treated as missing.
	Cases Used	Statistics for each table are based on all the cases with valid data in the specified range(s) for all variables in each table.
Syntax		CROSSTABS /TABLES=math_phys_mention_recode math_phys_specific_recode BY Prepost /FORMAT=AVALUE TABLES /STATISTICS=CHISQ /CELLS=COUNT /COUNT ROUND CELL.
Resources	Processor Time	00:00:00.02
	Elapsed Time	00:00:00.00
	Dimensions Requested	2
	Cells Available	524245

Recoded math/phys mention * Pre/post

Crosstab

Count

		Pre/post		Total
		1	2	
Recoded math/phys mention	.00	30	21	51
	2.00	6	15	21
Total		36	36	72

Chi-Square Tests

	Value	df	Asymptotic Significance (2-sided)	Exact Sig. (2-sided)	Exact Sig. (1-sided)
Pearson Chi-Square	5.445[a]	1	.020		
Continuity Correction[b]	4.303	1	.038		
Likelihood Ratio	5.581	1	.018		
Fisher's Exact Test				.037	.018
Linear-by-Linear Association	5.370	1	.020		
N of Valid Cases	72				

a. 0 cells (.0%) have expected count less than 5. The minimum expected count is 10.50.

b. Computed only for a 2x2 table

Recoded math/phys specific * Pre/post

Crosstab

Count

		Pre/post		
		1	2	Total
Recoded math/phys specific	.00	31	27	58
	2.00	5	9	14
Total		36	36	72

Chi-Square Tests

	Value	df	Asymptotic Significance (2-sided)	Exact Sig. (2-sided)	Exact Sig. (1-sided)
Pearson Chi-Square	1.419[a]	1	.234		
Continuity Correction[b]	.798	1	.372		
Likelihood Ratio	1.435	1	.231		
Fisher's Exact Test				.372	.186
Linear-by-Linear Association	1.399	1	.237		
N of Valid Cases	72				

a. 0 cells (.0%) have expected count less than 5. The minimum expected count is 7.00.

b. Computed only for a 2x2 table
