# Supplementary material for: EMMAs: Implementation and Assessment of a Suite of Cross-Disciplinary, Case-Based High School Activities to Explore Three-Dimensional Molecular Structure, Noncovalent Interactions, and Molecular Dynamics
Source: J Chem Educ. 2024 May 10;101(6):2436–47. doi: 10.1021/acs.jchemed.4c00036 (PMC11171454; doi:10.1021/acs.jchemed.4c00036)
Supplement: Supplementary file 1 — ed4c00036_si_001.zip [file ed4c00036_si_001.zip › Kotsalidis_supporting_info_revisions/04 - Exploring Molecular Interactions Using VMD-Ponatinib_Abl Kinase Chem 2.docx]

**Exploring Molecular Interactions Using VMD-Ponatinib/Abl Kinase**

| **READ THIS:** Today you are going to explore a **complex**, or a system made from more than one molecule interacting (or binding) with each other. The complex you will be studying involves two molecules – the drug molecule **ponatinib**, which you studied in an earlier activity, and the protein molecule **Abl kinase**, which you also studied in an earlier activity and is the structurally important part of ponatinib’s **target**, the Bcr-Abl kinase. By studying this complex, you will begin to understand how **ponatinib** interacts with this target to treat chronic myeloid leukemia. |
| --- |

1. **Loading the Drug/Protein Complex into VMD**

Use what you have learned from the previous activities to load the ponatinib_ablkinase file into VMD.
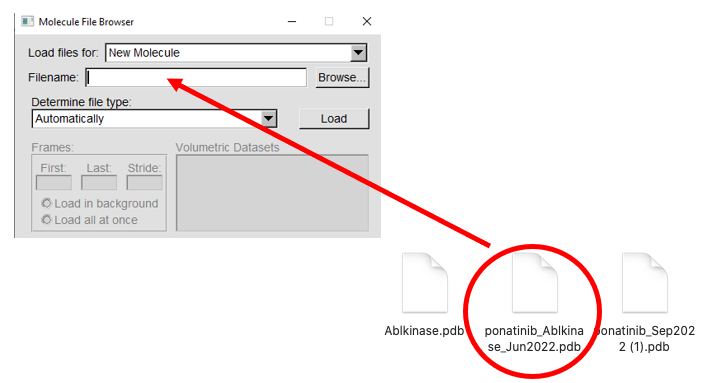


You will see something like this after the molecule is loaded:
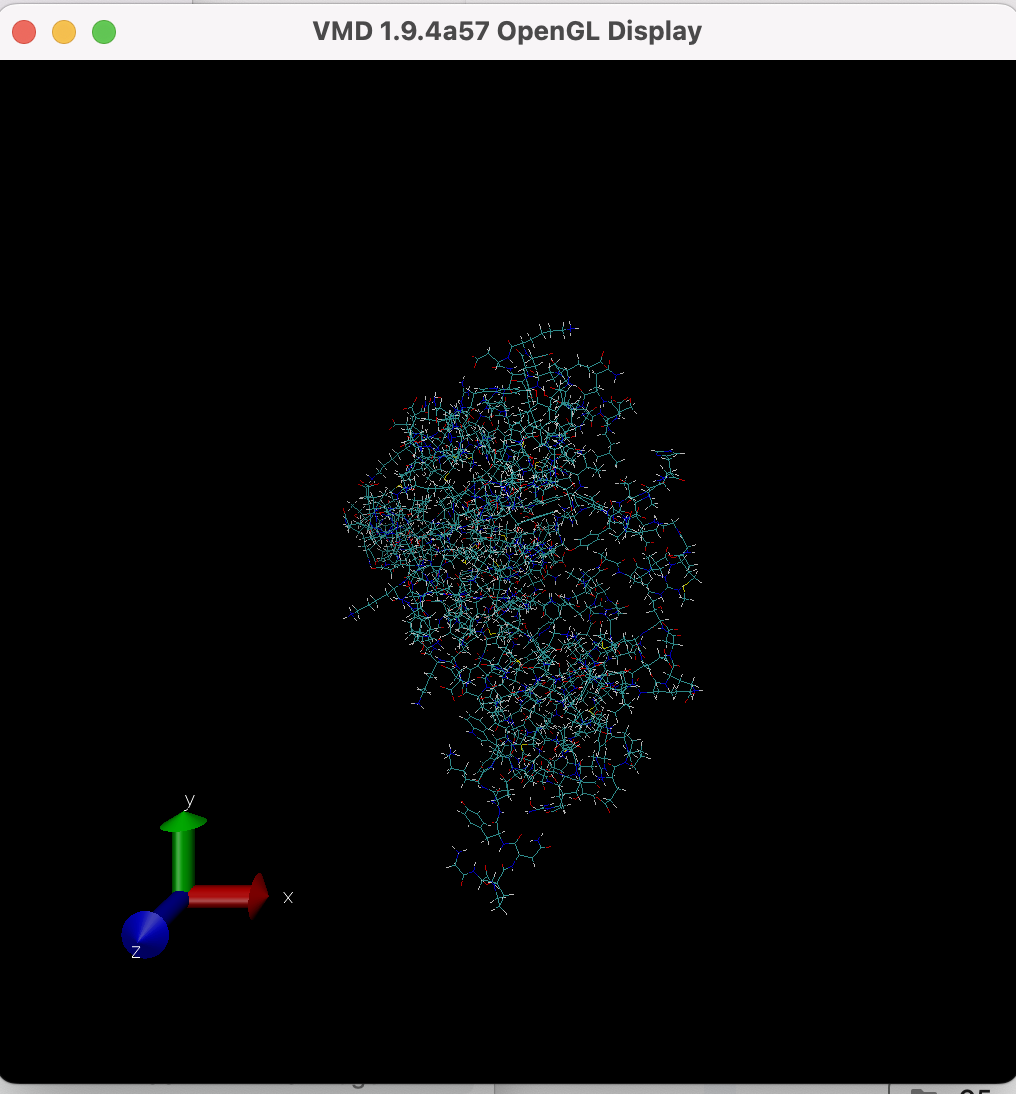


Please refer to the list of useful [VMD commands](https://docs.google.com/document/d/1hrvWnPcHNHJkrqHxHY8J5Jtwa4spaki0rfVGo3ZDZsE/edit?usp=drive_link) to remind yourself how to rotate, zoom, translate, and center.

1. **Visualizing Each Molecule Differently Through Creating Multiple Representations**

There are two molecules in this complex, but they are currently very hard to see distinctly. In a complex, each molecule is usually assigned to a different “chain”. In this particular complex, the protein is assigned to “chain A” and the drug is “chain D”.
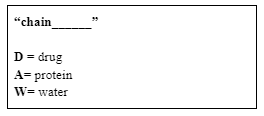


You can use **multiple representations** to more easily visualize each molecule separately:

1. In the **VMD Main window** select Graphics → Representations. A new window will pop up, the **Graphical Representations** window.
2. In the “Selected Atoms” box, replace the word “all” with “chain A” and press enter. You may not notice a difference in what you see in the OpenGL Display, but the drug molecule will no longer be shown.
3. Select the following:
   1. **Coloring Method: ColorID** (you will see the number “0” to the right, which yields a dark blue). You can change this number to be a different number if you’d like to use a different color. For now, keep it blue.
   2. **Drawing method: VDW**
4. In a few words describe what you see. **(2 points)**

| **Delete this text and type your answer here.** |
| --- |

1. Now, click on the “Create Rep” button on the “Graphical Representations” Window:


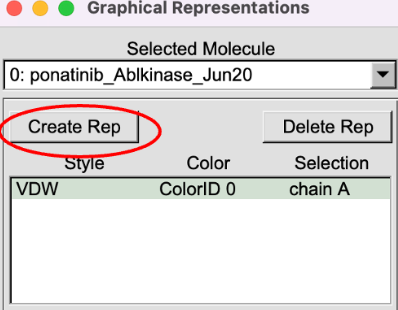


1. You will now see two identical lines of text show up in the whitespace below the button.


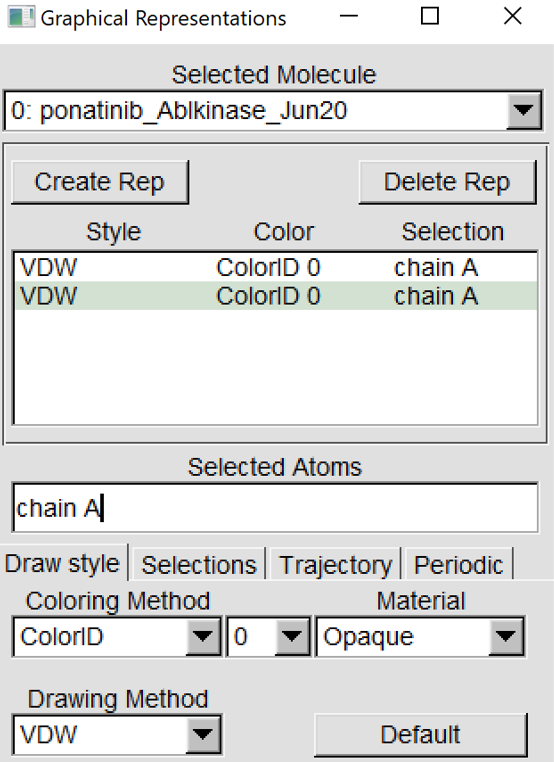


1. In the “Selected Atoms” space, delete “chain A” and type in “Chain D” and press enter.

Change the “ColorID” of this representation to “1” (red). In a sentence or two, describe what you see. **(2 points)**

| **Delete this text and type your answer here.** |
| --- |

1. **IMPORTANT:** You can move between each selection (in this case, the drug and target) by clicking on one or the other selection in the “Graphical Representations” window.


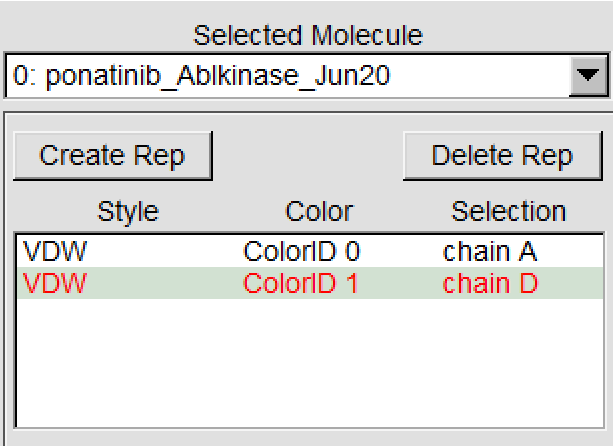


1. Try double clicking on the representation until the words turn to red.
2. In a few words describe what happens to the drug and target when you click on the representation until it turns red. **(1 point)**

| **Delete this text and type your answer here.** |
| --- |

The selected representation is highlighted in green. Once you’ve selected one or the other representation, you can customize how you represent that selection by changing its Coloring Method and Drawing Method.

1. Use your skills in VMD to represent the system as follows:
2. First, make the drug (chain D) have a licorice drawing representation and colored by “Name”


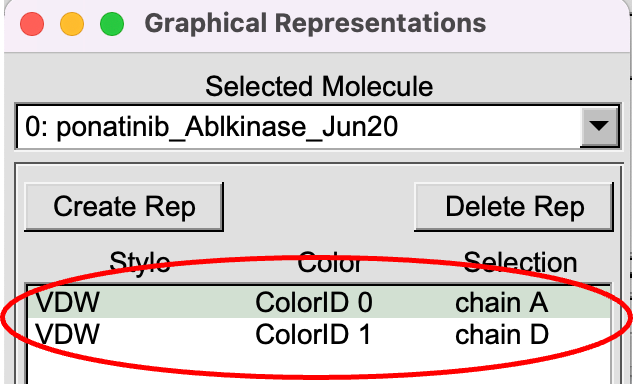


1. Next, make the protein (chain A) have a “Lines” representation and remain colored blue. Note that when you choose “Lines”, you can change the thickness of the lines using the buttons at the bottom of the Graphical Representation Window to make them clearer (we prefer a thickness of “2”)

Rotate your system so you can clearly see the drug within the protein binding pocket (a “pocket” of space within the 3D protein structure that a small molecule can physically fit into)

13. **Have your teacher check your representation of the drug and target molecule. (2 points)**

14. Now try to represent your drug and protein in whatever different representations you choose for each. For the drug, we recommend you choose a representation from among lines, licorice, CPK, or VDW. For the protein we recommend you choose a representation from among lines, licorice, CPK, VDW, cartoon, or ribbons.

15. In a sentence or two, explain why you think it might be useful to represent different molecules in different ways when looking at a complex. **(2 points)**

| **Delete this text and type your answer here.** |
| --- |

1. **Identifying Hydrogen Bonds Between Drug and Protein Target**

As you can see and explore using VMD, the drug has a shape that enables it to fit within the ATP-binding pocket of this kinase. But in addition to having a shape that is **complementary**, or just the right fit, the drug also makes hydrogen bonds with the protein. To see the hydrogen bonds, it helps to focus only on parts of the protein that are very close to the drug. We can use VMD to select only those amino acid residues on the protein that are very close to the drug in this complex:

1. First, change the representation of the drug (chain D) to use the Drawing Method “Licorice” and Coloring Method “Name” if those are currently not the selected representations.

2. Next, click on the “chain A” selection and replace “chain A” in the “Selected Atoms” window with the following:

**“same resid as within 4 of chain D”**

And press enter. Don’t forget to use the [Helpful VMD commands](https://docs.google.com/document/d/1hrvWnPcHNHJkrqHxHY8J5Jtwa4spaki0rfVGo3ZDZsE/edit?usp=drive_link) sheet.

What does this do? **This selection tells VMD to show only the residues (amino acids) of the protein that have at least one atom that is within a distance of 4 Angstroms (where one Angstrom is 10^-10^ m) from at least one atom on the drug. In other words, it shows parts of the protein that are close to the drug in 3D space.** Because hydrogen bonds occur between molecules only when the molecules are very close to each other, this is a good way to remove irrelevant parts of the protein when trying to find hydrogen bonds.

3. Use Drawing Method “Lines” and set the Thickness to a value that you like. Use the Coloring Method “Name” so you can identify different types of atoms. Center your view on the drug by pressing “c” and then clicking on any atom on the drug. Then translate and rotate as necessary to get your system into the middle of the Display window. You may see something similar to this:


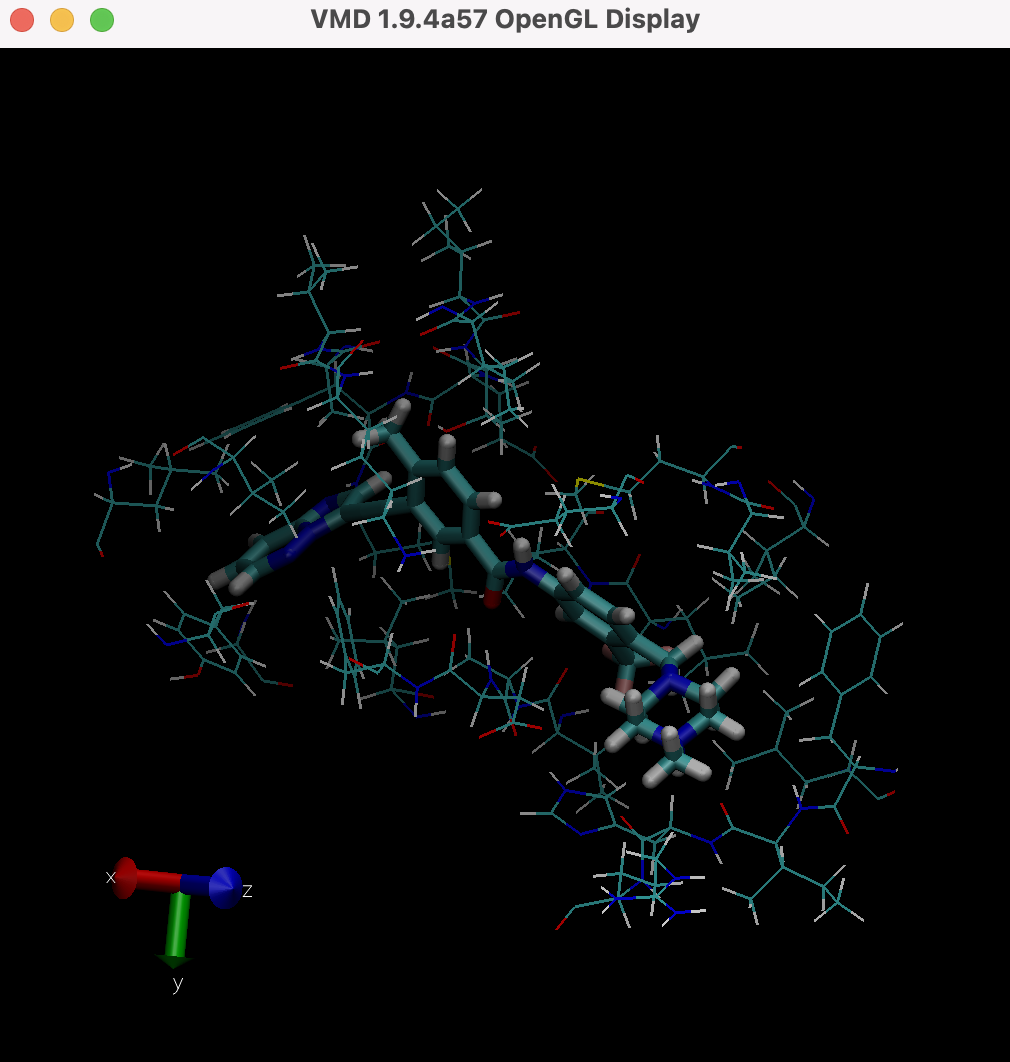


| **Color** | **Atom Type** |
| --- | --- |
| Turquoise | Carbon |
| White | Hydrogen |
| Red | Oxygen |
| Blue | Nitrogen |
| Pink | Fluorine |
| Yellow | Sulfur |

4. This year, you have learned about **hydrogen bonds, which are noncovalent (intermolecular) interactions that occur between an O, N, or F atom that has a partial negative charge and an H atom that is bonded to an O, N, or F atom and therefore has a partial positive charge. Hydrogen bonds typically have a length of between 2-3 Angstroms** – they are longer than a covalent bond, but can be strong enough to hold two different molecules together.

**(1 Angstrom = 10^-10^ m)**

**CHALLENGE: (10 points)**  Use your VMD skills (rotating, zooming, translating, centering, finding distance, and observing) to see if you can identify an atom on the drug (chain D) that is involved in a hydrogen bond with an atom on the target protein (chain A). These bonds are not shown in VMD by default. So you instead will use your knowledge of chemistry to find them. Once you believe you have found a hydrogen bond, find the length of the bond by pressing “2” and then clicking on the two atoms.

How many hydrogen bonds can you find between the drug and the target? Hint: there are four total! Record the length of each hydrogen bond in the box below.

⭐You can hide labels and bonds by pressing “2” and clicking on the two atoms again. As another way to delete the labels and dashed white lines from your molecule, you can go to the VMD Main window and click on Graphics -> Labels. Select the atoms or bonds that you want to delete.⭐

| **Hydrogen Bond** | **Distance in Angstroms ( 1 Angstrom = 10^-10^ m)** |
| --- | --- |
| **#1** | **Delete this text and type your answer here.** |
| **#2** | **BONUS** |
| **#3** | **BONUS** |
| **#4** | **BONUS** |


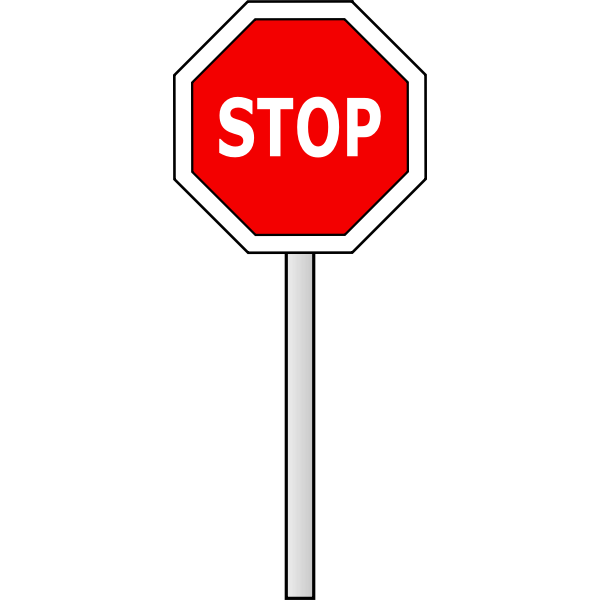
**Have your teacher check your hydrogen bond distances and representation.**
