# Supplementary material for: EMMAs: Implementation and Assessment of a Suite of Cross-Disciplinary, Case-Based High School Activities to Explore Three-Dimensional Molecular Structure, Noncovalent Interactions, and Molecular Dynamics
Source: J Chem Educ. 2024 May 10;101(6):2436–47. doi: 10.1021/acs.jchemed.4c00036 (PMC11171454; doi:10.1021/acs.jchemed.4c00036)
Supplement: Supplementary file 1 — ed4c00036_si_001.zip [file ed4c00036_si_001.zip › Kotsalidis_supporting_info_revisions/00 - SUMMARY of EMMAs.docx]

***EMMAs: Exploring Molecular Modeling through case-based Activities***

(Note – a similar document originally appeared in the New England Association of Chemistry Teachers’ Summer conference 2023 (Engaging Chemistry: Hands on, Minds on), Aug. 1-2 Fitchburg State Univ.)

**If you have questions or want an updated version of activities with live links for easy navigation, please reach out to**

[mradhakr@wellesley.edu](https://mail.google.com/mail/?view=cm&fs=1&to=mradhakr%40wellesley.edu&authuser=4)

Linked materials as of 2023 can also be found at:

<https://docs.google.com/document/d/19pdo-LRTVRrNbSOWjJ6YgE3m6uWcx-thpUSQ5Ww2mes/edit>

******The following files are useful resources throughout all VMD-related activities:**

A-HELPFUL VMD COMMANDS

B-AMINO ACIDS IN PROTEINS HANDOUT

******The following files provide learning goals for activities and relate them to certain standards:**

C – EMMAs Learning Outcomes for Activities

D – EMMAs State_NGSS_AP College Board Items Addressed

| FILENAME/ACTIVITY | DESCRIPTION |
| --- | --- |
| 00 - Installing Visual Molecular Dynamics (VMD) Software  00 - Folder of VMD Files | These documents include Instructions on downloading VMD onto your computer and a collection of files used in the project.  We highly recommend having your tech folks install VMD and the files onto school machines before students work on the project. |
| [01 - Chronic Myeloid Leukemia Case Study](https://docs.google.com/document/d/1leFG95tDDq2uBjPrBd2GhAeDcdPjMoCVlmCMNOg1wKE/edit?usp=drive_link) | Students meet Sandy: a woman diagnosed with Chronic Myeloid Leukemia. There are two EdPuzzles in the brief case study. We assigned this as homework. |
| 02 - VMD Ponatinib & Abl Kinase Chem 1  02 - VMD Ponatinib & Abl Kinase Chem 2 | This activity is a two-part lesson where students learn the basics of VMD to manipulate and analyze a TKI drug first, and then its target protein second. It took students about 30 minutes to finish Part 1, the drug exploration. An atomic structure/intra-bonding challenge is at the end of part 1. The second part of the lesson allows students to use VMD skills to analyze the drug target…Abl-1 Kinase. The second part of the activity takes students about 25 minutes, so some groups will finish in one class period, while others will need to complete it during the next class.  Students worked in teams of two, occasionally three, where one was in charge of VMD, and the other worked through the tutorial or activity questions. In the rare case of a group of 3, the student on VMD is in the middle, and two students work through the google doc. Students access materials on google classroom, and each student receives a copy of the assignment. Students only submit one doc per group for grading. Note that all the VMD files in the folder of VMD files (00 - Folder of VMD Files) were pre-loaded onto each classroom computer into a folder named “VMD”; this way students could just drag files from this folder into the VMD dialog box to load them, as per the instructions in the activity. |
| 03 - CML Stories Investigation | Students choose between four athletes diagnosed with CML and read their personal stories. Students answer questions in a google doc. We assigned this for homework. |
| 04 - VMD Ponatinib & Abl Kinase Interactions Chem 1  04 - VMD Ponatinib & Abl Kinase Interactions Chem 2 | Students use their VMD skills to explore the interactions between the drug and protein. There is a brief hydrogen bonding challenge at the end of the activity. |
| 05 - Secret Code Activity Form  05 - Secret Code Activity Clues (Task Cards)  [05 - Secret Code Website](https://sites.google.com/wellesley.edu/cracking-the-cml-code/home) | Students apply everything they’ve learned about VMD, Chemical Bonding, and interparticle forces to solve a creative and ambitious word challenge. (Will take between 35 – 60 min if students are split into groups where each group does ~3-4 clues, depending on student ability)  The activity provides students clues often based on interactions between the drug and specific amino acids of the protein. Some clues pertain to noncovalent interactions between amino acids within the protein. Depending on how much students remember about amino acids from first year biology, they may need a refresher before engaging with the challenge. In our implementation, students worked through guided notes and a brief practice before completing the Secret Code.  We scaffolded for different levels through *hint* cards. For the Chem 2 classes, we walked through the clues as a class. We projected VMD on the front wall, and everyone walked through the steps together. For Chem 2, we completed only the first word.  The website provides a virtual version of the activity! |
| 06 - Molecular Dynamics EdPuzzle 1 Video  06 - Molecular Dynamics EdPuzzle 2 Video  06 – Molecular Dynamics EdPuzzle Questions | Students explore dynamic systems through a few videos that Nena created on Molecular Dynamics. These EdPuzzles can be homework, but we chose to complete these as a whole class since most students had never been exposed to the content before, and an appreciation for the importance of exploring chemical systems with Molecular Dynamics is a goal of the project. |
| 07 - Investigations of MD Simulations Activity  07 - Investigation of MD Simulations Post-Lab | Activities under development at the time of manuscript submission for students to explore Molecular Dynamics and mechanisms of drug resistance within the Abl-Kinase system more deeply (see Discussion Section) |

**If you have questions or want an updated version of activities with live links for easy navigation, please reach out to**

[mradhakr@wellesley.edu](https://mail.google.com/mail/?view=cm&fs=1&to=mradhakr%40wellesley.edu&authuser=4)
