# Supplementary material for: EMMAs: Implementation and Assessment of a Suite of Cross-Disciplinary, Case-Based High School Activities to Explore Three-Dimensional Molecular Structure, Noncovalent Interactions, and Molecular Dynamics
Source: J Chem Educ. 2024 May 10;101(6):2436–47. doi: 10.1021/acs.jchemed.4c00036 (PMC11171454; doi:10.1021/acs.jchemed.4c00036)
Supplement: Supplementary file 1 — ed4c00036_si_001.zip [file ed4c00036_si_001.zip › Kotsalidis_supporting_info_revisions/I - Kotsalidis_supp_info_2_survey_instruments..docx]

**SUPPORTING INFORMATION PART II**

**for**

**EMMAs: A Suite of Cross-Disciplinary, Cased-Based High School Activities to Explore Three-Dimensional Molecular Structure, Noncovalent Interactions, and Molecular Dynamics**

**Supporting Information Related to the Survey Instruments:**

Pre-survey used for Kotsalidis et al.

Below are a series of questions related to our research project considering how computational molecular modeling impacts student learning. We expect that you will be able to complete these questions in 5-10 minutes. 

You can skip any questions you do not wish to answer other than entering your codename. Your teacher will not know your answers to these questions or whether you are participating in this study.

What is your race/ethnicity? Please check all that apply.

- Alaskan Native (1)
- American Indian (2)
- Asian American (3)
- Black or African American (4)
- Filipino (5)
- Hawaiian (6)
- Hispanic/Latino (7)
- Pacific Islander (8)
- White (9)
- Other (10) __________________________________________________
- Prefer not to answer (11)

What is your gender?

- Female (1)
- Male (2)
- Non-binary (3)
- Other (4) __________________________________________________
- Prefer not to answer (5)

What class/grade are you?

- I am a high school first-year/freshman (9th grade) (1)
- I am a high school sophomore (10th grade) (2)
- I am a high school junior (11th grade) (3)
- I am a high school senior (12th grade) (4)
- Other (5) __________________________________________________
- Prefer not to answer (6)

What chemistry course are you currently taking?

- Chem 1 (1)
- Chem 2 (2)

Please indicate all high school science courses you have already taken or are currently taking

- Biology (1)
- Biotechnology (2)
- Environmental science (4)
- Forensics (5)
- Anatomy and Physiology (6)
- Aquatic Biology (7)
- Physics (8)
- Other (9) __________________________________________________

In this section we present questions about science and questions about you. These will help us put learning in context. For each item below please rate your agreement with the item:

|  | Strongly agree (1) | Agree (2) | Neither agree nor disagree (3) | Disagree (4) | Strongly disagree (5) |
| --- | --- | --- | --- | --- | --- |
| I understand different ways to visualize a molecule. (1) |  |  |  |  |  |
| Molecules interact with each other. (2) |  |  |  |  |  |
| Molecules are not always moving. (3) |  |  |  |  |  |
| I can picture molecules interacting in my mind. (4) |  |  |  |  |  |
| The shapes of molecules do not impact how they interact with one another. (5) |  |  |  |  |  |
| How well two molecules interact with each other can be influenced by the locations of their charges. (6) |  |  |  |  |  |

| I can explain how a drug molecule and its target molecule interact using pictures, words or other representations. (7) |  |  |  |  |  |
| --- | --- | --- | --- | --- | --- |
| I get personal satisfaction when I solve a scientific problem by figuring it out myself. (8) |  |  |  |  |  |
| I consider myself a science person. (9) |  |  |  |  |  |

Describe one way in which understanding how two molecules interact relates to topics in math or other sciences, such as biology or physics.

________________________________________________________________

In no more than three sentences describe how you think a drug molecule and a target molecule can interact with one another. Note that there is not a specific "correct" or "right" answer to this question.

________________________________________________________________

Draw a sketch or picture in the box below of how you think a drug molecule and a target molecule can interact with one another. Note that there is not a specific "correct" or "right" answer to this question.

Please let us know if you have any additional comments related to this survey or the questions we have asked in the box below. When you click the arrow your survey results will be submitted.

________________________________________________________________

Post-survey used for Kotsalidis et al.

Below are a series of questions related to our research project considering how computational molecular modeling impacts student learning. We expect that you will be able to complete these questions in 5-10 minutes. 


You can skip any questions you do not wish to answer other than entering your codename. Your teacher will not know your answers to these questions or whether you are participating in this study.

In this section we present questions about science and questions about you. These will help us put learning in context. For each item below please rate your agreement with the item:

|  | Strongly agree (1) | Agree (2) | Neither agree nor disagree (3) | Disagree (4) | Strongly disagree (5) |
| --- | --- | --- | --- | --- | --- |
| I understand different ways to visualize a molecule. (1) |  |  |  |  |  |
| Molecules interact with each other. (2) |  |  |  |  |  |
| Molecules are not always moving. (3) |  |  |  |  |  |
| I can picture molecules interacting in my mind. (4) |  |  |  |  |  |
| The shapes of molecules do not impact how they interact with one another. (5) |  |  |  |  |  |
| How well two molecules interact with each other can be influenced by the locations of their charges. (6) |  |  |  |  |  |
| I can explain how a drug molecule and its target molecule interact using pictures, words or other representations. (7) |  |  |  |  |  |
| I get personal satisfaction when I solve a scientific problem by figuring it out myself. (8) |  |  |  |  |  |
| I consider myself a science person. (9) |  |  |  |  |  |

Describe one way in which understanding how two molecules interact relates to topics in math or other sciences, such as biology or physics.

________________________________________________________________

In no more than three sentences describe how you think a drug molecule and a target molecule can interact with one another. Note that there is not a specific "correct" or "right" answer to this question.

________________________________________________________________

Draw a sketch or picture in the box below of how you think a drug molecule and a target molecule can interact with one another. Note that there is not a specific "correct" or "right" answer to this question.

What was one thing you learned from the molecular modeling activities?

________________________________________________________________

What was one thing you liked about the molecular modeling activities?

________________________________________________________________

What was one thing you would change to improve the molecular modeling activities?

________________________________________________________________

Evaluate your overall sense of satisfaction with the molecular modeling activities by choosing one statement below.

- I am very satisfied by the experience (1)
- I am satisfied by the experience (2)
- I feel neural about the experience (3)
- I am dissatisfied by the experience (4)
- I am very dissatisfied by the experience (5)

Would you be interested in completing other molecular modeling activities in the future?

- I would be very interested in participating in and completing other molecular modeling activities (1)
- I would be somewhat interested in participating in and completing other molecular modeling activities (2)
- I would not be interested in participating in and completing other molecular modeling activities (3)

Please let us know if you have any additional comments related to this survey or the questions we have asked in the box below. When you click the arrow your survey results will be submitted.

________________________________________________________________
