# Supplementary material for: EMMAs: Implementation and Assessment of a Suite of Cross-Disciplinary, Case-Based High School Activities to Explore Three-Dimensional Molecular Structure, Noncovalent Interactions, and Molecular Dynamics
Source: J Chem Educ. 2024 May 10;101(6):2436–47. doi: 10.1021/acs.jchemed.4c00036 (PMC11171454; doi:10.1021/acs.jchemed.4c00036)
Supplement: Supplementary file 1 — ed4c00036_si_001.zip [file ed4c00036_si_001.zip › Kotsalidis_supporting_info_revisions/05 - Secret Code Task Cards.docx]

| **Word #1 / Clue #1**  ***“I have a positively charged R-group with three nitrogen atoms that interacts with the negatively charged R-group of amino acid residue 81.”*** | **Word #1 / Clue #1**  ***“I have a positively charged R-group with three nitrogen atoms that interacts with the negatively charged R-group of amino acid residue 81.”*** |
| --- | --- |
| **Word #1 / Clue #1**  ***“I have a positively charged R-group with three nitrogen atoms that interacts with the negatively charged R-group of amino acid residue 81.”*** | **Word #1 / Clue #1**  ***“I have a positively charged R-group with three nitrogen atoms that interacts with the negatively charged R-group of amino acid residue 81.”*** |

| **Word #1 / Clue #2**  ***“My backbone oxygen atom forms a nice hydrogen bond (length 1.88 Angstroms) with a hydrogen on residue 96”*** | **Word #1 / Clue #2**  ***“My backbone oxygen atom forms a nice hydrogen bond (length 1.88 Angstroms) with a hydrogen on residue 96”*** |
| --- | --- |
| **Word #1 / Clue #2**  ***“My backbone oxygen atom forms a nice hydrogen bond (length 1.88 Angstroms) with a hydrogen on residue 96”*** | **Word #1 / Clue #2**  ***“My backbone oxygen atom forms a nice hydrogen bond (length 1.88 Angstroms) with a hydrogen on residue 96”*** |

| **Word #1 / Clue #3**  ***“I am NOT a hydrophobic amino acid.. I am the ONLY non-hydrophobic amino acid that is within 3 Angstroms of the three fluorine atoms (pink) on the drug.*** | **Word #1 / Clue #3**  ***“I am NOT a hydrophobic amino acid. I am the ONLY non-hydrophobic amino acid that is within 3 Angstroms of the three fluorine atoms (pink) on the drug.*** |
| --- | --- |
| **Word #1 / Clue #3**  ***“I am NOT a hydrophobic amino acid. I am the ONLY non-hydrophobic amino acid that is within 3 Angstroms of the three fluorine atoms (pink) on the drug.*** | **Word #1 / Clue #3**  ***“I am NOT a hydrophobic amino acid. I am the ONLY non-hydrophobic amino acid that is within 3 Angstroms of the three fluorine atoms (pink) on the drug.*** |

| **Word #1 / Clue #4**  ***“I have a polar side chain with an oxygen atom that is 3.45 Angstroms away from one of the drug carbons involved in a triple bond that has a linear molecular geometry.”*** | **Word #1 / Clue #4**  ***“I have a polar side chain with an oxygen atom that is 3.45 Angstroms away from one of the drug carbons involved in a triple bond that has a linear molecular geometry.”*** |
| --- | --- |
| **Word #1 / Clue #4**  ***“I have a polar side chain with an oxygen atom that is 3.45 Angstroms away from one of the drug carbons involved in a triple bond that has a linear molecular geometry.”*** | **Word #1 / Clue #4**  ***“I have a polar side chain with an oxygen atom that is 3.45 Angstroms away from one of the drug carbons involved in a triple bond that has a linear molecular geometry.”*** |

| **Word #1 / Clue #5**  ***“The H66 atom of the drug points directly into the center of my ring. What amino acid am I?”*** | **Word #1 / Clue #5**  ***“The H66 atom of the drug points directly into the center of my ring. What amino acid am I?”*** |
| --- | --- |
| **Word #1 / Clue #5**  ***“The H66 atom of the drug points directly into the center of my ring. What amino acid am I?”*** | **Word #1 / Clue #5**  ***“The H66 atom of the drug points directly into the center of my ring. What amino acid am I?”*** |

| **Word #1 / Clue #6**  ***“Atoms within my backbone form hydrogen bonds with residues 272 and 280.”*** | **Word #1 / Clue #6**  ***“Atoms within my backbone form hydrogen bonds with residues 272 and 280.”*** |
| --- | --- |
| **Word #1 / Clue #6**  ***“Atoms within my backbone form hydrogen bonds with residues 272 and 280.”*** | **Word #1 / Clue #6**  ***“Atoms within my backbone form hydrogen bonds with residues 272 and 280.”*** |

| **Word #1 / Clue #7**  ***“I am a negatively charged amino acid with an oxygen atom that makes a hydrogen bond to atom H56 on the drug. ”*** | **Word #1 / Clue #7**  ***“I am a negatively charged amino acid with an oxygen atom that makes a hydrogen bond to atom H56 on the drug. ”*** |
| --- | --- |
| **Word #1 / Clue #7**  ***“I am a negatively charged amino acid with an oxygen atom that makes a hydrogen bond to atom H56 on the drug. ”*** | **Word #1 / Clue #7**  ***“I have a negatively charged amino acid with an oxygen atom that makes a hydrogen bond to atom H56 on the drug. ”*** |

| **Word #1 / Clue #8**  ***“A backbone hydrogen atom of mine forms a nice hydrogen bond with the backbone oxygen atom of residue 120. I also contain a sulfur atom.”*** | **Word #1 / Clue #8**  ***“A backbone hydrogen atom of mine forms a nice hydrogen bond with the backbone oxygen atom of residue 120. I also contain a sulfur atom.”*** |
| --- | --- |
| **Word #1 / Clue #8**  ***“A backbone hydrogen atom of mine forms a nice hydrogen bond with the backbone oxygen atom of residue 120. I also contain a sulfur atom.”*** | **Word #1 / Clue #8**  ***“A backbone hydrogen atom of mine forms a nice hydrogen bond with the backbone oxygen atom of residue 120. I also contain a sulfur atom.”*** |

| **Word #1 / Clue #9**  ***“My backbone oxygen forms a nice hydrogen bond with the H67 atom of the drug.”*** | **Word #1 / Clue #9**  ***“My backbone oxygen forms a nice hydrogen bond with the H67 atom of the drug.”*** |
| --- | --- |
| **Word #1 / Clue #9**  ***“My backbone oxygen forms a nice hydrogen bond with the H67 atom of the drug.”*** | **Word #1 / Clue #9**  ***“My backbone oxygen forms a nice hydrogen bond with the H67 atom of the drug.”*** |

| **Word #2 / Clue #1**  ***“I form a (covalent) peptide bond with residue 121 – this means that I am right next to residue 121 in the covalently connected chain or string of amino acids. And I am also the same type of amino acid as residue 121!”*** | **Word #2 / Clue #1**  ***“I form a (covalent) peptide bond with residue 121 – this means that I am right next to residue 121 in the covalently connected chain or string of amino acids. And I am also the same type of amino acid as residue 121!”*** |
| --- | --- |
| **Word #2 / Clue #1**  ***“I form a (covalent) peptide bond with residue 121 – this means that I am right next to residue 121 in the covalently connected chain or string of amino acids. And I am also the same type of amino acid as residue 121!”*** | **Word #2 / Clue #1**  ***“I form a (covalent) peptide bond with residue 121 – this means that I am right next to residue 121 in the covalently connected chain or string of amino acids. And I am also the same type of amino acid as residue 121!”*** |

| **Word #2 / Clue #2**  ***“My side chain is hydrophobic and nonpolar. One of my atoms is only 2.62 Angstroms away from a hydrogen atom (H58) on the drug!”*** | **Word #2 / Clue #2**  ***“My side chain is hydrophobic and nonpolar. One of my atoms is only 2.62 Angstroms away from a hydrogen atom (H58) on the drug!”*** |
| --- | --- |
| **Word #2 / Clue #2**  ***“My side chain is hydrophobic and nonpolar. One of my atoms is only 2.62 Angstroms away from a hydrogen atom (H58) on the drug!”*** | **Word #2 / Clue #2**  ***“My side chain is hydrophobic and nonpolar. One of my atoms is only 2.62 Angstroms away from a hydrogen atom (H58) on the drug!”*** |

| **Word #3 / Clue #1**  ***“I am an amino acid that has a carbon atom with tetrahedral geometry that is covalently bonded to three hydrogens. and is close to a ring on the drug. In fact, one of the three hydrogens is 3.14 Angstroms away from atom C5 on the drug.”*** | **Word #3 / Clue #1**  ***“I am an amino acid that has a carbon atom with tetrahedral geometry that is covalently bonded to three hydrogens. and is close to a ring on the drug. In fact, one of the three hydrogens is 3.14 Angstroms away from atom C5 on the drug.”*** |
| --- | --- |
| **Word #3 / Clue #1**  ***“I am an amino acid that has a carbon atom with tetrahedral geometry that is covalently bonded to three hydrogens. and is close to a ring on the drug. In fact, one of the three hydrogens is 3.14 Angstroms away from atom C5 on the drug.”*** | **Word #3 / Clue #1**  ***“I am an amino acid that has a carbon atom with tetrahedral geometry that is covalently bonded to three hydrogens. and is close to a ring on the drug. In fact, one of the three hydrogens is 3.14 Angstroms away from atom C5 on the drug.”*** |

| **Word #3 / Clue #2**  ***“I form a (covalent) peptide bond with residue 86 – this means that I am right next to residue 86 in the connected chain or string of amino acids. And I am also the same type of amino acid as residue 86!”*** | **Word #3 / Clue #2**  ***“I form a (covalent) peptide bond with residue 86 – this means that I am right next to residue 86 in the connected chain or string of amino acids. And I am also the same type of amino acid as residue 86!”*** |
| --- | --- |
| **Word #3 / Clue #2**  ***“I form a (covalent) peptide bond with residue 86 – this means that I am right next to residue 86 in the covalently connected chain or string of amino acids. And I am also the same type of amino acid as residue 86!”*** | **Word #3 / Clue #2**  ***“I form a (covalent) peptide bond with residue 86 – this means that I am right next to residue 86 in the connected chain or string of amino acids. And I am also the same type of amino acid as residue 86!”*** |

| **Word #3 / Clue #3**  ***“I likely have London Dispersion forces (id-id) with the drug. A hydrogen atom in my backbone is only 2.65 Angstroms away from the fluorine atom F35 on the drug.”*** | **Word #3 / Clue #3**  ***“I likely have London Dispersion forces (id-id) with the drug. A hydrogen atom in my backbone is only 2.65 Angstroms away from the fluorine atom F35 on the drug.”*** |
| --- | --- |
| **Word #3 / Clue #3**  ***“I likely have London Dispersion forces (id-id) with the drug. A hydrogen atom in my backbone is only 2.65 Angstroms away from the fluorine atom F35 on the drug.”*** | **Word #3 / Clue #3**  ***“I likely have London Dispersion forces (id-id) with the drug. A hydrogen atom in my backbone is only 2.65 Angstroms away from the fluorine atom F35 on the drug.”*** |

| **Word #3 / Clue #4**  ***“A hydrogen bonded to one of my oxygen atoms forms a hydrogen bond with a water molecule that is “locked” within the protein structure, as many water molecules often are.” (Type “resid 325” to find the water molecule and “same residue as within 4 of resid 325’ to find things close to it!)*** | **Word #3 / Clue #4**  ***“A hydrogen bonded to one of my oxygen atoms forms a hydrogen bond with a water molecule that is “locked” within the protein structure, as many water molecules often are.” (Type “resid 325” to find the water molecule and “same residue as within 4 of resid 325’ to find things close to it!)*** |
| --- | --- |
| **Word #3 / Clue #4**  ***“A hydrogen bonded to one of my oxygen atoms forms a hydrogen bond with a water molecule that is “locked” within the protein structure, as many water molecules often are.” (Type “resid 325” to find the water molecule and “same residue as within 4 of resid 325’ to find things close to it!)*** | **Word #3 / Clue #4**  ***“A hydrogen bonded to one of my oxygen atoms forms a hydrogen bond with a water molecule that is “locked” within the protein structure, as many water molecules often are.” (Type “resid 325” to find the water molecule and “same residue as within 4 of resid 325’ to find things close to it!)*** |

| **Word #3 / Clue #5**  ***“I have a sulfur atom in my side chain. I experience London dispersion forces (id-id) with residue 201.”*** | **Word #3 / Clue #5**  ***“I have a sulfur atom in my side chain. I experience London dispersion forces (id-id) with residue 201.”*** |
| --- | --- |
| **Word #3 / Clue #5**  ***“I have a sulfur atom in my side chain. I experience London dispersion forces (id-id) with residue 201.”*** | **Word #3 / Clue #5**  ***“I have a sulfur atom in my side chain. I experience London dispersion forces (id-id) with residue 201.”*** |

| **Word #3 / Clue #6**  ***“I have a nonpolar & hydrophobic sidechain that contains only one ring. You will find me near residue 178.”*** | **Word #3 / Clue #6**  ***“I have a nonpolar & hydrophobic sidechain that contains only one ring. You will find me near residue 178.”*** |
| --- | --- |
| **Word #3 / Clue #6**  ***“I have a nonpolar & hydrophobic sidechain that contains only one ring. You will find me near residue 178.”*** | **Word #3 / Clue #6**  ***“I have a nonpolar & hydrophobic sidechain that contains only one ring. You will find me near residue 178.”*** |

| **Word #3 / Clue #7**  ***“I have a ring with two nitrogens. One of these nitrogens forms a hydrogen bond (1.82 Angstroms long) with a hydrogen atom of residue 70.”*** | **Word #3 / Clue #7**  ***“I have a ring with two nitrogens. One of these nitrogens forms a hydrogen bond (1.82 Angstroms long) with a hydrogen atom of residue 70.”*** |
| --- | --- |
| **Word #3 / Clue #7**  ***“I have a ring with two nitrogens. One of these nitrogens forms a hydrogen bond (1.82 Angstroms long) with a hydrogen atom of residue 70.”*** | **Word #3 / Clue #7**  ***“I have a ring with two nitrogens. One of these nitrogens forms a hydrogen bond (1.82 Angstroms long) with a hydrogen atom of residue 70.”*** |

| **Word #3 / Clue #8**  ***“One of the two partially positive hydrogens off of a nitrogen atom of mine makes a hydrogen bond (length 2.37 Angstroms) with the partially negative oxygen atom of residue 136."*** | **Word #3 / Clue #8**  ***“One of the two partially positive hydrogens off of a nitrogen atom of mine makes a hydrogen bond (length 2.37 Angstroms) with the partially negative oxygen atom of residue 136."*** |
| --- | --- |
| **Word #3 / Clue #8**  ***“One of the two partially positive hydrogens off of a nitrogen atom of mine makes a hydrogen bond (length 2.37 Angstroms) with the partially negative oxygen atom of residue 136."*** | **Word #3 / Clue #8**  ***“One of the two partially positive hydrogens off of a nitrogen atom of mine makes a hydrogen bond (length 2.37 Angstroms) with the partially negative oxygen atom of residue 136."*** |

| **Word #3 / Clue #9**  ***“My backbone oxygen atom is making a beautiful hydrogen bond (distance 1.85 Angstroms) with a backbone hydrogen of residue 174.”*** | **Word #3 / Clue #9**  ***“My backbone oxygen atom is making a beautiful hydrogen bond (distance 1.85 Angstroms) with a backbone hydrogen of residue 174.”*** |
| --- | --- |
| **Word #3 / Clue #9**  ***“My backbone oxygen atom is making a beautiful hydrogen bond (distance 1.85 Angstroms) with a backbone hydrogen of residue 174.”*** | **Word #3 / Clue #9**  ***“My backbone oxygen atom is making a beautiful hydrogen bond (distance 1.85 Angstroms) with a backbone hydrogen of residue 174.”*** |

| **Word #3 / Clue #10**  ***“I am a nonpolar amino acid. One of my hydrogens is 2.39 Angstroms away from atom H60 on the drug”*** | **Word #3 / Clue #10**  ***“I am a nonpolar amino acid. One of my hydrogens is 2.39 Angstroms away from atom H60 on the drug”*** |
| --- | --- |
| **Word #3 / Clue #10**  ***“I am a nonpolar amino acid. One of my hydrogens is 2.39 Angstroms away from atom H60 on the drug”*** | **Word #3 / Clue #10**  ***“I am a nonpolar amino acid. One of my hydrogens is 2.39 Angstroms away from atom H60 on the drug”*** |
