# Supplementary material for: EMMAs: Implementation and Assessment of a Suite of Cross-Disciplinary, Case-Based High School Activities to Explore Three-Dimensional Molecular Structure, Noncovalent Interactions, and Molecular Dynamics
Source: J Chem Educ. 2024 May 10;101(6):2436–47. doi: 10.1021/acs.jchemed.4c00036 (PMC11171454; doi:10.1021/acs.jchemed.4c00036)
Supplement: Supplementary file 1 — ed4c00036_si_001.zip [file ed4c00036_si_001.zip › Kotsalidis_supporting_info_revisions/07 - Investigation of MD Sims Post-lab.docx]

**“Investigation of Molecular Dynamics Simulations” Post-lab**

**Review**

In the “Investigation of Molecular Dynamics Simulations” activity we learned about both the wild type and T315I mutant Abl kinase proteins and how they may differ. We learned about how mutations in proteins, such as the T315I mutation, can influence both noncovalent interactions (such as hydrogen bonds) and fluctuations in the molecules in the system. As we discussed, ponatinib, a third-generation tyrosine kinase inhibitor, has the potential to help treat patients with this T315I mutation because the drug is not relying on a hydrogen bond interaction with the threonine residue at position 315.

**Does the Hydrogen Bond Really Matter?**

Recent research has speculated and shown that maybe the hydrogen bond isn’t as important as it was once made out to be for the kinase’s resistance to imatinib. In their research, both Schindler et al.^^[[1]](#footnote-0)^^ and Corbin et al.^^[[2]](#footnote-1)^^ found that although the T315I mutation did alter the three-dimensional structure of the protein, they did not find a hydrogen bond critical for imatinib’s ability to bind to the kinase. In order to further investigate these findings, Pricl et al.^^[[3]](#footnote-2)^^ used a molecular modeling level approach. The abstract from their paper is shown below. In a scientific paper, abstracts summarize the major aspects of the entire paper in a short paragraph or two. They describe the overall purpose of the research, how they designed the experiments and the major findings from their study.

***T315I-mutated Bcr-Abl in Chronic Myeloid Leukemia and Imatinib: Insights from a Computational Study***

“The early stage of chronic myeloid leukemia is triggered by the tyrosine kinase Bcr-Abl. Imatinib mesylate, a selective inhibitor of Bcr-Abl, has been successful in chronic myeloid leukemia clinical trials, but short-lived remissions are usually observed in blast crisis patients. Sequencing of the BCR-ABL gene in relapsed patients revealed a set of mutants that mediate drug resistance. Previously reported work postulated that the missense T315I mutation both alters the three-dimensional structure of the protein binding site, thus decreasing the protein sensitivity for the drug, and does not feature a fundamental hydrogen bond that is critical for binding with imatinib. These speculations, however, were not supported by investigations at the molecular modeling level. Here, we present the results obtained from the application of molecular dynamics simulations to the study of the interactions between T315I Bcr-Abl and imatinib. For the first time, we show that, with respect to the wild-type system, the absence of the supposedly critical H-bond is not the only cause for the failure of receptor inhibition by imatinib, but also a plethora of other protein/drug interactions are drastically and unfavorably changed in the mutant protein.”

1. What do the authors say was already known previously before this paper was published?

|  |
| --- |

1. What do the author’s state is their purpose in conducting their research?

|  |
| --- |

1. What were the main findings in this study?

|  |
| --- |

Science is an ongoing process with new discoveries happening every day. Whether it be about the discovery of a new molecular pathway in a disease or the discovery of a new drug to treat the disease, science is always evolving.

1. Schindler, T., Bornmann, W., Pellicena, P., Miller, W. T., Clarkson, B., and Kuriyan, J. (2000) Structural Mechanism for STI-571 Inhibition of Abelson Tyrosine Kinase. Science 289, 1938–1942. [↑](#footnote-ref-0)
2. Corbin, A. S., Buchdunger, E., Pascal, F., and Druker, B. J. (2002) Analysis of the Structural Basis of Specificity of Inhibition of the Abl Kinase by STI571. J. Biol. Chem. 277, 32214–32219. [↑](#footnote-ref-1)
3. Pricl, S., Fermeglia, M., Ferrone, M., and Tamborini, E. (2005) T315I-mutated Bcr-Abl in chronic myeloid leukemia and imatinib: insights from a computational study. Mol. Cancer Ther. 4, 1167–1174. [↑](#footnote-ref-2)
