# Supplementary material for: EMMAs: Implementation and Assessment of a Suite of Cross-Disciplinary, Case-Based High School Activities to Explore Three-Dimensional Molecular Structure, Noncovalent Interactions, and Molecular Dynamics
Source: J Chem Educ. 2024 May 10;101(6):2436–47. doi: 10.1021/acs.jchemed.4c00036 (PMC11171454; doi:10.1021/acs.jchemed.4c00036)
Supplement: Supplementary file 1 — ed4c00036_si_001.zip [file ed4c00036_si_001.zip › Kotsalidis_supporting_info_revisions/A-HELPFUL VMD COMMANDS.pdf]

Helpful VMD Commands

**R** + click + hold	rotate
**T**+ click + hold	translate
**S** + click + hold	zoom
**C** + click + **R**	center + rotate

**1** + click atom	**identify** atom or amino acid
**2** + click (2) atoms	**distance** between two atoms
**3** + click (3) atoms	**angle** between three atoms

**Screenshot**
**mac =**<br>command + shift + 3 (whole screen)<br>command + shift + 4 (select specific area)<br><br>**pc=**<br>Windows key + shift + s<br>snipping tool

Atom Color	Atom Type
Turquoise	Carbon
White	Hydrogen
Red	Oxygen
Blue	Nitrogen
Pink	Fluorine
Yellow	Sulfur

"chain______"

D = drug
A= protein
W= water

"resid______"

insert number to find amino acid

"name______"

- ➔ Insert atom symbol and number
- ➔ Finds a specific atom
- ➔ ex. "**name** H57" for hydrogen number 57

"same resid as within______ of chain D"

- ➔ Insert distance (usually 2-4)
- ➔ Finds amino acids that are a certain distance from the drug.

"same resid as within______ of resid______"

- ➔ Insert distance (usually 2-4)
- ➔ Insert resid numbers
- ➔ Find amino acids that are a certain distance from another amino acid.
