# Supplementary material for: EMMAs: Implementation and Assessment of a Suite of Cross-Disciplinary, Case-Based High School Activities to Explore Three-Dimensional Molecular Structure, Noncovalent Interactions, and Molecular Dynamics
Source: J Chem Educ. 2024 May 10;101(6):2436–47. doi: 10.1021/acs.jchemed.4c00036 (PMC11171454; doi:10.1021/acs.jchemed.4c00036)
Supplement: Supplementary file 1 — ed4c00036_si_001.zip [file ed4c00036_si_001.zip › Kotsalidis_supporting_info_revisions/C - EMMAs Learning Outcomes for Activitiies.docx]

Remember

Understand

Apply

Analyze

Evaluate

**Activity 1: CML Case Study**

Students should be able to:

● Explain how a genetic mutation leads to a change in protein function.

● Explain how a drug molecule, through physically interacting with a target, can alter the target’s function.

● Explain how a biomolecular change can lead to a disease.

**Activities 2a and 2b: Exploring ponatinib and Abl kinase**

Students should be able to:

● Recall that proteins are polymers of amino acids that can form secondary and tertiary structures.

● Recognize that atoms are the basic building blocks of molecules.

● Know how to rotate, translate, and zoom in/out and center the molecules of interest.

● Compare and contrast different coloring and drawing methods in VMD.

● Select relevant parts of the molecules using VMD.

● Determine the distances between atoms using VMD.

● Compare and contrast the value of using certain graphical representations to answer relevant questions.

● Identify molecular geometries visually.

**Activity 3: CML Stories Investigation**

Students should be able to:

● Provide an example of a person with CML and summarize some of their experiences (symptoms, diagnosis, etc.).

● Explain the connection between biomolecular processes and changes and disease.

● Reflect and comment on aspects of the athlete’s story that make a personal impression on them.

**Activity 4: Exploring the Ponatinib-Abl kinase complex**

Students should be able to:

● Manipulate the molecule through rotation, translation and zooming in/out in order to answer other biochemical questions.

● Compare and contrast the different drawing methods in VMD in the context of visualization.

● Select relevant parts of the molecules using VMD.

● Determine the distances between atoms using VMD.

● Create multiple representations to visualize multiple molecules simultaneously.

● Evaluate choices of representations when visualizing molecular interactions.

● Identify hydrogen bonding within the ponatinib-Abl kinase complex.

**Activity 5: Cracking the Secret Code**

Students should be able to:

● Compare and contrast the different coloring and drawing methods in VMD.

● Use VMD commands to aid in molecule selection.

● Illustrate the difference between graphical representations by creating multiple representations.

● Recognize amino acids.

● Connect amino acid structure with their physical and chemical properties.

● Identify hydrogen bonding within the ponatinib-Abl kinase complex.

● Recognize important kinase residues that interact closely with the drug.

● Determine the distance between two atoms.

● Recall what constitutes covalent bonds, hydrogen bonding interactions and hydrophobic interfaces.

● Describe the structure of amino acids.

● Explain the importance of biological interactions, both covalent and noncovalent, in protein-drug interactions.

**Activity 6: Molecular Simulation Video Modules**

Students should be able to:

● Describe what an MD simulation is and what it shows.

● Outline the important steps in creating an MD simulation.

● Articulate the importance of MD simulations in biological systems.

● Relate MD simulations to a biological system outside of those discussed.

● Explain applications of Newton’s Laws to create an MD simulation.

● Articulate basic physical concepts relating to electrostatic interactions and force.

● Explain the importance of small time steps when creating a trajectory.

**(Ongoing work: Activity 7: Investigations of MD Simulations)**

Students should be able to:

● Summarize the significance of MD simulations in scientific research.

● Apply VMD skills to a dynamic trajectory.

● Predict how MD simulations can be used in drug design.

● Compare and contrast the structural and binding aspects of ponatinib and imatinib.

● Generate hydrogen bonding graphs.

● Analyze hydrogen bonding graphs.

● Evaluate and weigh different and sometimes contradictory evidence in addressing scientific questions.

● Analyze distance and fluctuation analysis graphs.

● Justify the importance of data analysis in understanding drug-target interactions.

● Recognize the dynamic nature of molecules.

● Explain ways that science is interdisciplinary.

● Identify the purpose, and main findings from a primary literature article abstract.

● Explain how science is an ongoing process.

● Evaluate and weigh different and sometimes contradictory evidence in addressing scientific question.
