# Supplementary material for: EMMAs: Implementation and Assessment of a Suite of Cross-Disciplinary, Case-Based High School Activities to Explore Three-Dimensional Molecular Structure, Noncovalent Interactions, and Molecular Dynamics
Source: J Chem Educ. 2024 May 10;101(6):2436–47. doi: 10.1021/acs.jchemed.4c00036 (PMC11171454; doi:10.1021/acs.jchemed.4c00036)
Supplement: Supplementary file 1 — ed4c00036_si_001.zip [file ed4c00036_si_001.zip › Kotsalidis_supporting_info_revisions/II - Kotsalidis_supp_info_3_computational_method_details.docx]

**SUPPORTING INFORMATION PART III**

**for**

**EMMAs: A Suite of Cross-Disciplinary, Cased-Based High School Activities to Explore Three-Dimensional Molecular Structure, Noncovalent Interactions, and Molecular Dynamics**

**Supporting Information Related to Computational Methods Used to Generate Activities:**

**Computational Details:** The static ponatinib/Abl kinase structure file used for Activities 2, 4, and 5 was prepared from a crystallographic model (PDB ID 3OXZ) (Zhou et al., 2011). Missing residues were built using the UCSF MODELLER/CHIMERA package (Pettersen et al., 2004), and water molecules making fewer than 3 potential hydrogen bond contacts within 3.3 Angstroms of drug or protein were removed. The amide groups of one GLN/ASN side chain (GLN 252) was flipped based on visual inspection of hydrogen bonding contacts, and all HIS side chains were modeled as the epsilon tautomer. Hydrogens atoms were added using the HBUILD facility (Brunger and Karplus, 1988) within the CHARMM molecular modeling software package (Brooks et al., 1983) and the CHARMm22 force field (Momany and Rone, 1992). In between an initial two rounds of running HBUILD and a subsequent two-round run, all atoms not original to the crystal structure were minimized via 500 steps of minimization. Ponatinib point charges were estimated using the RESP procedure (Bayly et al., 1993) after geometry optimization at the HF/6-31g level and electrostatic potentials obtained at the HF/6-31g* level using Gaussian03 (Frisch, 2004). For both ponatinib and imatinib, the nitrogen of the methylpiperazine group was assumed to be protonated (Aleksandrov and Simonson, 2010).

The 100ns MD simulations of the imatinib/Abl kinase complex in explicit solvent used to generate the movies in activities 6 and 7 were carried out using the GROMACS software package (Abraham et al., 2015) with the CHARMM36 force field (Huang and MacKerell Jr, 2013) and the SPC and TIP3P water models (Jorgensen et al., 1983). The initial models for the simulations were based on a crystallographic model (PDB ID 2HYY (Cowan-Jacob et al., 2007)) that was prepared using MODELLER/CHIMERA, with drug charges obtained in a similar manner to that for ponatinib above, except a two-stage RESP procedure was used here. Additional atom types and bond, angle, and dihedral parameters were obtained using CGenFF (Vanommeslaeghe et al., 2010). For the simulation with mutated Abl kinase, PyMOL (Schrodinger, 2015) was used to introduce a T315I mutation. Details for the simulation parameters can be found in (Audil, 2020) and (Trinh, 2020).

Abraham, M. J.,Murtola, T.,Schulz, R.,Páll, S.,Smith, J. C.,Hess, B. & Lindahl, E. (2015). GROMACS: High performance molecular simulations through multi-level parallelism from laptops to supercomputers. *SoftwareX,* 1-2**,** 19-25.

Aleksandrov, A. & Simonson, T. (2010). A molecular mechanics model for imatinib and imatinib:kinase binding. *Journal of Computational Chemistry,* 31**,** 1550-1560.

Audil, A. 2020. *Optimizing Drug-Target Electrostatic Interactions Using Molecular Dynamic (MD) Simulations: A Computational Approach to Investigating the Wildtype Chronic Myeloid Leukemia System.* B.A., Wellelsey College.

Bayly, C. I.,Cieplak, P.,Cornell, W. D. & Kollman, P. A. (1993). A well-behaved electrostatic potential based method using charge restraints for deriving atomic charges - The RESP model. *Journal of Physical Chemistry,* 97**,** 10269-10280.

Brooks, B. R.,Bruccoleri, R. E.,Olafson, B. D.,States, D. J.,Swaminathan, S. & Karplus, M. (1983). CHARMM - A program for macromolecular energy, minimization, and dynamics calculations. *Journal of Computational Chemistry,* 4**,** 187-217.

Brunger, A. T. & Karplus, M. (1988). Polar hydrogen positions in proteins - Empirical energy placement and neutron-diffraction comparison. *Proteins-Structure Function and Genetics,* 4**,** 148-156.

Cowan-Jacob, S. W.,Fendrich, G.,Floersheimer, A.,Furet, P.,Liebetanz, J.,Rummel, G.,Rheinberger, P.,Centeleghe, M.,Fabbro, D. & Manley, P. W. (2007). Structural biology contributions to the discovery of drugs to treat chronic myelogenous leukaemia. *Acta Crystallographica Section D,* 63**,** 80-93.

Frisch, M. J. T., G. W.; Schlegel, H. B.; Scuseria, G. E.; Robb, M. A.; Cheeseman, J. R.; Montgomery, Jr., J. A.; Vreven, T.; Kudin, K. N.; Burant, J. C.; Millam, J. M.; Iyengar, S. S.; Tomasi, J.; Barone, V.; Mennucci, B.; Cossi, M.; Scalmani, G.; Rega, N.; Petersson, G. A.; Nakatsuji, H.; Hada, M.; Ehara, M.; Toyota, K.; Fukuda, R.; Hasegawa, J.; Ishida, M.; Nakajima, T.; Honda, Y.; Kitao, O.; Nakai, H.; Klene, M.; Li, X.; Knox, J. E.; Hratchian, H. P.; Cross, J. B.; Bakken, V.; Adamo, C.; Jaramillo, J.; Gomperts, R.; Stratmann, R. E.; Yazyev, O.; Austin, A. J.; Cammi, R.; Pomelli, C.; Ochterski, J. W.; Ayala, P. Y.; Morokuma, K.; Voth, G. A.; Salvador, P.; Dannenberg, J. J.; Zakrzewski, V. G.; Dapprich, S.; Daniels, A. D.; Strain, M. C.; Farkas, O.; Malick, D. K.; Rabuck, A. D.; Raghavachari, K.; Foresman, J. B.; Ortiz, J. V.; Cui, Q.; Baboul, A. G.; Clifford, S.; Cioslowski, J.; Stefanov, B. B.; Liu, G.; Liashenko, A.; Piskorz, P.; Komaromi, I.; Martin, R. L.; Fox, D. J.; Keith, T.; Al-Laham, M. A.; Peng, C. Y.; Nanayakkara, A.; Challacombe, M.; Gill, P. M. W.; Johnson, B.; Chen, W.; Wong, M. W.; Gonzalez, C.; and Pople, J. A 2004. *Gaussian 03, Revision E.01* Gaussian, Inc., Wallingford, CT.

Huang, J. & Mackerell Jr, A. D. (2013). CHARMM36 all-atom additive protein force field: Validation based on comparison to NMR data. *Journal of Computational Chemistry,* 34**,** 2135-2145.

Jorgensen, W. L.,Chandrasekhar, J.,Madura, J. D.,Impey, R. W. & Klein, M. L. (1983). Comparison of simple potential functions for simulating liquid water. *Journal of Chemical Physics,* 79**,** 926-935.

Momany, F. A. & Rone, R. (1992). Validation of the general-purpose QUANTA(R)3.2/CHARMM(R) force-field. *Journal of Computational Chemistry,* 13**,** 888-900.

Pettersen, E. F.,Goddard, T. D.,Huang, C. C.,Couch, G. S.,Greenblatt, D. M.,Meng, E. C. & Ferrin, T. E. (2004). UCSF Chimera--a visualization system for exploratory research and analysis. *J Comput Chem,* 25**,** 1605-12.

Schrodinger, Llc 2015. The PyMOL Molecular Graphics System, Version 1.8.

Trinh, C. M. 2020. *Effects of Molecular Dynamics on the Electrostatic Interactions between Bcr-Abl Kinase and the*

*Chronic Myeloid Leukemia Drug Imatinib: A Computational Study.* B.A., Wellesley College.

Vanommeslaeghe, K.,Hatcher, E.,Acharya, C.,Kundu, S.,Zhong, S.,Shim, J.,Darian, E.,Guvench, O.,Lopes, P.,Vorobyov, I. & Mackerell Jr, A. D. (2010). CHARMM general force field: A force field for drug-like molecules compatible with the CHARMM all-atom additive biological force fields. *Journal of Computational Chemistry,* 31**,** 671-690.

Zhou, T.,Commodore, L.,Huang, W.-S.,Wang, Y.,Thomas, M.,Keats, J.,Xu, Q.,Rivera, V. M.,Shakespeare, W. C.,Clackson, T.,Dalgarno, D. C. & Zhu, X. (2011). Structural Mechanism of the Pan-BCR-ABL Inhibitor Ponatinib (AP24534): Lessons for Overcoming Kinase Inhibitor Resistance. *Chemical Biology & Drug Design,* 77**,** 1-11.
