# Supplementary material for: EMMAs: Implementation and Assessment of a Suite of Cross-Disciplinary, Case-Based High School Activities to Explore Three-Dimensional Molecular Structure, Noncovalent Interactions, and Molecular Dynamics
Source: J Chem Educ. 2024 May 10;101(6):2436–47. doi: 10.1021/acs.jchemed.4c00036 (PMC11171454; doi:10.1021/acs.jchemed.4c00036)
Supplement: Supplementary file 1 — ed4c00036_si_001.zip [file ed4c00036_si_001.zip › Kotsalidis_supporting_info_revisions/Supporting Information TItle Page.pdf]

Supplementary Information for:

EMMAs: Implementation and Assessment of a Suite of Cross-Disciplinary, Case-Based High School Activities to Explore Three-Dimensional Molecular Structure, Noncovalent Interactions, and Molecular Dynamics

Parthena E. Kotsalidis[1], Shelby N. Kranc[2,4], Martin Berryman[3,5], Mala L. Radhakrishnan[1,2*],

Donald E. Elmore[1,2*]

1. Biochemistry Program, Wellesley College, Wellesley, MA 02481.
2. Chemistry Department, Wellesley College, Wellesley, MA 02481.
3. Lincoln-Sudbury Regional High School, Sudbury, MA 01776
4. Current address: Pioneer Charter School of Science, Everett, MA 02149
5. Current address: Malden High School, Malden, MA 02148

* Denotes co-corresponding authors: mradhakr@wellesley.edu (MLR) and

delmore@wellesley.edu (DEE)

Table of contents for files included in Supporting Information:

00 – Summary of EMMAs

00 - Instructions for downloading and installing Visual Molecular Dynamics Software

00 - Folder of VMD Files

01 - Chronic Myeloid Leukemia Case Study

02 - VMD Ponatinib & Abl Kinase Chem 1

02 - VMD Ponatinib & Abl Kinase Chem 2

03 - CML Stories Investigation

04 - VMD Ponatinib & Abl Kinase Interactions Chem 1

04 - VMD Ponatinib & Abl Kinase Interactions Chem 2

05 - Secret Code Activity Form

05 - Secret Code Activity Clues (Task Cards)

05 - Secret Code Website

06 - Molecular Dynamics EdPuzzle 1 Video

06 - Molecular Dynamics EdPuzzle 2 Video

06 – Molecular Dynamics EdPuzzle Questions

07 - Investigations of MD Simulations Activity

07 - Investigation of MD Simulations Post-Lab

A-HELPFUL VMD COMMANDS

B-AMINO ACIDS IN PROTEINS HANDOUT

C – EMMAs Learning Outcomes for Activities

D – EMMAs State_NGSS_AP College Board Items Addressed

I - Kotsalidis_supp_info_2_survey_instruments

II - Kotsalidis_supp_info_3_computational_method_details

III - SPSS Output for Kotsalidis et al
