# Supplementary material for: EMMAs: Implementation and Assessment of a Suite of Cross-Disciplinary, Case-Based High School Activities to Explore Three-Dimensional Molecular Structure, Noncovalent Interactions, and Molecular Dynamics
Source: J Chem Educ. 2024 May 10;101(6):2436–47. doi: 10.1021/acs.jchemed.4c00036 (PMC11171454; doi:10.1021/acs.jchemed.4c00036)
Supplement: Supplementary file 1 — ed4c00036_si_001.zip [file ed4c00036_si_001.zip › Kotsalidis_supporting_info_revisions/03 - Chronic Myeloid Leukemia Stories Investigation.docx]

**Chronic Myeloid Leukemia Stories Investigation**

**CML Story #1**

[
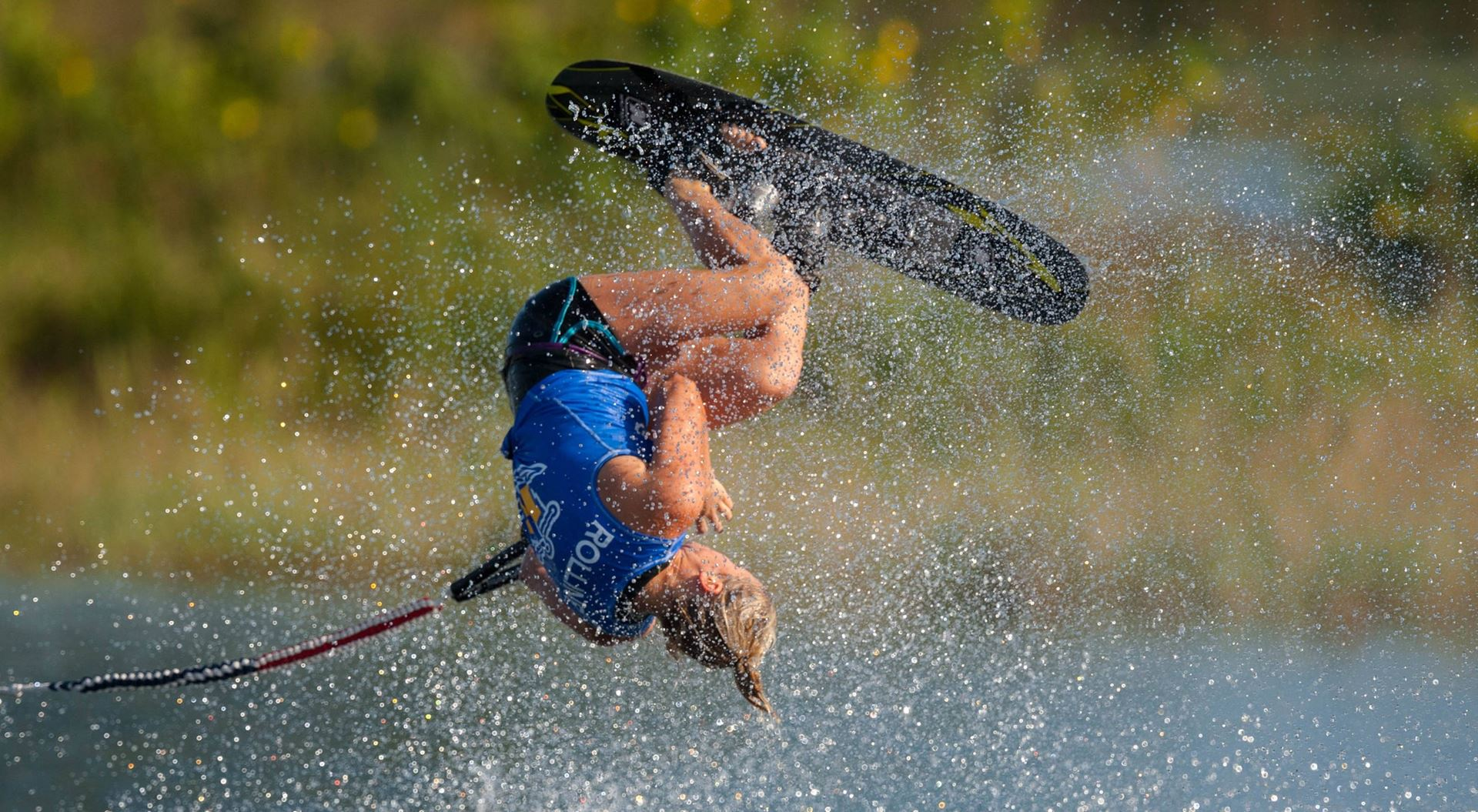
](http://www.alexarscore.com/cancer)

[Alexa Score Wakeboarding](http://www.alexarscore.com/cancer)

**CML Story #2**

[
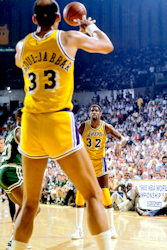
](https://www.webmd.com/cancer/lymphoma/news/20091110/kareem-abdul-jabbar-leukemia-is-cml)

[Kareem Abdul-Jabbar Basketball](https://www.espn.com/nba/news/story?id=4640518)

Steve Lipofsky www.Basketballphoto.com

**CML Story #3**

[
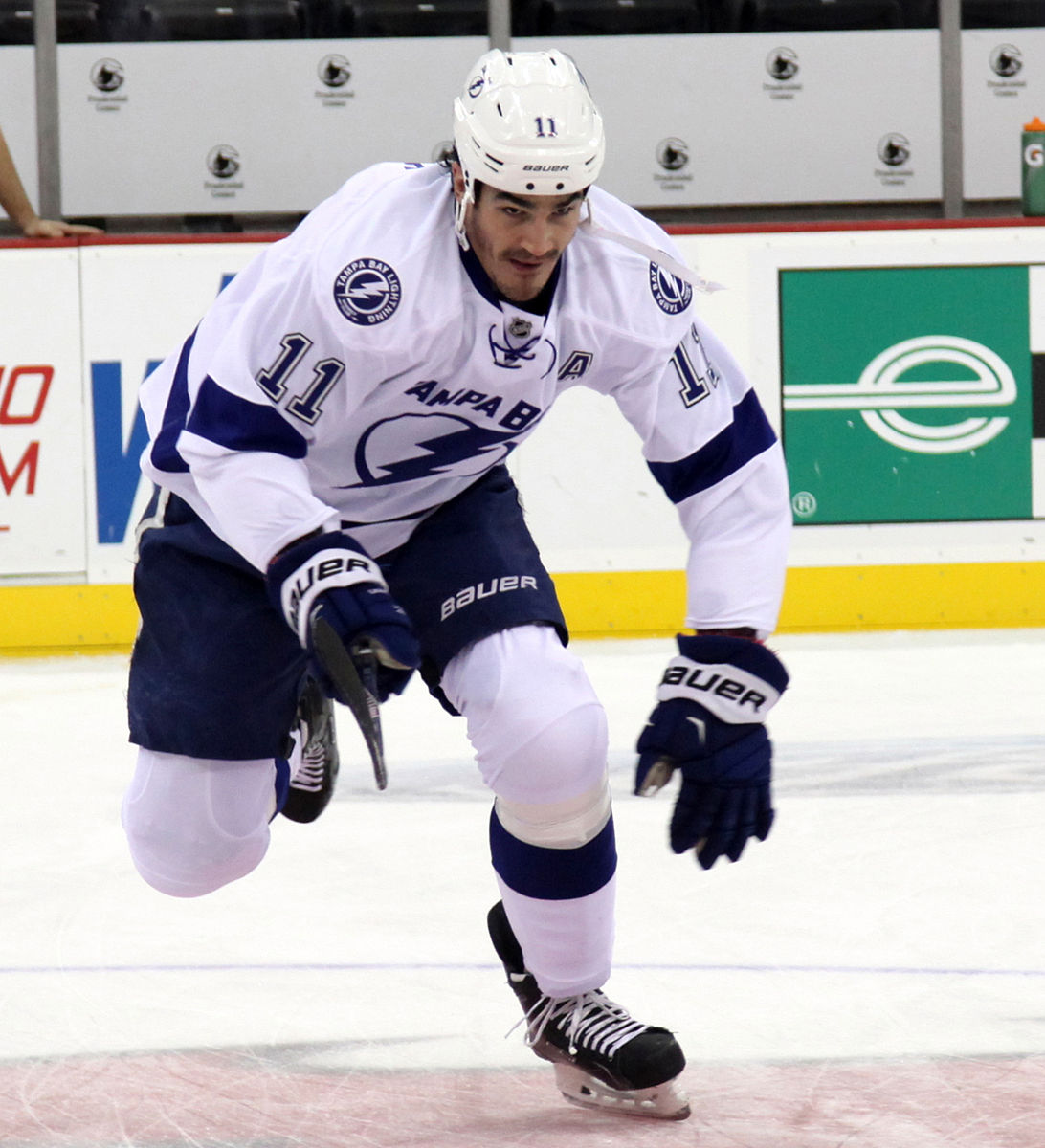
](https://www.nhl.com/news/new-jersey-devils-brian-boyle-has-leukemia/c-291181794)

[Brian Boyle Hockey](https://www.nhl.com/news/new-jersey-devils-brian-boyle-has-leukemia/c-291181794)

Photo by Lisa Gansky

**CML Story #4**

[
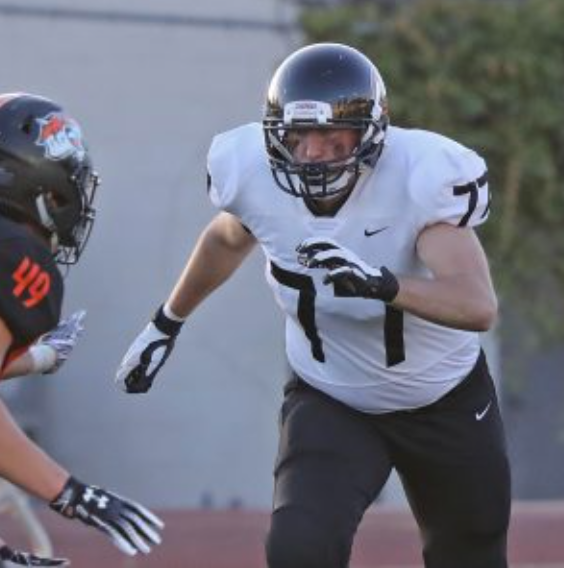
](https://news.chapman.edu/2019/10/31/the-heart-of-a-panther/)

[Hunter Spriggs Football](https://news.chapman.edu/2019/10/31/the-heart-of-a-panther/)

1. Choose one of the athlete’s stories pictured above. Read through the article and answer the following questions:

1. List a few symptoms the athlete experienced before being diagnosed with CML.

| Delete this text and type your answer here. |
| --- |

1. How did the athlete learn about their diagnosis? (blood test, bone marrow smear, aspiration,...)

| Delete this text and type your answer here. |
| --- |

(c) How did the athlete process their diagnosis? Did they stop playing? Did they persevere?

| Delete this text and type your answer here. |
| --- |

(d) Describe one additional observation about the athlete’s story that impressed, surprised, or made an impression on you.

| Delete this text and type your answer here. |
| --- |

| 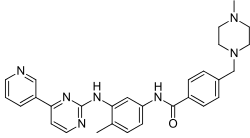  Structure of imatinib (Gleevac)  C_29_H_31_N_7_O | 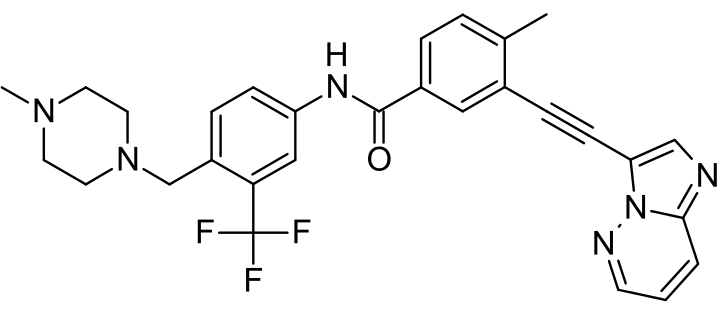  Structure of ponatinib (Iclusig)  C_29_H_27_F_3_N_6_O |
| --- | --- |

2. Above are structural pictures of two tyrosine kinase inhibitor drugs we explored in the case study and computer lab explorations.

1. Describe or copy/paste two features that are the same between the two molecules.

*Example. Each molecule contains 29 carbon atoms*

| Delete this text and type your answer here. |
| --- |

1. Describe or copy/paste two features that are different between the two molecules.

*Example.The ponatinib molecule contains fluorine atoms, while imatinib does not.*

| Delete this text and type your answer here. |
| --- |

3. Think back on the case study/edpuzzle assignment and our explorations with VMD software in the computer lab.

1. What are a few things you learned or liked about the experiences?

| Delete this text and type your answer here. |
| --- |

1. Was there anything that surprised you?

| Delete this text and type your answer here. |
| --- |

(c) Was there anything that was a challenge for you?

| Delete this text and type your answer here. |
| --- |

(d) Do you think you might be more interested in considering a STEM career after learning about Chronic Myeloid Leukemia through modeling?

| Delete this text and type your answer here. |
| --- |
