# Supplementary material for: EMMAs: Implementation and Assessment of a Suite of Cross-Disciplinary, Case-Based High School Activities to Explore Three-Dimensional Molecular Structure, Noncovalent Interactions, and Molecular Dynamics
Source: J Chem Educ. 2024 May 10;101(6):2436–47. doi: 10.1021/acs.jchemed.4c00036 (PMC11171454; doi:10.1021/acs.jchemed.4c00036)
Supplement: Supplementary file 1 — ed4c00036_si_001.zip [file ed4c00036_si_001.zip › Kotsalidis_supporting_info_revisions/01 - Chronic Myeloid Leukemia Case Study.docx]

**Chronic Myeloid Leukemia Case Study**

Imagine you are a third year medical student working in the cancer unit. Sandra, a 40 year old woman, has come in because she got a call from her doctor that her blood test came back with some concerning results. After speaking with Sandra you learn that over the past month she has been feeling very fatigued and has had some weight loss. You order an additional blood test called a complete blood count (CBC) and notice her white blood cell count is very high. A typical healthy person has a white blood cell count ranging from 4,000-11,000 white blood cells.^^[[1]](#footnote-0)^^ Sandra’s white blood cell count however, is 200,000! Unfortunately, it is likely that Sandra has a form of cancer called Chronic Myeloid Leukemia.

Chronic Myeloid Leukemia (CML) is a cancer of the myeloid cells, which are cells that make red blood cells, platelets and most types of white blood cells.^^[[2]](#footnote-1)^^ Below is the picture of a bone marrow smear which is another method for diagnosing a patient with CML.


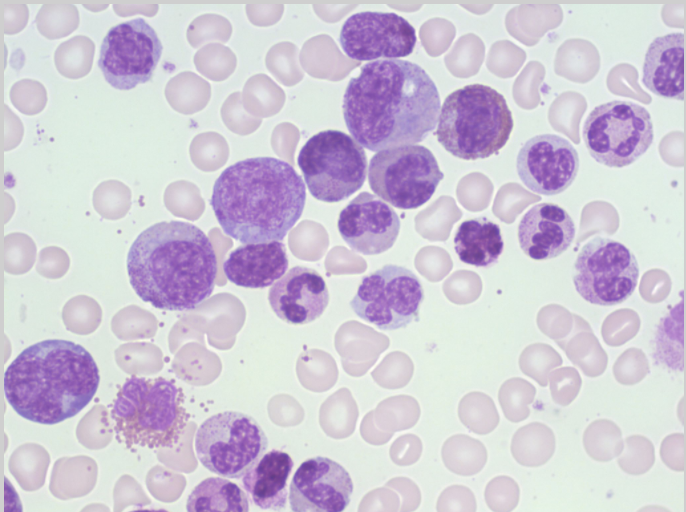


An image from a bone marrow smear demonstrating myeloid hyperplasia in CML. Hyperplasia is defined as an increase in the growth and reproduction of cells, leading to the enlargement of an organ or tissue. It characterizes the initial stages in cancer development.

100x oil immersion. From MLS Collection, University of Alberta, <https://doi.org/10.7939/R34X54Z0H>

CML arises from a chromosomal abnormality. In other words, part of the DNA from one chromosome moves to another chromosome. Chromosomes are made up of proteins and DNA and they store the genetic material. In CML, there is a swapping, or translocation, of chromosomes. In this case, part of chromosome 22 moves to chromosome 9, causing chromosome 22 to be shorter (this is called the [Philadelphia chromosome](https://www.youtube.com/watch?v=e4Uz3MLfhZs)).


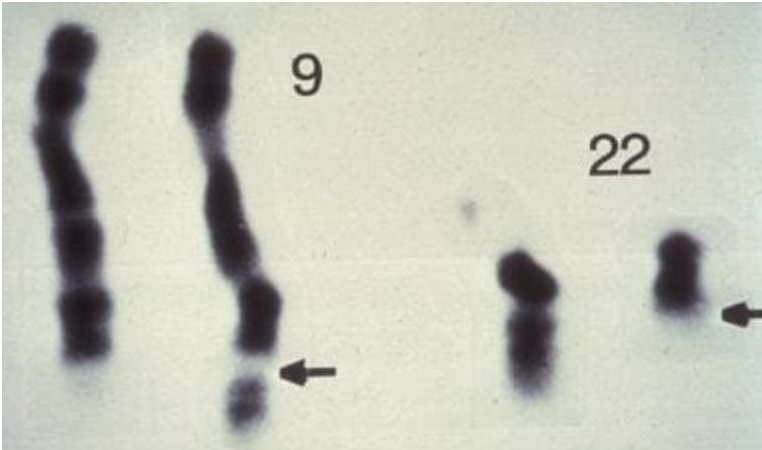


The results of the reciprocal translocation found in CML. Part of chromosome 22 moves to the lower arm of chromosome 9.

Peter C. Nowell, MD, Department of Pathology and Clinical Laboratory of the University of Pennsylvania School of Medicine.

The Abl kinase is a protein in the body that helps regulate processes involved with cell growth and division. The movement of DNA from one chromosome to another leads to the production of the BCR-ABL1 gene, an abnormal and cancerous gene that codes for an overactive kinase that causes too many white blood cells to be produced. This means that the protein is always “on” at all times. The behavior of this overactive kinase is due to the binding of ATP to the binding pocket.

**1.** Identify two tests from the reading that a doctor in the oncology unit could perform to diagnose a patient with Chronic Myeloid Leukemia (CML). (1 point)

|  |
| --- |

**2.** Chronic Myeloid Leukemia (CML) arises from a __________________________________. (1 point)

**3.** Explain how you know that Sandra likely has Chronic Myeloid Leukemia? (2 points)

|  |
| --- |

**4.** Complete the Edpuzzle below to learn more about Chronic Myeloid Leukemia.

[Edpuzzle #1: What is Chronic Myeloid Leukemia (CML)?](https://edpuzzle.com/media/6468d2ba1f972242e39aabef)

Patients with cancer are producing more white blood cells than they need due to the overactive BCR-ABL gene. As you saw in the Edpuzzle video, highly effective drugs have been developed to inhibit the function of this overactive kinase in patients with CML. One such drug is Imatinib. The Edpuzzle video explained how Imatinib binds to the Abl kinase and inhibits its function. Imatinib works by fitting into the binding pocket of the Abl kinase, the same binding pocket ATP would bind in. When imatinib binds, the Abl kinase can no longer bind the ATP molecule it needs to function. As a result, the overactive kinase gets turned off”.

**5.** Below are the structural formula and ball and stick model for the Imatinib molecule.


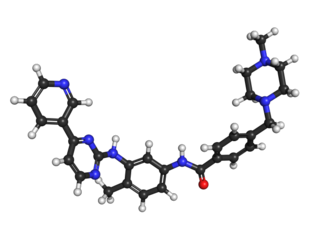


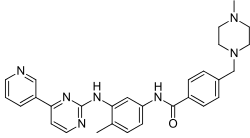


**Structural formula Ball and Stick Model**

**6.** Identify **two** structural features of the Imatinib molecule that interest you. (1 point)

|  |
| --- |

**7.** In terms of intermolecular forces, why is the Imatinib drug soluble in water? (1 point)

|  |
| --- |

**8.** Complete the Edpuzzle below to learn more about how Imatinib blocks the cancer causing ABL-1 kinase.

[Edpuzzle #2: Imatinib (Gleevec)](https://edpuzzle.com/media/6468d19abba7a942c8a19c36)

During the next class, you will learn how to use Visual Molecular Dynamics (VMD), a molecular visualization software, to analyze the Abl kinase protein and another drug that treats CML called Ponatinib.


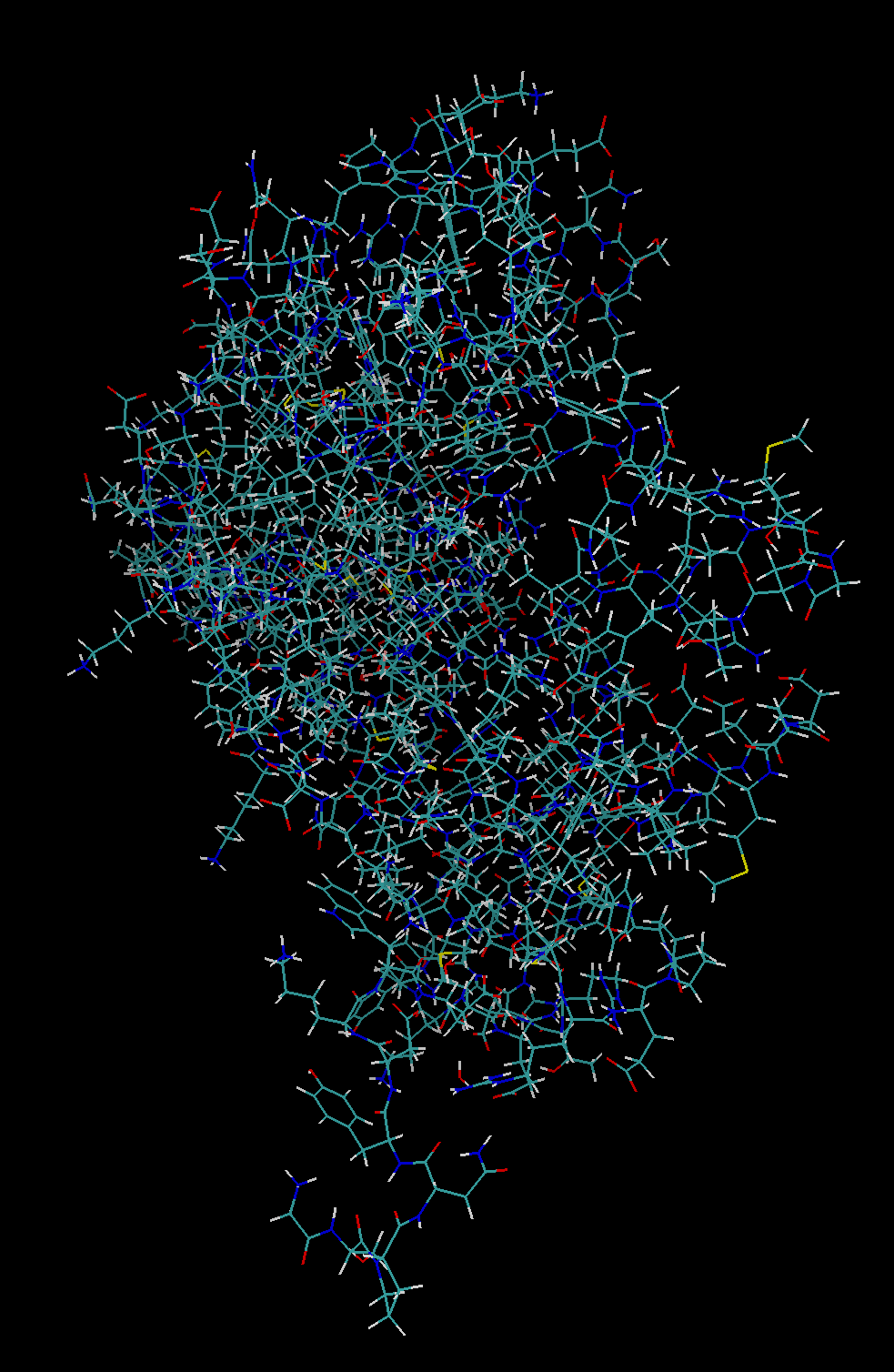


VMD representation of ABL kinase

1. Wang, Eunice. “How Fast Does Leukemia Develop?” Roswell Park Comprehensive Cancer Center, 4 Oct. 2018, <https://www.roswellpark.org/cancertalk/201810/how-fast-does-leukemia-develop>. [↑](#footnote-ref-0)
2. “What Is Chronic Myeloid Leukemia?: Leukemia Types.” *American Cancer Society*, 19 June 2018, https://www.cancer.org/cancer/chronic-myeloid-leukemia/about/what-is-cml.html. [↑](#footnote-ref-1)
