# Supplementary material for: EMMAs: Implementation and Assessment of a Suite of Cross-Disciplinary, Case-Based High School Activities to Explore Three-Dimensional Molecular Structure, Noncovalent Interactions, and Molecular Dynamics
Source: J Chem Educ. 2024 May 10;101(6):2436–47. doi: 10.1021/acs.jchemed.4c00036 (PMC11171454; doi:10.1021/acs.jchemed.4c00036)

Amino Acids in Proteins

Electrically Charged Side Chains

Positive (Basic)

Arginine (Arg) R

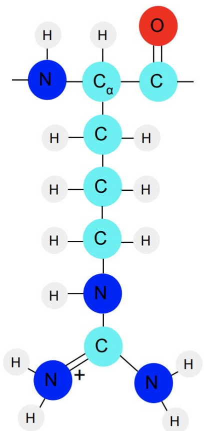

Histidine (His) H

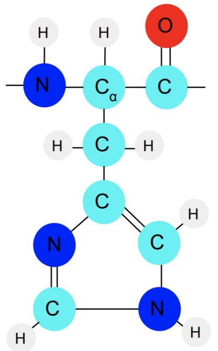

Lysine (Lys) K

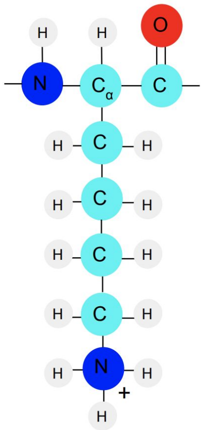

Negative (Acidic)

Glutamic Acid (Glu) E

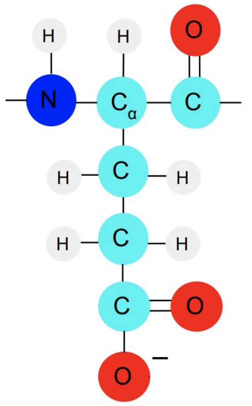

Aspartic Acid (Asp) D

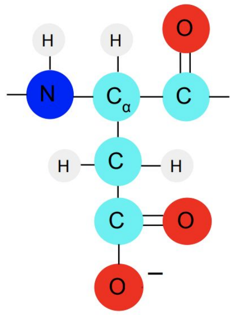

Polar & Hydrophilic Uncharged Side Chains

Serine (Ser) S

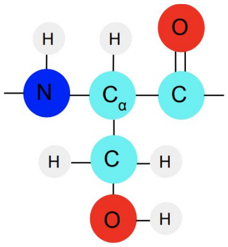

Asparagine (Asn) N

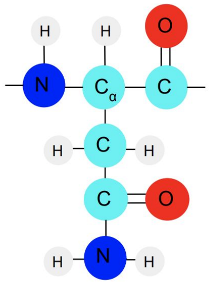

Tyrosine (Tyr) Y

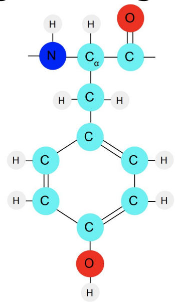

Threonine (Thr) T

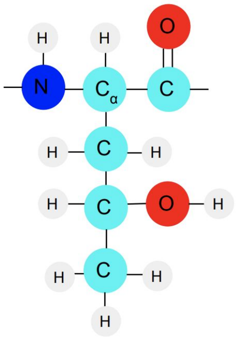

Glutamine (Gln) Q

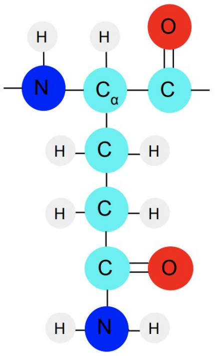

Nonpolar & Hydrophobic Side Chains

Glycine (Gly) G

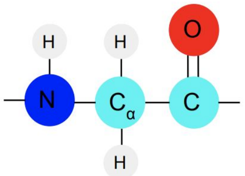

Alanine (Ala) A

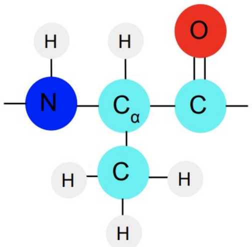

Valine (Val) V

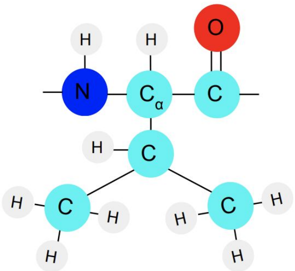

Isoleucine (Ile) I

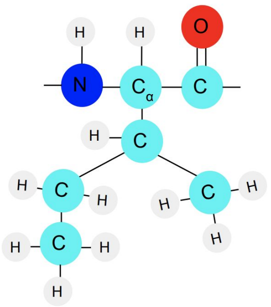

Leucine (Leu) L

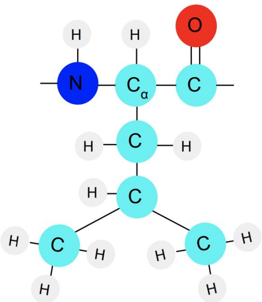

Methionine (Met) M

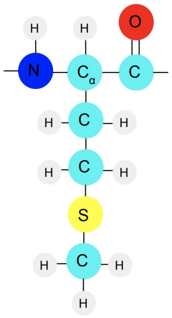

Phenylalanine (Phe) F

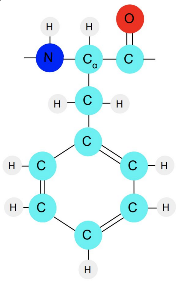

Tryptophan (Trp) W

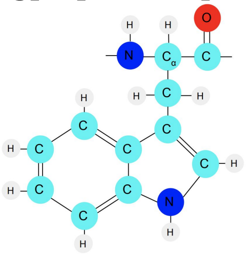

Cysteine (Cys) C

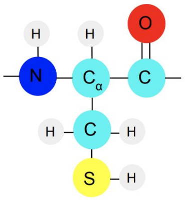

Proline (Pro) P

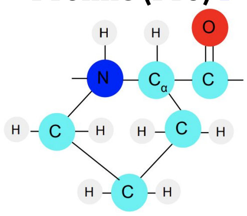

Supplement: Supplementary file 1 — ed4c00036_si_001.zip [file ed4c00036_si_001.zip › Kotsalidis_supporting_info_revisions/B-AMINO ACIDS IN PROTEINS HANDOUT.pdf]
