# Supplementary material for: EMMAs: Implementation and Assessment of a Suite of Cross-Disciplinary, Case-Based High School Activities to Explore Three-Dimensional Molecular Structure, Noncovalent Interactions, and Molecular Dynamics
Source: J Chem Educ. 2024 May 10;101(6):2436–47. doi: 10.1021/acs.jchemed.4c00036 (PMC11171454; doi:10.1021/acs.jchemed.4c00036)
Supplement: Supplementary file 1 — ed4c00036_si_001.zip [file ed4c00036_si_001.zip › Kotsalidis_supporting_info_revisions/02 - VMD-Ponatinib & Abl Kinase Chem 2.docx]

**Your Name:**

|  |
| --- |

**Partner Name:**

|  |
| --- |

**Introduction into Exploring Molecular Structure Using Visual Molecular Dynamics (VMD)**

**Exploring the drug Ponatinib**

In the prior activity, you read about Sandra, who has chronic myeloid leukemia (CML). You learned about a drug, imatinib, that can be used to treat CML by binding to a protein target. There are many drugs that can be used to treat CML and that can bind to this same protein. The specific way that drug molecules bind to the protein depends on the intermolecular forces (noncovalent interactions) between the molecules. Today, you are going to explore the structure of another drug used to treat CML, called ponatinib. The structure of the drug molecule determines how it will interact with the protein target.

1. **Loading the Drug Molecule into VMD**
2. Open the VMD software by clicking the VMD icon on your computer desktop
3. When you open the software, three windows will “pop up”: VMD Main window, OpenGL Display window, and VMD command window.
4. Locate the VMD Main window, click File → New Molecule.


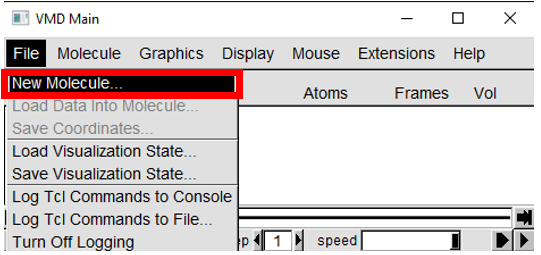

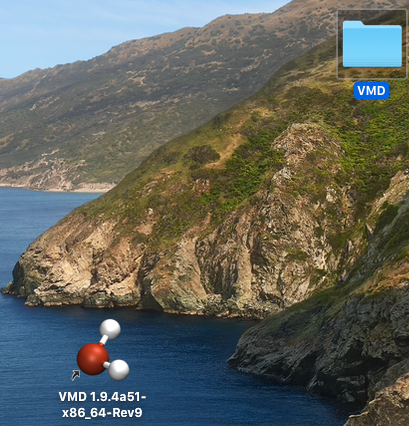


1. Click and Drag the **ponatinib_Sep2022.pdb** from the VMD folder into the Filename box, and click load.


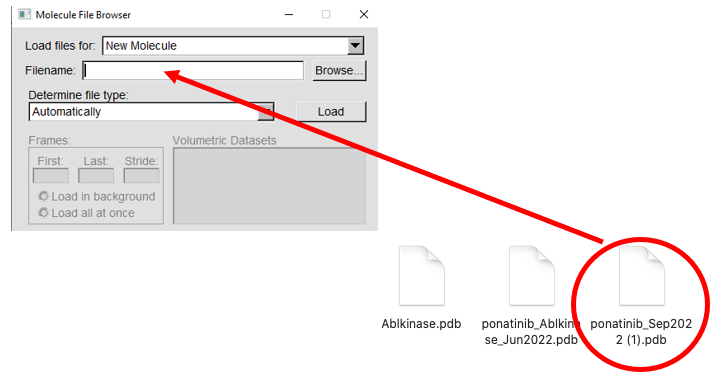


1. The drug molecule should be loaded into VMD and look like the picture below.


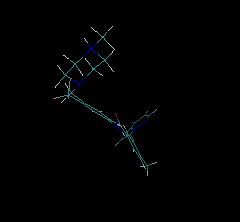


1. Now, the ponatinib molecule is shown in your OpenGL Display window. (You may close the Molecule File Browser window at any time.)

***IMPORTANT: Do not click on the green button to make the window larger. Instead put your mouse cursor on the lower right hand corner of the picture and pull.***

1. Click your mouse anywhere on the molecule and hold. Drag your mouse to move the molecule around the screen. In a few words, describe what you see on your screen. There is no “correct” answer to this question. Simply share a few observations.**(2 points)**

`

| **Delete this text and type your answer here.** |
| --- |

Each atom in the molecule has its own unique color. The colors of the atoms are summarized in the table below.

| **Atom Color** | **Atom Type** |
| --- | --- |
| Turquoise | Carbon |
| White | Hydrogen |
| Red | Oxygen |
| Blue | Nitrogen |
| Pink | Fluorine |

1. What one type of atom makes up most of the molecule? **(1 point)**

| **Delete this text and type your answer here.** |
| --- |

1. Do you think the atoms in the molecule are connected with covalent bonds, ionic bonds or intermolecular forces? Explain your answer. **(2 points)**

| **Delete this text and type your answer here.** |
| --- |

1. Identify one more structural feature of the molecule that interests you. **(1 point)**

| **Delete this text and type your answer here.** |
| --- |

1. **Viewing the Molecule**
2. In the VMD Main window select the Mouse menu. Explore switching the mouse mode between Rotation, Translation, and Scale. (You can also press the “T”, “S” and “R” keys on your keyboard to move between the three.)


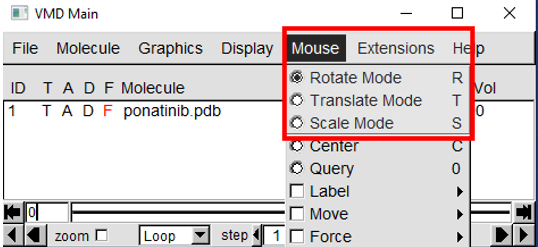


1. Describe how each of the mouse modes manipulates the molecule. **(3 points)**

| **(a) Translation (T) causes the molecule to move______________________.**  **(b) Rotation (R) causes the molecule to move________________________.**  **(c) Scale (S) causes the molecule to move___________________________.** |
| --- |

⭐**Tip: In the OpenGL Display you can reset the view by pressing the “=” key when you are in the window.**⭐

1. Now we are going to explore centering and rotating around a specific atom. In the VMD Main window click on Mouse → Center.


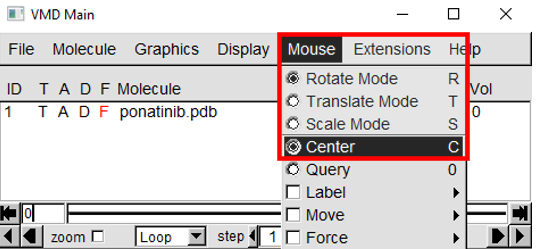


Pick one atom at one of the ends of the drug molecule and move the cross on top of it. Click your mouse. Then press “R” and rotate the molecule. Describe how your molecule moves. **(1 point)**

| **Delete this text and type your answer here.** |
| --- |

⭐**Tip: you can press the “C” key to also use this centering feature.**⭐

**You can find a summary of the most common VMD shortcuts and commands** [**here**](https://docs.google.com/document/d/1hrvWnPcHNHJkrqHxHY8J5Jtwa4spaki0rfVGo3ZDZsE/edit?usp=drive_link)**.**

1. You can customize a few of the Display settings in VMD. Tap the Display settings tab in the VMD Main window and scroll down to the setting you wish to change. Try changing between Perspective and Orthographic and see if you have a preference (It might help to press “R” and rotate after choosing each option to get a sense of how they differ). Click Display → Axes → Selection to remove or change the location of the (x-y-z) axes.
2. Take a moment to study the drug molecule once again. **Use your new skills with rotating, translating, scaling, and centering the molecule to answer the following questions. (4 points)**
3. How many ring-like structures can you find on the molecule?

| **Delete this text and type your answer here.** |
| --- |

1. Where do you notice that the white colored hydrogen atoms are located on the molecule?

| **Delete this text and type your answer here.** |
| --- |

1. How many red oxygen atoms do you see?

| **Delete this text and type your answer here.** |
| --- |

1. Determine the number of pink colored fluorine atoms on the molecule.

| **Delete this text and type your answer here.** |
| --- |


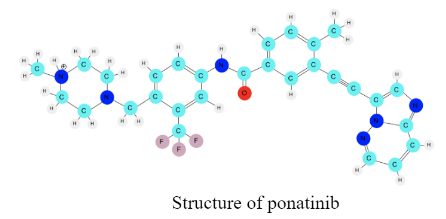


6. List **two** similarities and differences between the 2-D molecular structure of ponatinib shown above and the 3D molecular structure that you can manipulate on your screen. **(3 points)**

| **Similar** | **Different** |
| --- | --- |
| **Delete this text and type your answer here.** | **Delete this text and type your answer here.** |
| **Delete this text and type your answer here.** | **Delete this text and type your answer here.** |

1. **Drawing Methods**
2. In the VMD Main window select Graphics → Representations. A new window will pop up called the Graphical Representations window.

Notice the parameters that **you are currently working with**.

1. Coloring Method: Name
2. Drawing Method: Lines


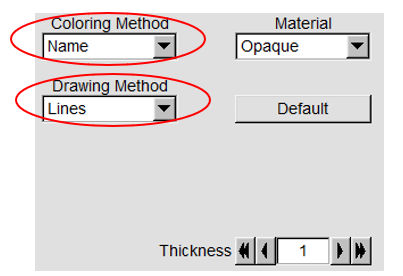


Next, let’s experiment with a few different **Drawing Methods**.

1. Input the following selections in the Graphical Representations window
   1. Coloring Method: Name
   2. Drawing Method: VDW

Describe what you see. **(1 point)**

| **Delete this text and type your answer here.** |
| --- |

1. Next try the following settings for graphical representations:
   1. Coloring Method: Name
   2. Drawing Method: CPK

Describe what you see. **(1 point)**

| **Delete this text and type your answer here.** |
| --- |

1. Next try the following settings for graphical representations:
   1. Coloring Method: Name
   2. Drawing Method: Licorice

Describe what you see. **(1 point)**

| **Delete this text and type your answer here.** |
| --- |

1. Now that we’ve seen a variety of different drawing methods let’s compare them! Match the following statements with the drawing method that you think would be most helpful in answering the question. More than one drawing method can be used for a single question.

**Drawing Methods**

| **VDW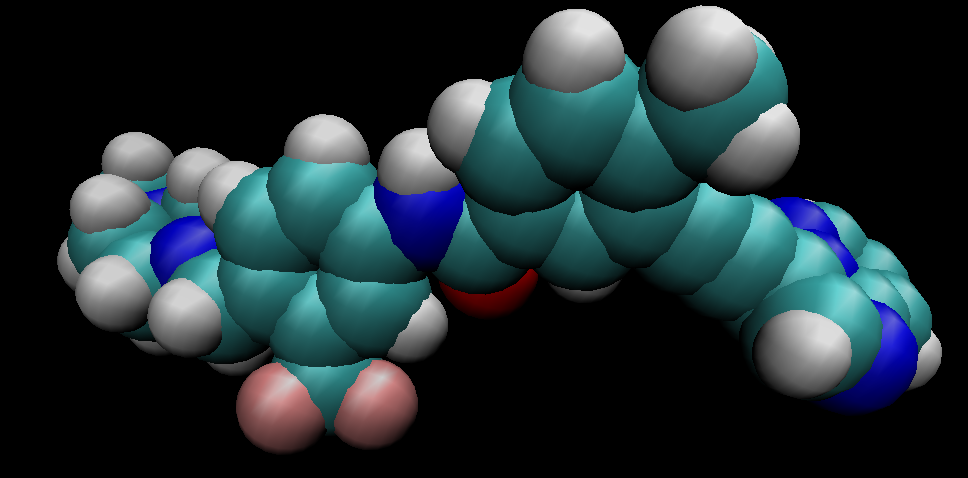** | **CPK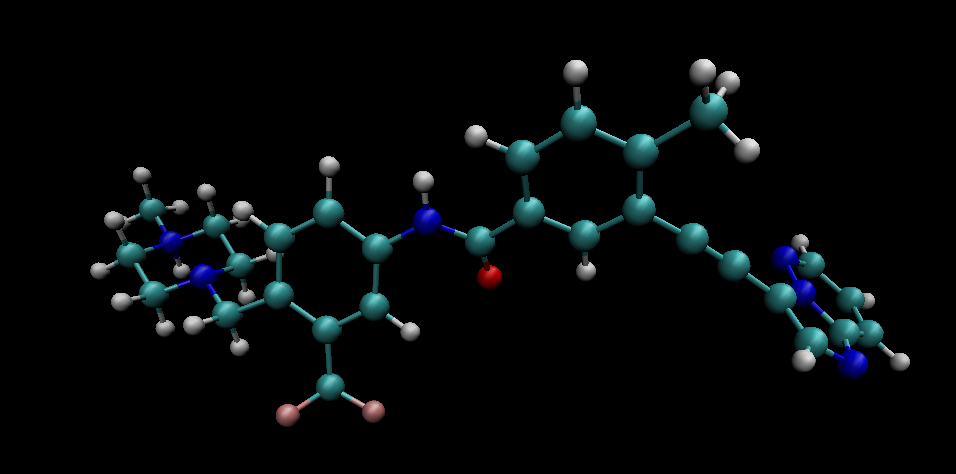** | **Lines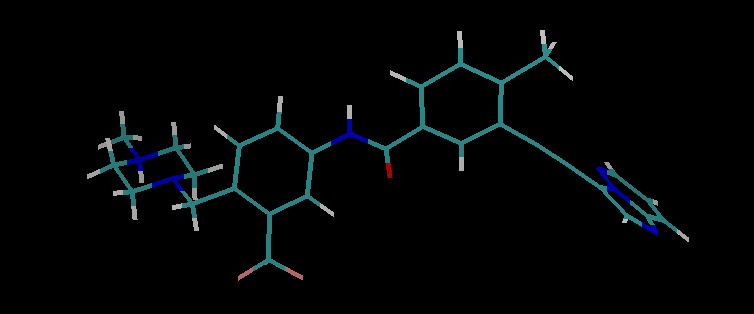** | **Licorice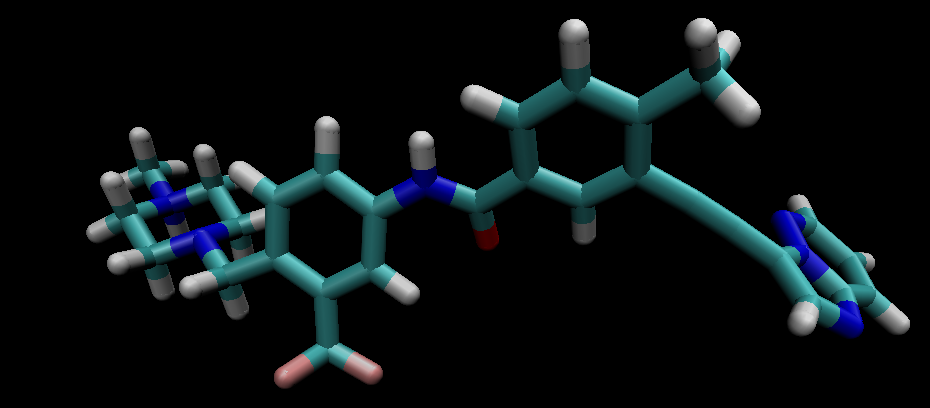** |
| --- | --- | --- | --- |

1. Which drawing method, in your opinion, is most helpful in identifying specific atoms – lines or CPK – and why? **(1 point)**

| **Delete this text and type your answer here.** |
| --- |

1. There are many interesting structural features in the ponatinib molecule. One such feature is a triple bond that looks like a longer extended line. Which drawing method, in your opinion, is most helpful in identifying the linear region in the ponatinib molecule – VDW or Licorice – and why? **(1 point)**

| **Delete this text and type your answer here.** |
| --- |

(c) Which drawing method, in your opinion, best helps you determine the angles around the atoms – Lines or VDW – and why? **(1 point)**

| **Delete this text and type your answer here.** |
| --- |

(d) Which drawing method, in your opinion, best helps you determine the amount of space taken up by the atoms in the molecule – Lines or VDW – and why? **(1 point)**

| **Delete this text and type your answer here.** |
| --- |

(e) Which drawing method, in your opinion, is most helpful in identifying the rings in the molecule – Lines or VDW – and why? **(1 point)**

| **Delete this text and type your answer here.** |
| --- |

(f) Do you think there is one “best” way to represent or draw molecules? Why or why not? **(1 point)**

| **Delete this text and type your answer here.** |
| --- |

1. **Determining Distances Between Atoms**
2. Input the following parameters into the Graphical Representations Window:
   1. Coloring Method: Name
   2. Drawing Method: CPK

⭐Remember that you can use the keys R, T, S, and C to manipulate the molecule.

R= rotate, T=translate, S=scale, and C=center ⭐

1. In the OpenGL Display window, press the “2” key. You will see a white cross appear on your screen.
2. Click on a pink fluorine atom. A label will appear next to the fluorine atom.
3. Fill in the blank for the label DRG285:F____. **(1 point)**

(DRG is an abbreviation for drug. There are three fluorine atoms, so each one has a different number 34-36.)

1. Now click on the red Oxygen atom. A label will appear next to the oxygen atom.
2. Fill in the blank for the label DRG285:O____. **(1 point)**
3. You will see a dashed white line between the two atoms with a white number. The number that pops up is the distance between the two atoms. VMD measures distance in Angstroms where

**1 Angstrom = 10^-10^ meters**. What is that distance? **(1 point)**

| **Delete this text and type your answer here.** |
| --- |

⭐You can hide labels and bonds by pressing “2” and clicking on the two atoms again.As another way to delete the labels and dashed white lines from your molecule, you can go to the VMD Main window and click on Graphics -> Labels. Select the atoms or bonds that you want to delete.⭐

1. Pick two other atoms to find the distance between and do so. Write the names of the atoms and their distance in the space below. **(2 points)**

| **Delete this text and type your answer here.** |
| --- |


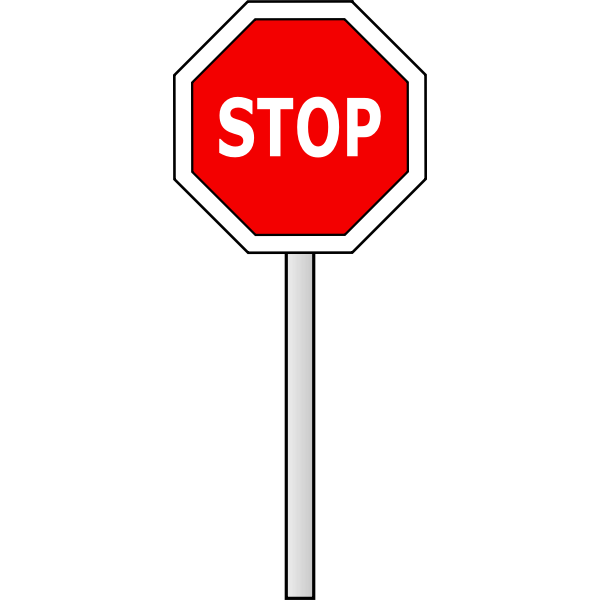


**Close VMD, and let your teacher know when you are finished with this part of the activity.**

[**OPTIONAL CHALLENGE FOR BONUS POINTS**](#bsjtmh2ig4kb)

**The Protein: Abl Kinase**

Thus far, you have learned that imatinib and ponatinib are two drugs that can treat chronic myeloid leukemia. They both bind to a kinase protein called the Bcr-Abl kinase. In addition, you have explored basic ways to use VMD to manipulate and analyze the structure of the drug molecule ponatinib. Next, you will explore the structure of the Abl kinase portion of this protein. Proteins are large complex molecules that are made up of many smaller units called amino acids. An amino acid in a protein is often called a “residue”. Proteins play a critical role in your body. They are required for regulation of the body’s tissues and organs, and they are required for structure and function.

1. **Loading the Protein: Abl Kinase into VMD**
2. In the VMD Main window, click File → New Molecule.


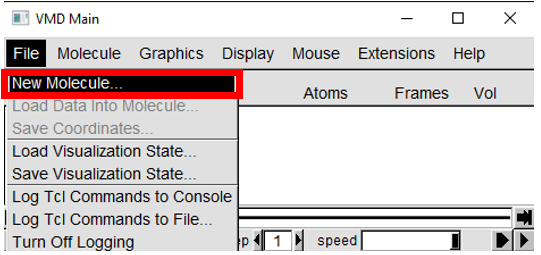


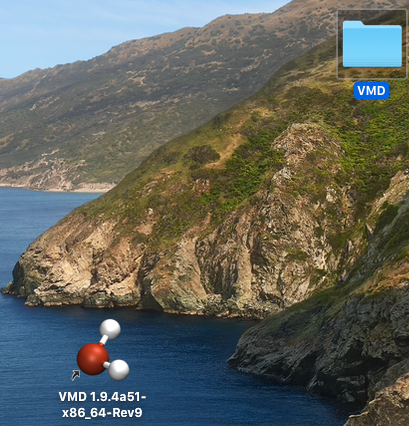


1. Locate the “**Ablkinase.pdb**” file in the VMD folder on your desktop.
2. Click and Drag the **Ablkinase.pdb** from the VMD folder into the Filename box, and click load.


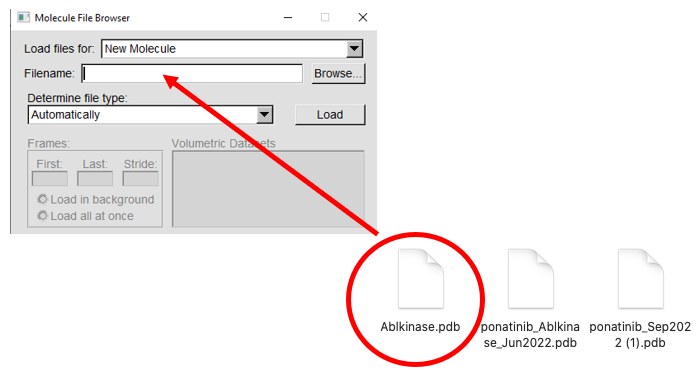


1. Now, the **Abl kinase protein** is shown in your **OpenGL Display window**. The OpenGL Display window is where you will view your molecules. (You may close the Molecule File Browser window at any time.)


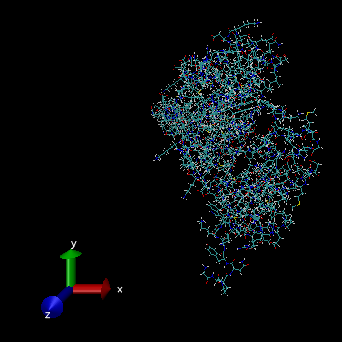


| **Color** | **Atom Type** |
| --- | --- |
| Turquoise | Carbon |
| White | Hydrogen |
| Red | Oxygen |
| Blue | Nitrogen |
| Yellow | Sulfur |

Use the VMD skills you have learned thus far to Zoom in and rotate the molecule (S-scale, R-rotate, T-translate, C-center).

1. List some of the different types of atoms you see in the protein. **(1 point)**

| **Delete this text and type your answer here.** |
| --- |

1. **Drawing Methods**

As you observed in the earlier exploration with the drug using VMD, many valuable methods exist for representing molecules.

In the VMD Main window select Graphics → Representations. The Graphics Display window will open.

Notice the parameters that **you are currently working with**.

Coloring Method: Name

Drawing Method: Lines
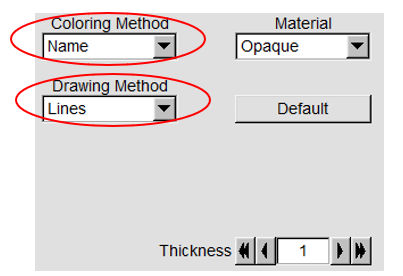


1. Change the drawing method to **VDW**.

There are six major Drawing Methods in VMD: VDW, CPK, Ribbons, Cartoon, Lines, and Licorice. Spend a few minutes exploring each method with the protein. Note that you have used VMD, CPK, Lines, and Licorice previously to look at the drug molecule ponatinib. Ribbons and Cartoon, however, are mainly used to look at proteins and other long polymers.

1. Experiment with a few of the different Drawing Methods and talk with your partner about which methods you like the best for the protein. List your favorite Drawing Methods in the space below. **(1 point)**

| **Delete this text and type your answer here.** |
| --- |

1. Now that you’ve explored a variety of different drawing methods let’s compare them! Match the following statements with the drawing method that you think would be most helpful in answering the question.

**Drawing Methods**

| **VDW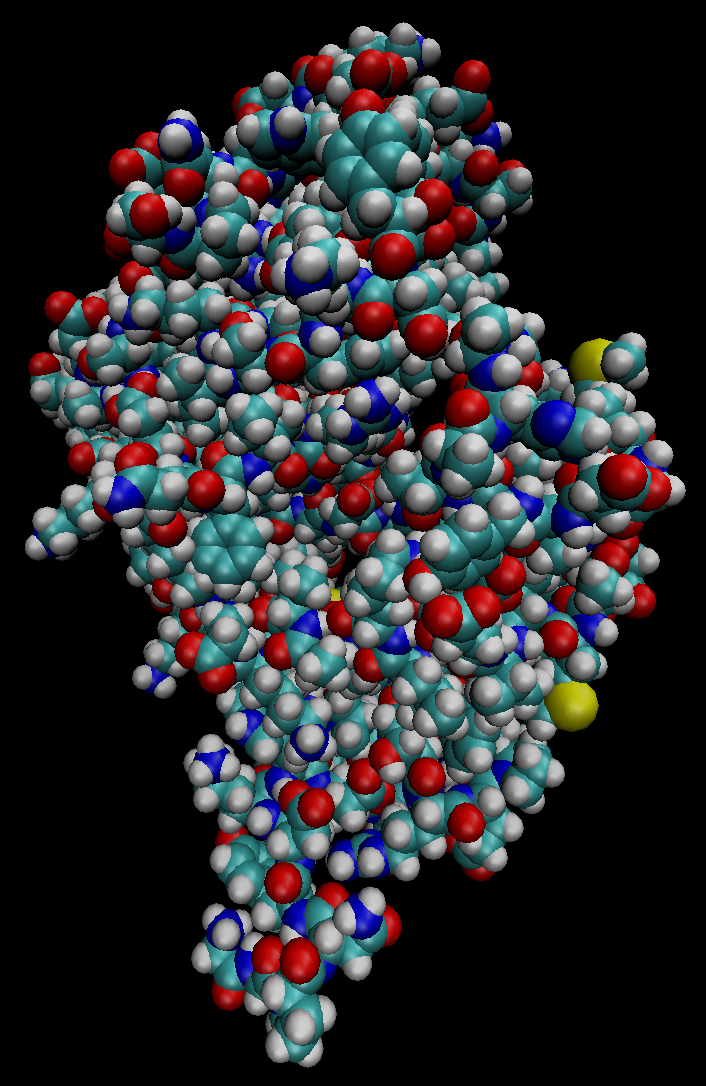** | **CPK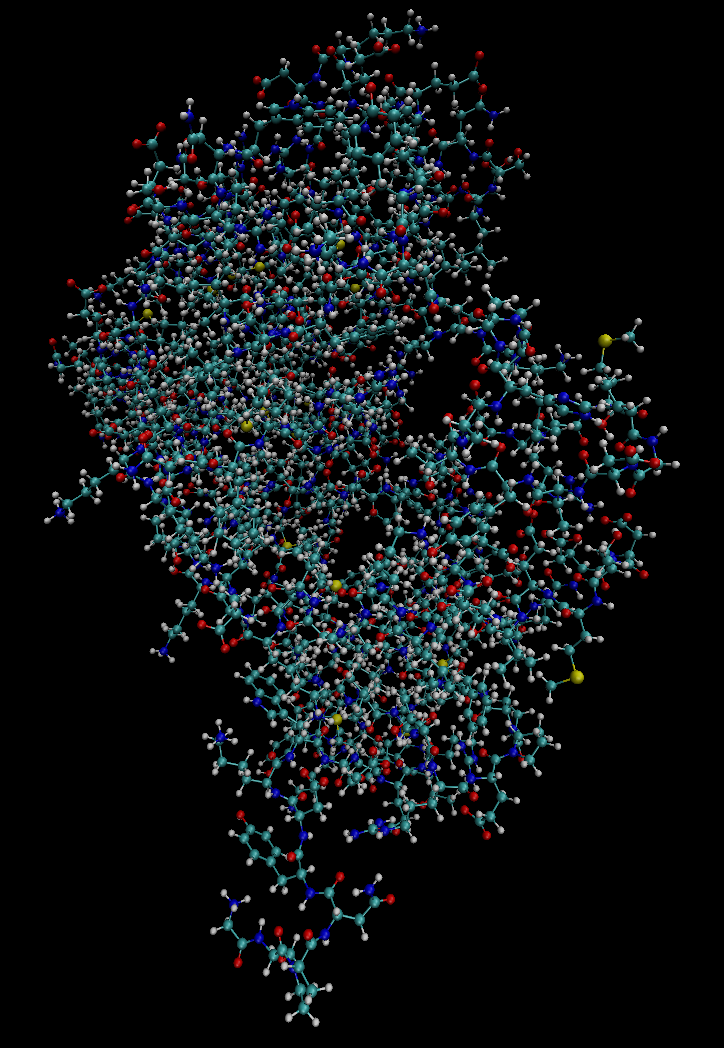** | **Ribbons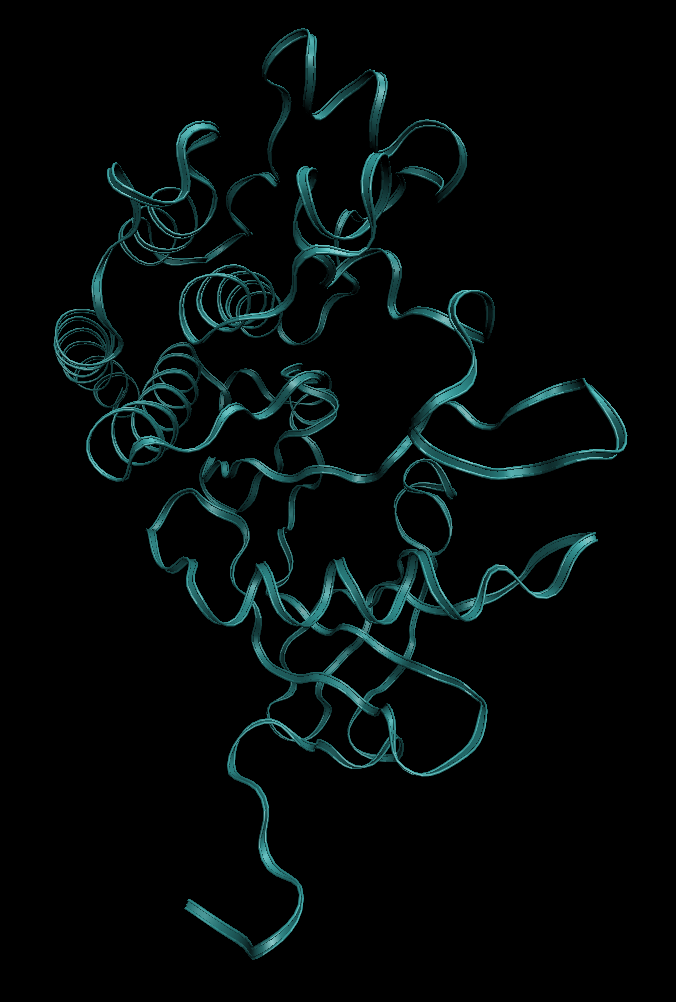** | **Cartoon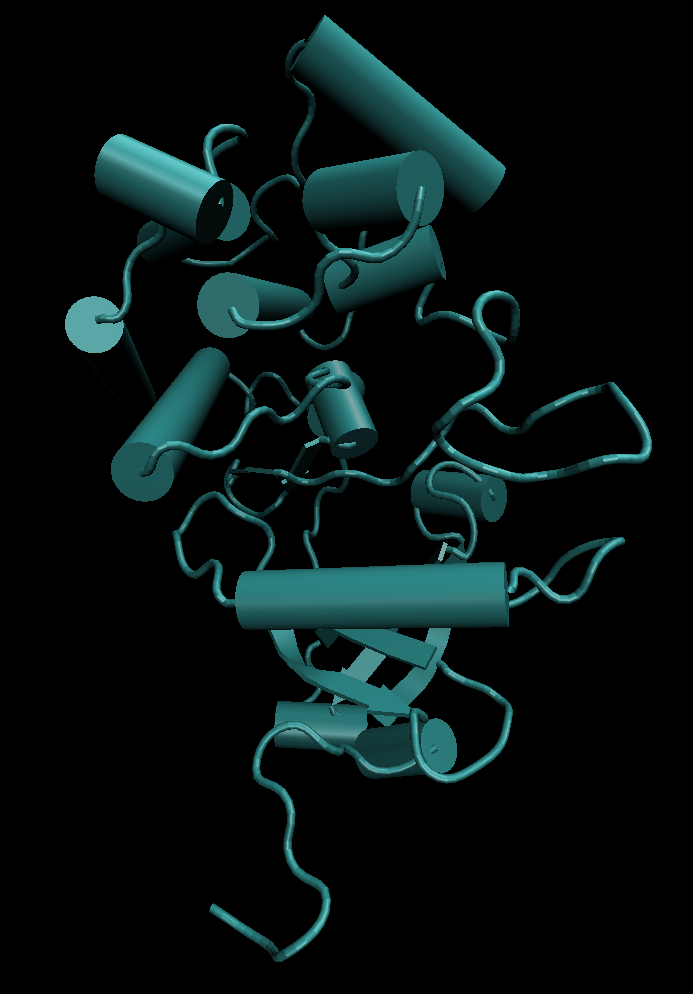** | **Licorice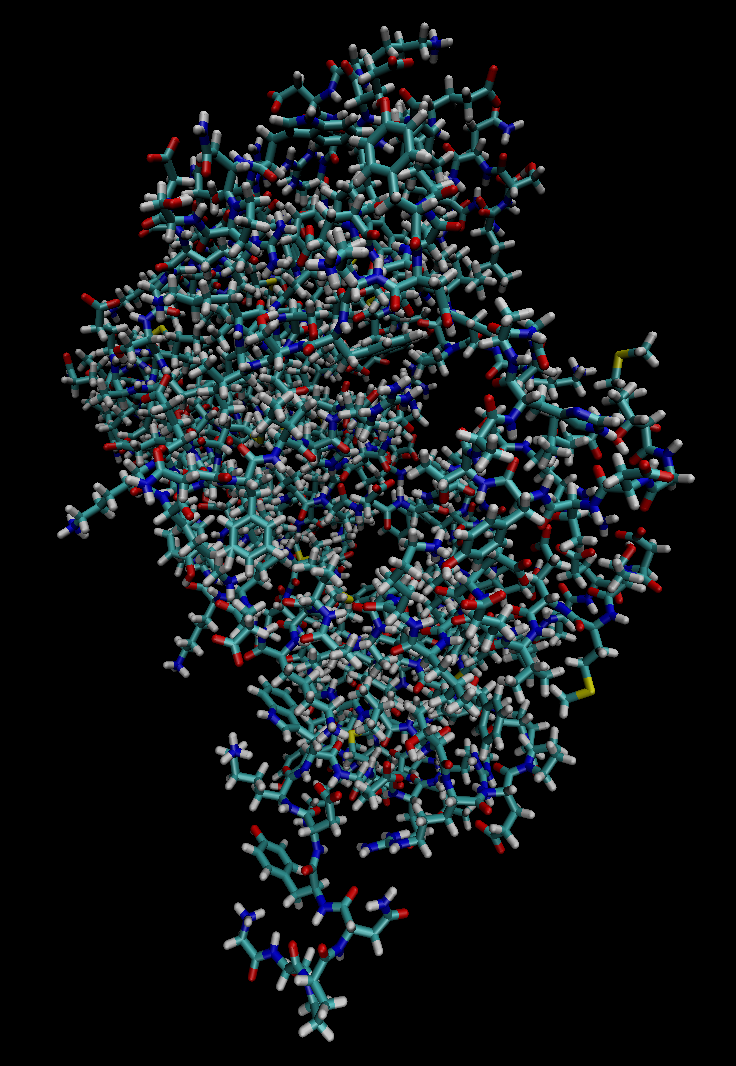** | **Lines**  **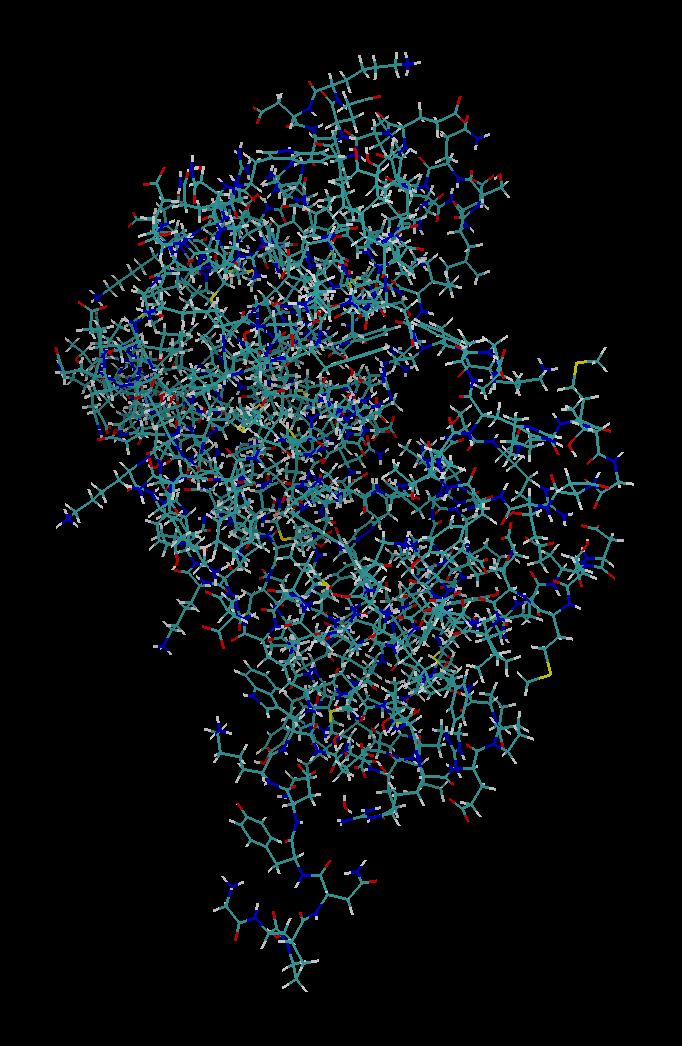** |
| --- | --- | --- | --- | --- | --- |

1. Which drawing method, in your opinion, is more helpful in identifying specific atoms – ribbons or CPK? **(1 point)**

| **Delete this text and type your answer here.** |
| --- |

(b) Which drawing method, in your opinion, better helps you determine the amount of space taken up by the protein – lines or VDW? Explain your answer**(2 points)**

| **Delete this text and type your answer here.** |
| --- |

(c) Which drawing method, in your opinion, better helps you determine the amino acids that make up the protein - cartoon or licorice ? Explain your answer. **(2 points)**

| **Delete this text and type your answer here.** |
| --- |

(d) Which drawing method, in your opinion, better helps you determine the bonds (how the atoms are connected) between the atoms – ribbons or lines ? Explain your answer. **(2 points)**

| **Delete this text and type your answer here.** |
| --- |

(e) Which drawing method, in your opinion, is less overwhelming to look at –cartoon or ribbons? Explain your answer. **(2 points)**

| **Delete this text and type your answer here.** |
| --- |

(f) Do you think there is one “best” way to represent or draw protein molecules? Explain your answer. **(2 points)**

| **Delete this text and type your answer here.** |
| --- |

(g) Which drawing method, in your opinion, better helps you identify alpha helices and beta sheets (also known as secondary structure) – VDW or cartoon? Explain your answer. **(2 points)**

| **Delete this text and type your answer here.** |
| --- |


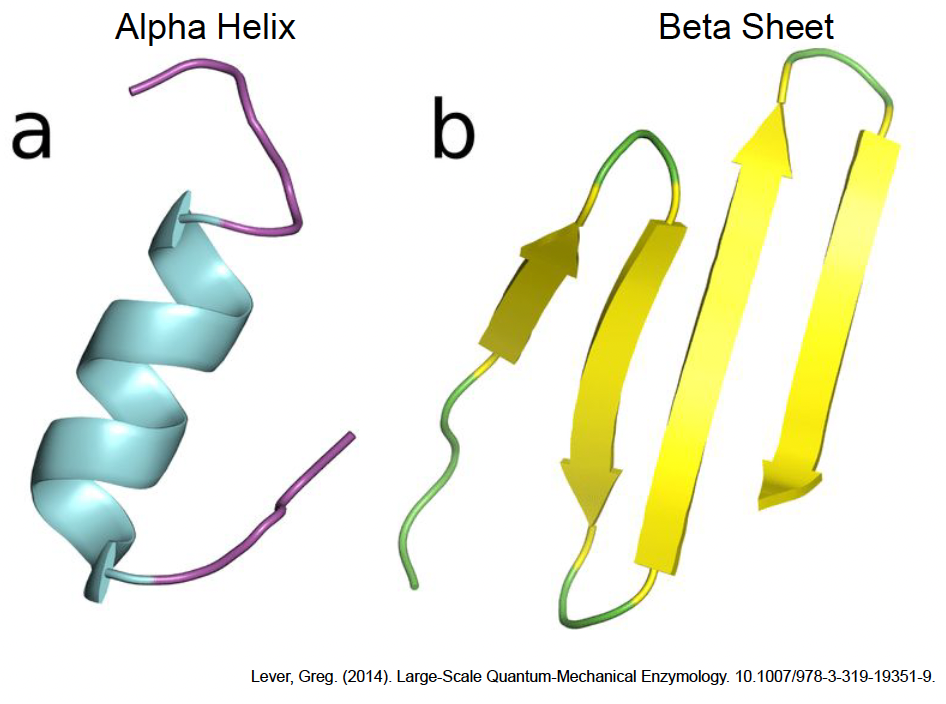


1. **Amino Acid Residue Selections**

As mentioned previously, an **amino acid** (small units that make up proteins) within a protein can also be called a “residue”. There are 20 amino acid residues used to make proteins. The order these amino acids string together determines the **primary sequence** of the protein, which in turn can determine how the protein **folds** into a 3D structure. The Abl kinase protein you are working with today has a unique 3D structure determined by the sequence of amino acid residues that make it up. The [Amino Acids Reference sheet](https://drive.google.com/file/d/1wGbIfO0yy8VcTLlB88T1_IGiannQvyrJ/view?usp=drive_link) shows the structural formulas for the amino acids.

In the **Graphical Representations** window enter the following:

1. Coloring Method: Name
2. Drawing Method: CPK
3. In the “Selected Atoms” box replace the word ALL with **resid 86**.


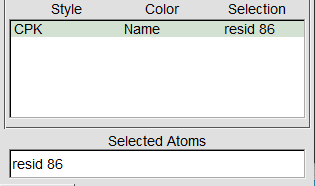


1. Click the Apply button. **Remember to press the = key to see the amino acid more clearly**

You’ve just selected an amino acid residue.

1. Use your [Amino Acids Reference sheet](https://drive.google.com/file/d/1wGbIfO0yy8VcTLlB88T1_IGiannQvyrJ/view?usp=drive_link) to write the name, three letter code, and single code for the amino acid you selected. **(1 point)**

| **Name:____________________________**  **Three Letter Code:______________**  **Single Letter Code:_____________** |
| --- |

1. Rotate, translate, and scale the 3D amino acid on your screen. In what ways does the 2D structure of the amino acid on your reference sheet compare to the 3D structure of amino acid on your VMD screen? List two similar and two different properties in the table below. **(2 points)**

| **Similarities** | **Differences** |
| --- | --- |
| **1. Delete this text and type your answer here.**  **2.Delete this text and type your answer here.** | **1.Delete this text and type your answer here.**  **2.Delete this text and type your answer here.** |

1. Now in the “Selected Atoms” box type “**resid 210**”. Click the Apply button and again center and zoom as needed using the [VMD commands](https://docs.google.com/document/d/1hrvWnPcHNHJkrqHxHY8J5Jtwa4spaki0rfVGo3ZDZsE/edit?usp=drive_link) you have learned.
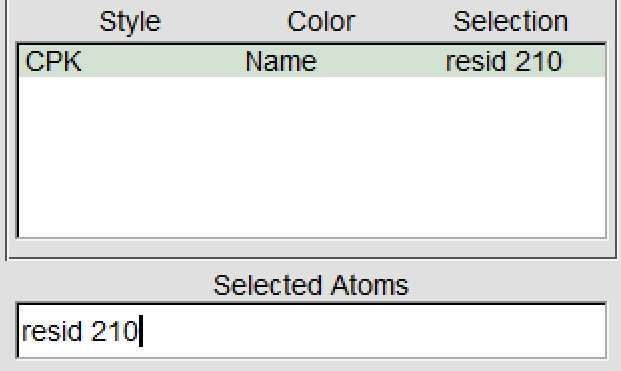


You’ve just selected another amino acid residue.

1. Use your [Amino Acids Reference sheet](https://drive.google.com/file/d/1wGbIfO0yy8VcTLlB88T1_IGiannQvyrJ/view?usp=drive_link) to write the name, three letter code, and single code for the new amino acid, resid 210, you selected. **(1 point)**

| **Name:____________________________**  **Three Letter Code:______________**  **Single Letter Code:_____________** |
| --- |

1. Rotate, translate, and scale the 3D **resid 210** on your screen. In what ways does the 2D structure of the amino acid on your reference sheet compare to the 3D structure of amino acid on your VMD screen? List two similar and two different properties in the table below. **(2 points)**

| **Similarities** | **Differences** |
| --- | --- |
| **1.Delete this text and type your answer here.**  **2.Delete this text and type your answer here.** | **1.Delete this text and type your answer here.**  **2.Delete this text and type your answer here.** |

1. **Protein Structure**

Proteins like the Abl kinase contain amino acids. Amino acids have a unique structure. Each amino acid shares a set of atoms that make up the amino acid **backbone**. The central carbon atom, also called the alpha carbon, has an atom or a group of atoms attached to it that varies among the amino acids (These groups are called side chains). When strung together, these amino acids make up a protein.

Here’s what a string of amino acids looks like:

**
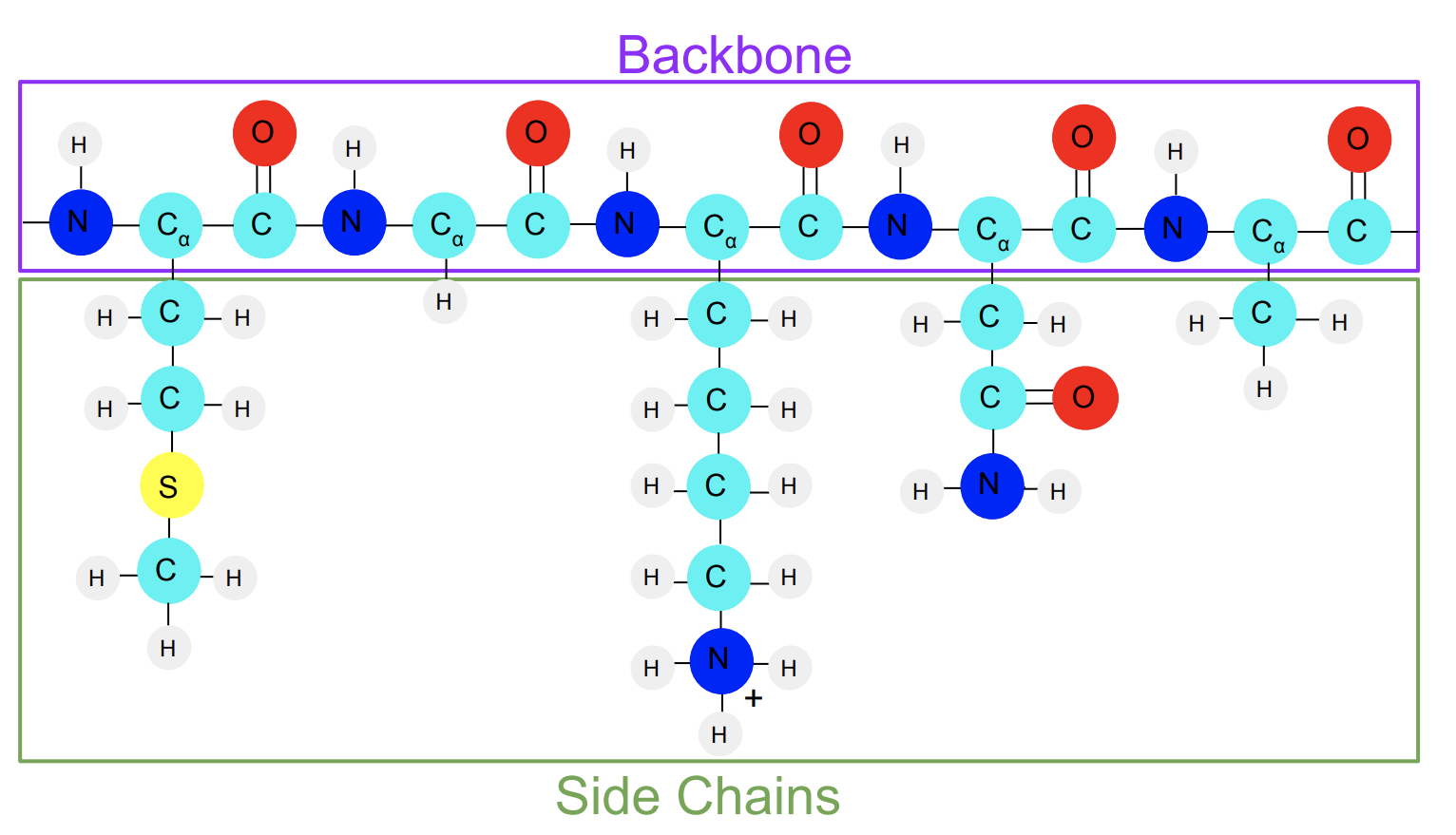
**

Next, let’s look at the backbone of the Abl kinase protein.

1. In the “Selected Atoms” box type “backbone” and press “enter”.


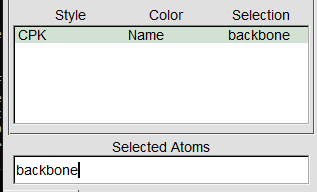


1. Describe what you notice about the backbone of the Abl kinase protein. **(1 point)**

| **Delete this text and type your answer here.** |
| --- |

1. What types of atoms do you see in the backbone? List the types of atoms in the space below.

**(1 point)**

| **Delete this text and type your answer here.** |
| --- |

1. Based on what you see in the representation, why do you think it is called the “backbone” of Abl Kinase? **(1 point)**

| **Delete this text and type your answer here.** |
| --- |

**END**

**Congratulations! You have just finished manipulating, analyzing, and exploring your first molecules using VMD. See your teacher for next steps.**

**Be sure to log out of your Gmail Account.**


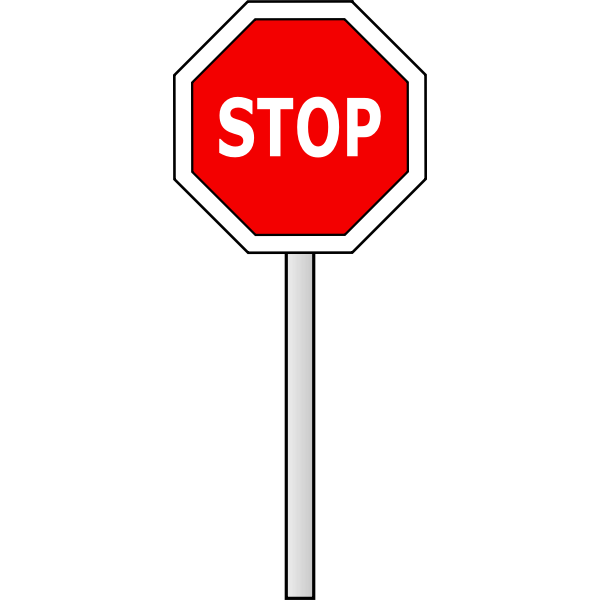


**CHALLENGE**


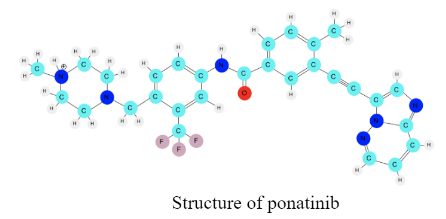


Try this next one if you want to earn **bonus points**. If you have trouble then skip it and come back later.

1.Find one central atom with each of the following molecular geometries (Remember that you can find the label of an atom by clicking on it and looking at the letters and numbers after the colon (e.g., “C31” or “H40”). Again, the “DRG” before the colon stands for “DRUG” and refers to what the molecule has been named in this case. You can remove a label if you don’t need it anymore by clicking on that atom again.)

Indicate the labels of the atoms you find in the spaces below:

Tetrahedral:

| **Delete this text and type your answer here.** |
| --- |

Trigonal planar:

| **Delete this text and type your answer here.** |
| --- |

Trigonal pyramidal:

| **Delete this text and type your answer here.** |
| --- |

Bent:

| **Delete this text and type your answer here.** |
| --- |

Linear:

| **Delete this text and type your answer here.** |
| --- |
