# Supplementary material for: EMMAs: Implementation and Assessment of a Suite of Cross-Disciplinary, Case-Based High School Activities to Explore Three-Dimensional Molecular Structure, Noncovalent Interactions, and Molecular Dynamics
Source: J Chem Educ. 2024 May 10;101(6):2436–47. doi: 10.1021/acs.jchemed.4c00036 (PMC11171454; doi:10.1021/acs.jchemed.4c00036)
Supplement: Supplementary file 1 — ed4c00036_si_001.zip [file ed4c00036_si_001.zip › Kotsalidis_supporting_info_revisions/06 - Molecular Dynamics EdPuzzle Questions.docx]

[Video 1](https://edpuzzle.com/media/621a7f39a7d9e9430666dfb1)

- 0:59- What biological processes is the Abl kinase involved in? (Open response)
- 1:24- What are the names of some of the drugs that have been developed to treat CML? Ponatinib, aspirin, imatinib (Multiple choice)
- 1:58- Why is it not ideal to just look at the static structure of a protein when studying protein-drug interactions? (Open response)
- 3:26- In your own words, explain what an MD simulation is. (Open response)

[Video 2](https://edpuzzle.com/media/621a83a0933c1242edc3fe8e)

- 0:47- Which of the following is an ion? Choose all that apply. K+, BF_3_, SO_3_^2–^, H_2_O (Multiple choice)
- 1:38- What does r represent in the equation? Charge of the ions, Force experienced by the ions, Distance between the ions (Multiple choice)
- 2:52- What are some of the most important steps when creating an MD Simulation? Determining the distance between the ions or atoms, Finding the mass of the ions or atoms, Calculating the forces experienced by the ions or atoms, Taking a small timestep (Multiple choice)
- 4:06- Does the force experienced by the ions change over the timestep? If so, how does it change? (Open response)
- 5:40- What disciplines of STEM are used when creating MD Simulations? Choose all that apply. Physics, Math, Chemistry, Biology, Computer science (Multiple choice)
