# Supplementary material for: EMMAs: Implementation and Assessment of a Suite of Cross-Disciplinary, Case-Based High School Activities to Explore Three-Dimensional Molecular Structure, Noncovalent Interactions, and Molecular Dynamics
Source: J Chem Educ. 2024 May 10;101(6):2436–47. doi: 10.1021/acs.jchemed.4c00036 (PMC11171454; doi:10.1021/acs.jchemed.4c00036)
Supplement: Supplementary file 1 — ed4c00036_si_001.zip [file ed4c00036_si_001.zip › Kotsalidis_supporting_info_revisions/07 - Investigation of MD Simulations Activity.docx]

**Investigation of Molecular Dynamics Simulations**

**Our System**

As we have learned, chronic myeloid leukemia (CML) is a cancer of the myeloid cells, which are cells that make red blood cells, platelets and most types of white blood cells.^^[[1]](#footnote-0)^^ The disease is characterized by a reciprocal translocation where the formation of the Philadelphia chromosome coding for BCR/ABL fusion protein results. This protein is constitutively active, meaning it is always on.


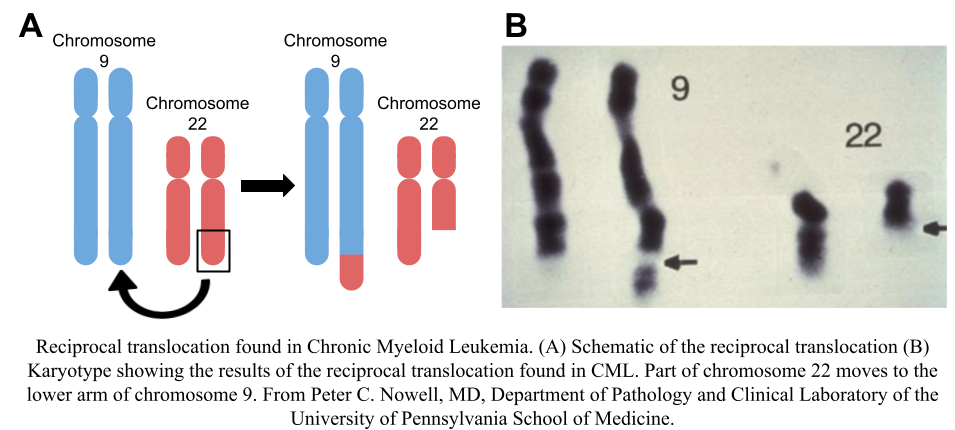


Kinases, like the Bcr-Abl kinase we have been working with, bind ATP in a binding pocket and catalyze the phosphorylation of different molecules. Phosphorylation is the process by which a phosphate molecule is transferred to another molecule.


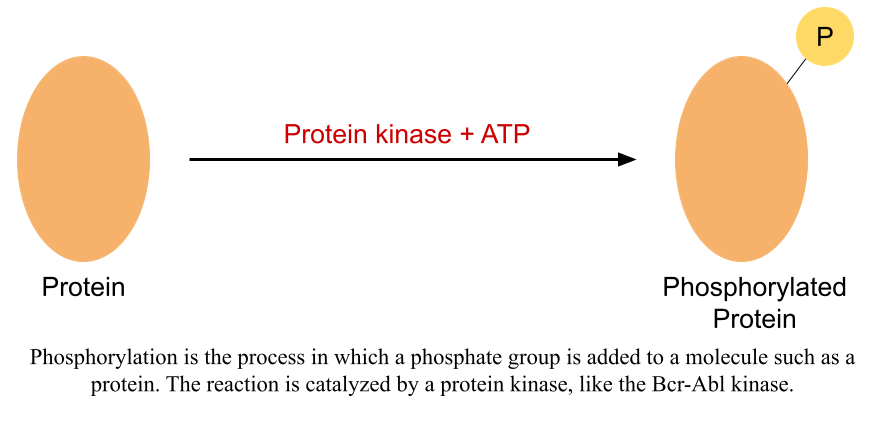


Kinases exist in both an active and inactive state. In the active state they can do their job of phosphorylation. The Bcr-Abl kinase has an activation loop that switches conformation from inactive to active leading to phosphorylation and activation of signaling molecules leading to cell proliferation and thus cancer.


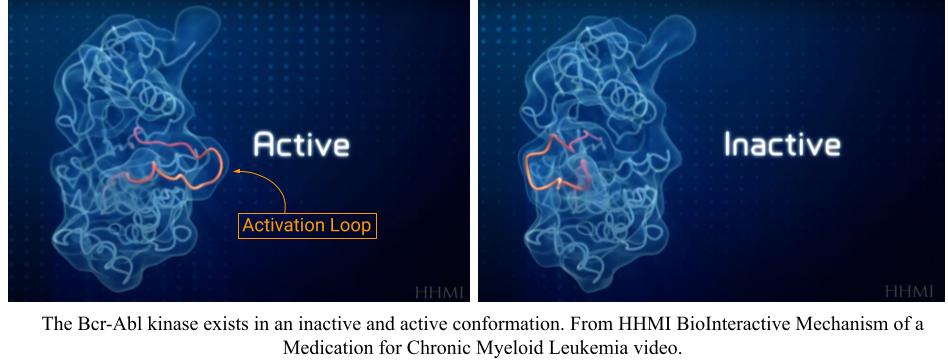


Kinases play an important role in disease such as CML and are actually the second largest drug target family.^^[[2]](#footnote-1)^^ Different generations of drugs have been developed to treat CML, all of which are called tyrosine kinase inhibitors (TKIs). Currently, TKIs are the only treatment option for CML patients.^^[[3]](#footnote-2)^^ These molecules are called “inhibitors” because they bind in the same pocket that ATP normally binds to and thus inhibits the protein’s function.

The first drug that was developed, and the one we will be looking at today, is called imatinib, and is also known by the brand name, Gleevec. It binds to the binding site of the kinase, locking it in the inactive conformation. When imatinib binds, it forms different intermolecular interactions which we will be investigating today.


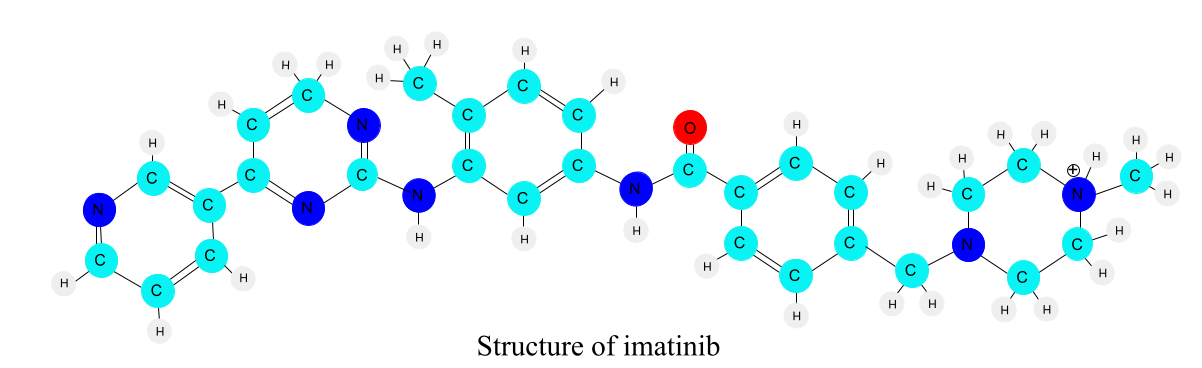


The introduction of imatinib for treating CML revolutionized cancer therapy. 12 months post imatinib introduction, 96% of patients had complete hematologic response (return to normal blood count) and 85% had complete cytogenetic response (absence of the Philadelphia chromosome in blood cells).^^[[4]](#footnote-3)^^

Let’s look at the following graph depicting the white blood cell counts in six patients (each shown in a different color) with CML who have been treated with imatinib (STI571). Each patient received 500 mg of imatinib per day for 150 days. The dotted line represents the upper limit of the normal white blood cell count range.


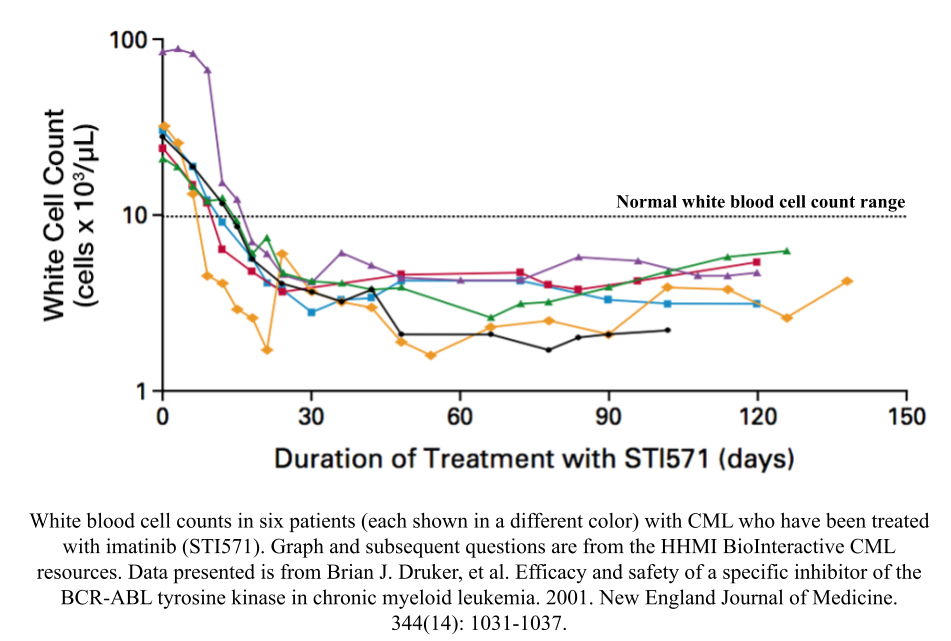


1. Describe the overall trend that you see in this graph.

|  |
| --- |

1. What do the initial values at time zero indicate about the health of the patients? Use the dotted line as a reference.

|  |
| --- |

1. How do the trends of the lines from 0 to 30 days compare to the trends of the lines from 30 days to 150 days? Comparing these trends, what claim could you make about the patients’ response to the drug?

|  |
| --- |

1. Why does the graph show six patients over 150 days and not just one patient? Or all 83 patients?

|  |
| --- |

1. Compare the general range of cell counts for all six patients at 0 days, 30 days, 60 days, 90 days, and 120 days. What does this tell you about individual responses to treatment?

|  |
| --- |

1. Would you be willing to approve this drug for treatment of other patients based on the results for six patients in this graph alone? What additional evidence would you want to see?

|  |
| --- |

Despite imatinib’s ability to treat many patients, other patients who had already entered advanced-stage CML and tried treatment with imatinib did not have this positive response. In fact many of the patients relapsed.^^[[5]](#footnote-4)^^ Mutations, or changes in the DNA sequence, were detected in 90% of these patients.^^[[6]](#footnote-5)^^ One of the main mutations detected in these patients was a threonine to isoleucine mutation at residue 315. If we remember back to our amino acid properties, we recognize that this is a change from a polar to a nonpolar amino acid. We will be looking at this mutated Abl kinase protein in this activity as well.


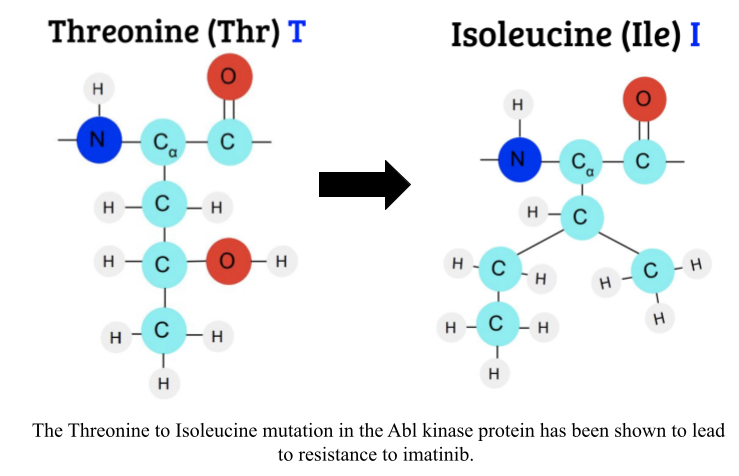


**MD Simulations**

Thus far we have been working with static structures in VMD. Static structures are an average structure at a single point in time and thus they don’t necessarily represent the realistic and dynamic nature of molecules. Not only this, but we can’t model and analyze conformational changes and dynamic intermolecular interactions by looking at static structures. That’s where MD simulations come in! We have learned about MD simulations and how they are computer simulation methods for analyzing the physical movements of atoms and molecules.

In this activity we will be comparing the structures of the wild type Abl kinase and its interaction with imatinib as well as the mutated T315I mutated kinase. To get started, download the following two MD simulations: [wild type](https://drive.google.com/file/d/1BRfYYFXMqph3Fr8a3dowIGOUlBwOzkTK/view?usp=drive_link) and [mutant.](https://drive.google.com/file/d/1VX1loruI_5BjXYnMDn4xejVQJtkSbqVP/view?usp=drive_link)

**Activity**

In the following activity, we will be studying MD Simulations of the wild type and mutant Abl kinase with the drug imatinib. We will first take a look at the hydrogen bonds between the protein and drug and then move on to studying distances between the protein and drug.

**Hydrogen Bond Analysis**

Intermolecular forces are forces that mediate interactions between molecules. These include London Dispersion forces, dipole-dipole interactions and hydrogen bonds. In this activity we will be focusing on hydrogen bonds. Hydrogen bonds are a special type of dipole-dipole interaction where a hydrogen atom covalently bonded to an electronegative atom interacts with a fluorine, oxygen, or nitrogen atom. We will be investigating the hydrogen bonds between the Abl kinase and imatinib molecules. Our goal is to determine whether the number of hydrogen bonds differ between the wild type and mutant proteins.

Let’s start by analyzing the hydrogen bonds between the **wild type protein** and imatinib.

1. Download the [wild type MD simulation](https://drive.google.com/file/d/1BRfYYFXMqph3Fr8a3dowIGOUlBwOzkTK/view?usp=drive_link).
2. Open VMD by clicking on the software.
3. Once in VMD, load in the MD simulation of the wild type protein using the following steps.
   1. In the VMD Main window click File → New Molecule
   2. Locate the “wildtype.pdb” file on your desktop. Drag the file into the Filename box and click Load.
4. The MD simulation will immediately start playing. At this moment in time, the speed of our simulation is very fast so we are going to want to slow it down. In the “VMD Main” window slide the slider for speed all the way to the left.


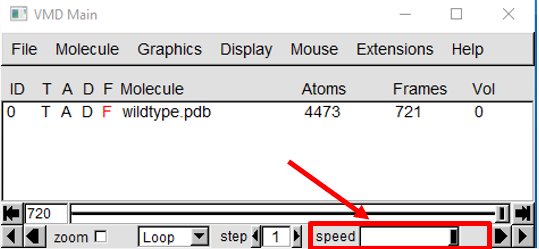


Now press the play button in the “VMD Main” window. Your simulation will start playing.

What are a few things you notice about the simulation?

|  |
| --- |

1. What we are observing is an MD Simulation. We are observing the Abl kinase and imatinib molecules moving around over time as we simulate how they would move around in a biological system.
2. To study the hydrogen bonds between the protein and drug, VMD has a built-in analysis tool.
3. To obtain a graph of the number of hydrogen bonds over time use the following steps:
   1. In the VMD Main window, click on Extensions→ Analysis→ Hydrogen Bonds (it is the 7th option on the list)


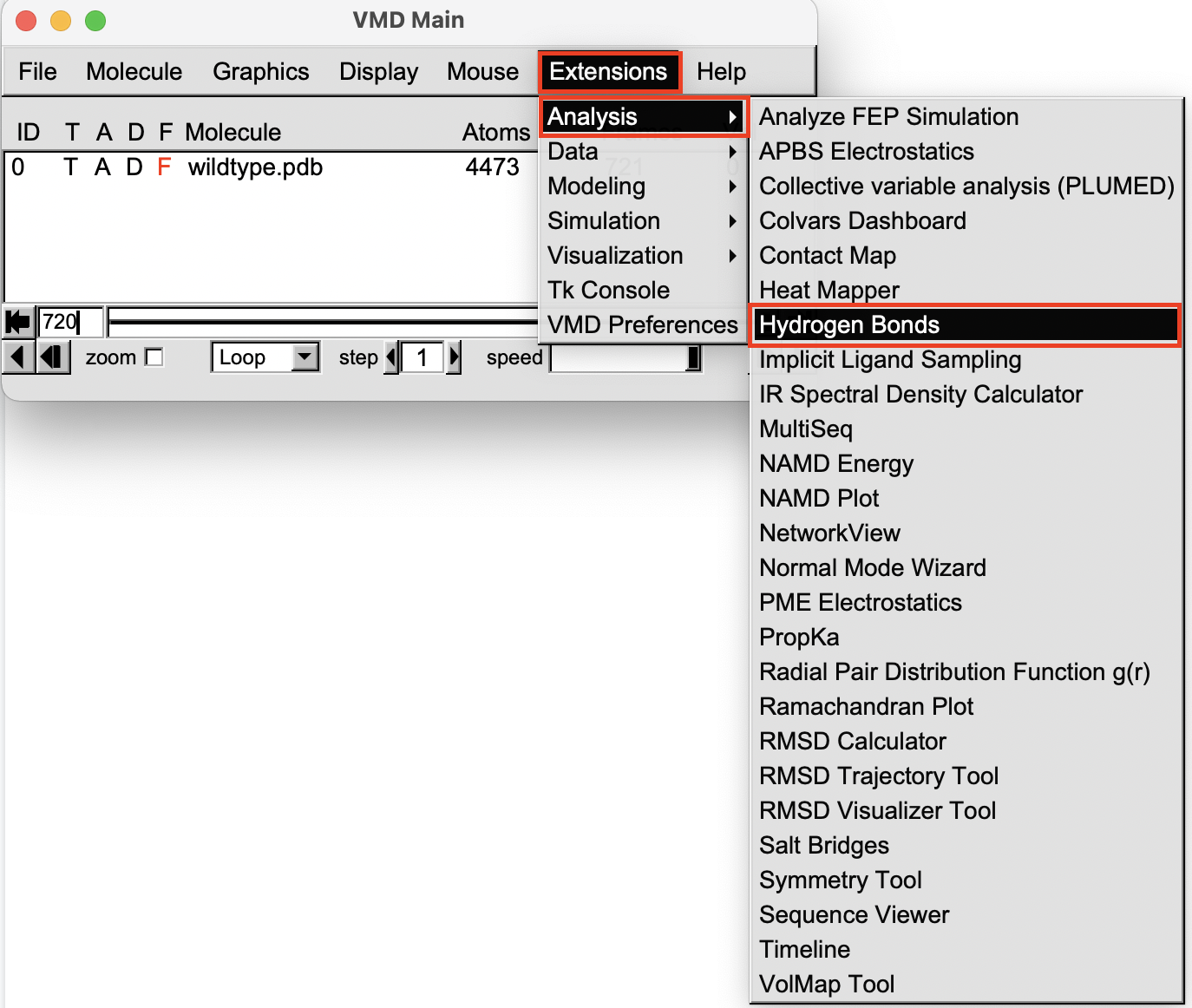


1. A window will pop up that looks like the following


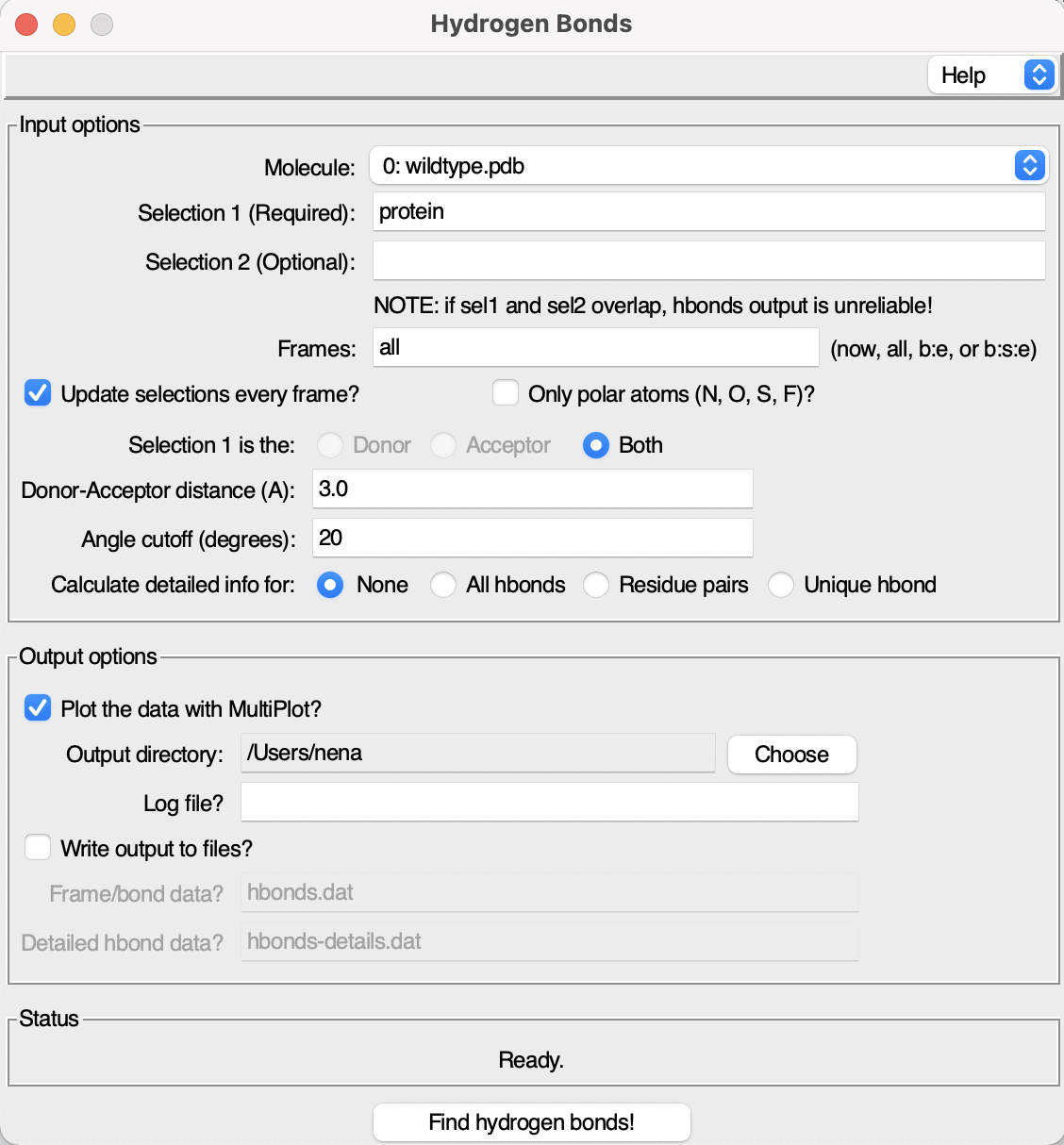


1. To figure out the number of hydrogen bonds between the protein and drug, we have to tell the program which two molecules to look at. Our Abl kinase is called “chain A” and the imatinib is called “chain B”.
2. We want to study the number of hydrogen bonds between the Abl kinase and imatinib so our two selections have to reflect that. In “Selection 1” type “chain A”. In “Selection 2” type “chain B”.
3. We want to study all the frames of our simulation so the “Frames” field should say “all”.
4. “Update selections every frame?” and “Only polar atoms (N, O, S, F)” should be checked.
5. For “Selection 1 is the:” “Both” should be clicked as “selection 1”, the protein, can act as both a hydrogen bond donor *and* acceptor.
6. The “Donor-Acceptor distance (A)” should be 3.5, where 3.5 has units of Angstroms. This is the average length of a hydrogen bond. Thus, we are using it as a “cut-off distance” where anything longer than 3.5 Angstroms won’t be counted as a hydrogen bond.
7. The “Angle cutoff (degrees)” should be 30.
8. For “Calculate detailed info for:”, “None” should be clicked.
9. You should then check “Plot the data with MultiPlot?” since we want to get a graph as the output.
10. For “Output directory” click “Choose” and then “Desktop” and then “Choose” again to lock in the place.
11. “Log File?” can stay blank and “Write output files” should also stay unchecked.
12. Your screen should look like this:


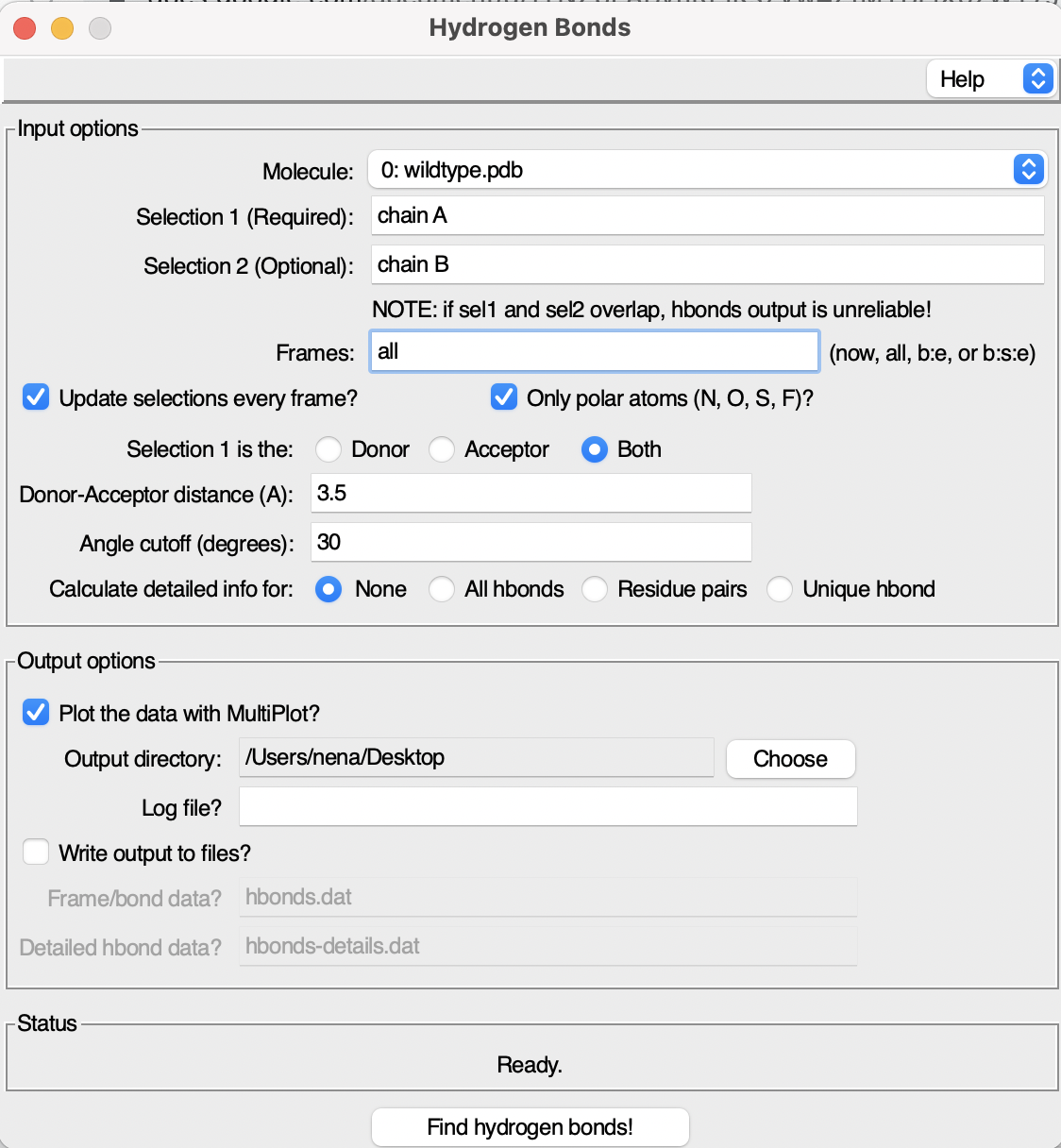


1. We are now ready to find the number of hydrogen bonds! Click “Find hydrogen bonds!”
2. It may take a few seconds, but a graph should pop up that shows all the hydrogen bonds formed in the simulation and their frequency. Paste a screenshot of your graph below.

|  |
| --- |

1. The y-axis shows the number of hydrogen bonds (No. Bonds) between the protein and drug, while the x-axis shows the “frames” or “snapshots” during which these hydrogen bonds were measured.
2. What is the maximum number of hydrogen bonds formed between the wild type Abl kinase protein and imatinib?________
3. What is something interesting you observe about the graph?________________________

Let’s now analyze the hydrogen bonds between the **mutant protein** and imatinib. As a reminder, this mutant has a threonine to isoleucine mutation at residue 315.

1. Close the window with your hydrogen bond graph as well as the “Hydrogen Bonds” window.
2. In the VMD Main window, click on Graphics→ Representations. A Graphical Representations window will appear. This window may be familiar as we used it in our other activities.
3. Double click on the word “all” under the “Selections”. It should turn red and your molecule should disappear in the OpenGL Display window.


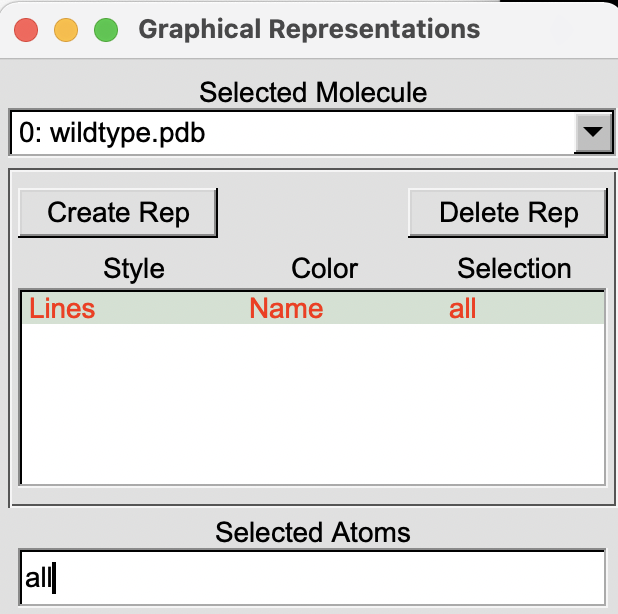


1. We are now ready to load in our mutant kinase and imatinib MD simulation. Download the [mutant MD simulation](https://drive.google.com/file/d/1VX1loruI_5BjXYnMDn4xejVQJtkSbqVP/view?usp=drive_link).
   1. Open VMD by clicking on the software.
   2. Once in VMD, load in the MD simulation of the mutant protein using the following steps.
      1. In the VMD Main window click File → New Molecule
      2. Locate the “mutant.pdb” file on your desktop. Drag the file into the Filename box and click Load.
2. The simulation of the mutant protein and drug will start playing.
3. We will again obtain a graph of the hydrogen bonds between the protein kinase and drug, but this time we are looking at the mutant protein.
4. In the VMD Main window, click on Extensions→ Analysis→ Hydrogen Bonds
5. Under “Molecule” click the dropdown menu and click “mutant.pdb”.
6. Replicate all the settings you had for the wild type protein. Your screen should look like this, with the following inputs and checked boxes:


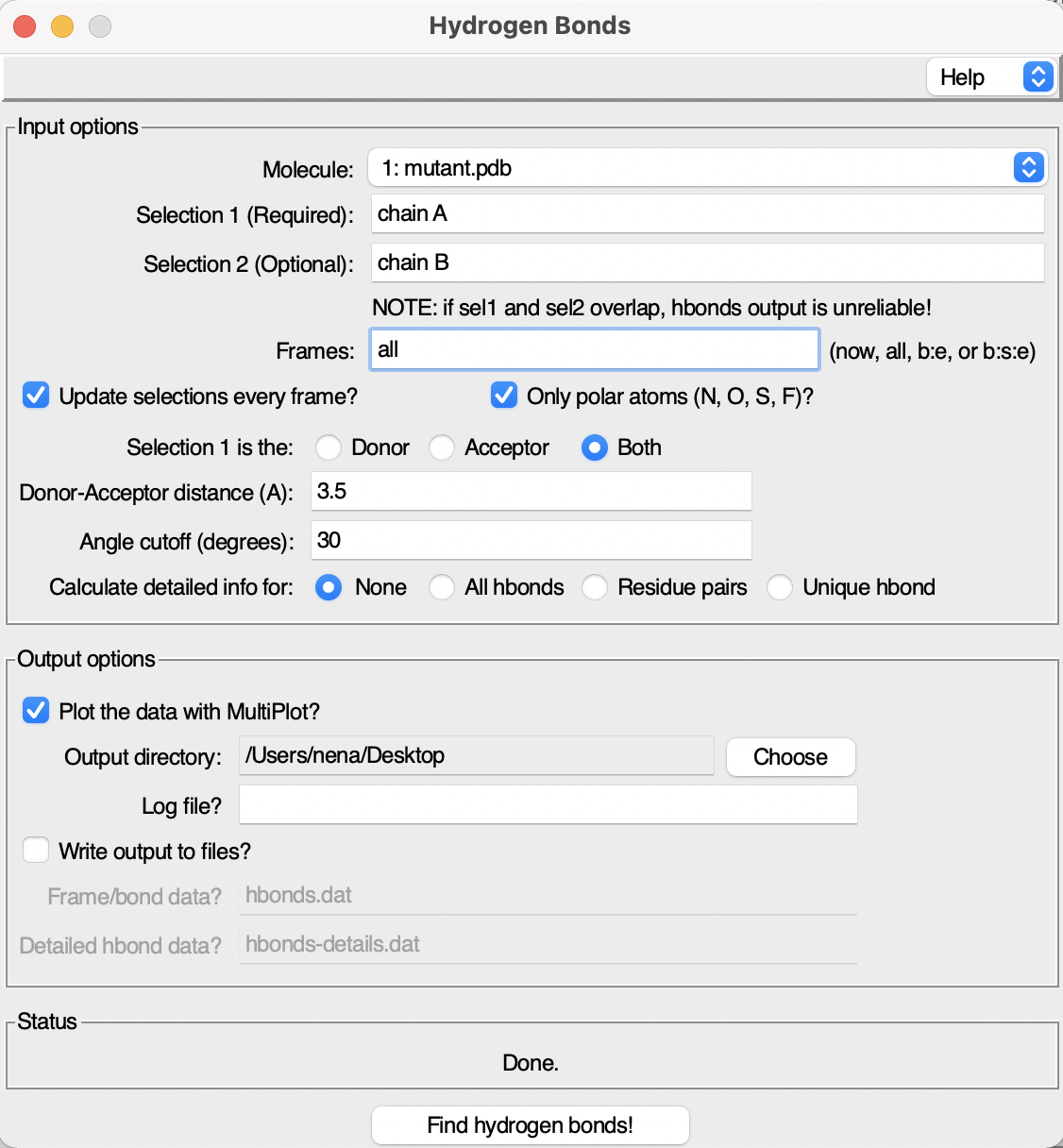


1. Click “Find hydrogen bonds!”
2. It may take a few seconds, but a graph should pop up that shows all the hydrogen bonds formed in the simulation and their frequency. Paste a screenshot of your graph below.

|  |
| --- |

1. Once again, the y-axis shows the number of hydrogen bonds (No. Bonds) between the protein and drug, while the x-axis shows the “frames” or “snapshots” during which these hydrogen bonds were measured.
2. What is the maximum number of hydrogen bonds formed between the mutant Abl kinase protein and imatinib?________

**Comparison of Hydrogen Bonds**

1. Let’s now compare the wild type and mutant protein’s hydrogen bond interactions with the drug.
   1. Does the maximum number of hydrogen bonds present in the wild type differ from the mutant? If so, how do they differ?

|  |
| --- |

- 1. You should have observed that the maximum number of hydrogen bonds in the mutant is lower than that of the wild type protein. This difference is due to the mutation we learned about earlier. When the protein kinase has a threonine in position 315, like in the wild type protein, it can form a hydrogen bond. However, when it gets mutated to an isoleucine, that hydrogen bond is lost because isoleucine doesn’t have an oxygen, nitrogen or fluorine atom to participate in hydrogen bonding.


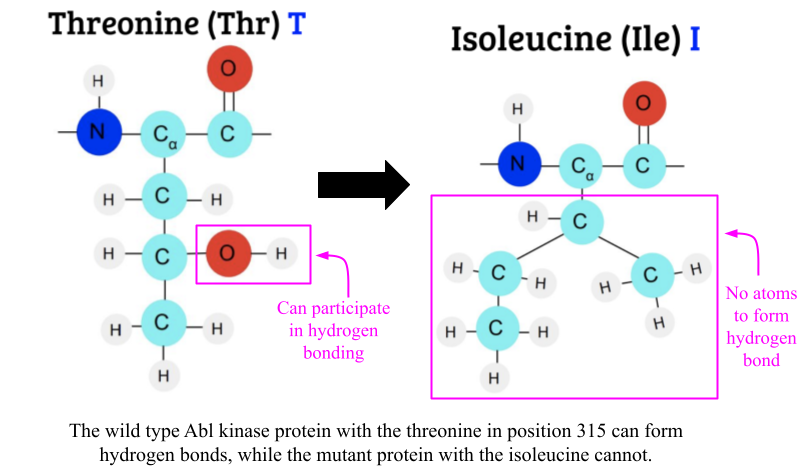


- 1. What other observations do you make between the wild type and mutant proteins?

|  |
| --- |

- 1. What other data analysis do you think we can do to better understand the difference in hydrogen bonding between the wild type and mutant proteins?

|  |
| --- |

1. One way we can compare the difference in hydrogen bonding between the wild type and mutant proteins is through statistical analysis. Using the Hydrogen Bond analysis tools provided by VMD, we can extract a spreadsheet with all the numbers of hydrogen bonds between the protein and drug in both the simulations as a function of time. What this means is that every 50 picoseconds, which is $1\times{10}^{-12}$ seconds, the software gives us the number of hydrogen bonds that exist. The spreadsheet looks like this.


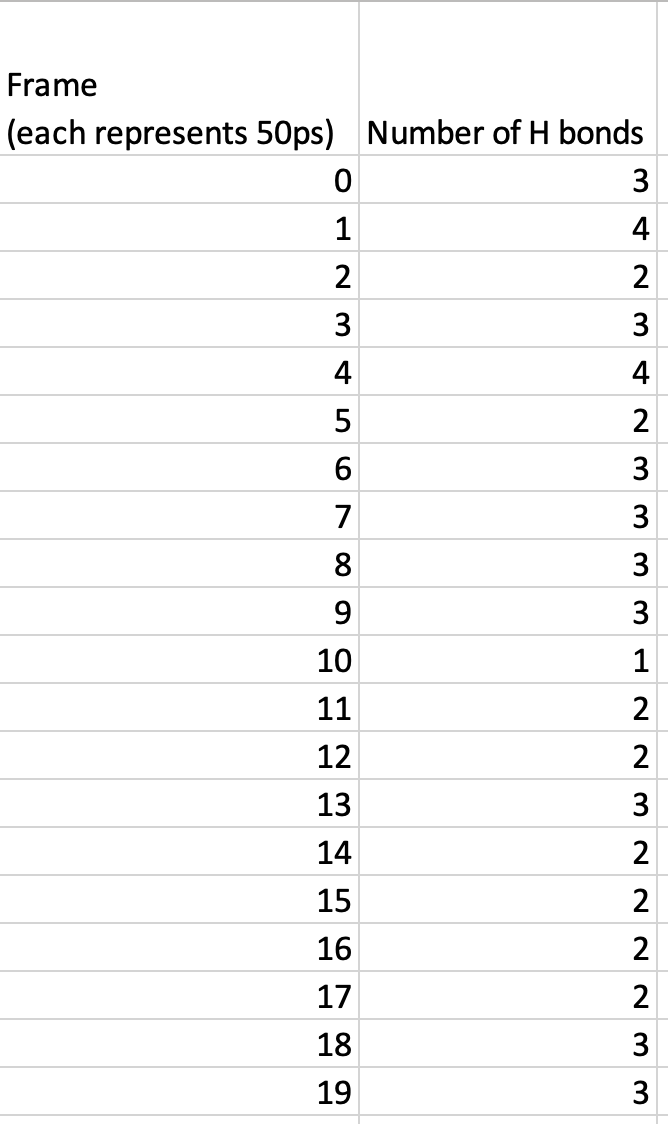


1. As you can see, the number of hydrogen bonds is changing over time. Why do you think this would be helpful for researchers to study? How is using an MD simulation to do this analysis advantages over other methods such as looking at the static structure?

|  |
| --- |

1. We can do some statistical analysis on the data and find the average number of hydrogen bonds between the protein and drug over the entire simulation in both the wild type and mutant proteins. Doing so we get the following data.

|  | **Wild type protein + imatinib** | **Mutant protein + imatinib** |
| --- | --- | --- |
| Average number of hydrogen bonds between the protein and the drug | 2.7 | 2.5 |
| Standard deviation | 0.8 | 0.6 |

1. This data shows that for the wild type protein and imatinib interactions, there are $2.7\pm0.8$ hydrogen bonds taking place over the entire simulation. For the mutant, there is on average $2.5\pm0.6$ hydrogen bonds taking place between the protein and drug over the entire simulation.
2. We can do some further analysis on the data to determine whether this difference we see in the average number of hydrogen bonds is statistically significant. Doing this analysis yields a p-value of ${8.4\times10}^{-8}$. For our results to be statistically significant, our p-value should be under 0.05. Our value of ${8.4\times10}^{-8}$ is much smaller than 0.05 so our results are in fact statistically significant.
3. This means that the number of hydrogen bonds we observed between the wild type protein and drug as well as the mutant protein and drug are likely not caused by chance!

**Distance and Fluctuation Analysis**

We learned that by studying MD simulations, we can learn more about the dynamic nature of molecules. Since molecules are moving around throughout the duration of our simulations, the distances between the different amino acids in the protein itself as well as between the protein and drug can change. In this activity, we will be investigating how distances change over the course of the simulation.

Since we just looked at the hydrogen bonds of the mutant protein kinase, let’s start by looking at some of the distances between the **mutant** protein and drug.

1. In the VMD Main window click “Graphics” → “Representations”. A “Graphical Representations” window should appear.
2. To distinguish between the protein and drug, we will place in two different Drawing Methods.
3. Let’s first focus on the drug. In the “Selected Atoms” field type “chain B” and press enter. Your drug should now be visible in your OpenGL Display window. If it is not, you can always recenter the molecule by clicking on the OpenGL Display window and clicking the “=” sign on your keyboard.
4. Now we want to change its drawing method to CPK. In the “Graphical Representations” window, under “Drawing Methods”, click on CPK. Now we can see the individual atoms making up the imatinib drug.


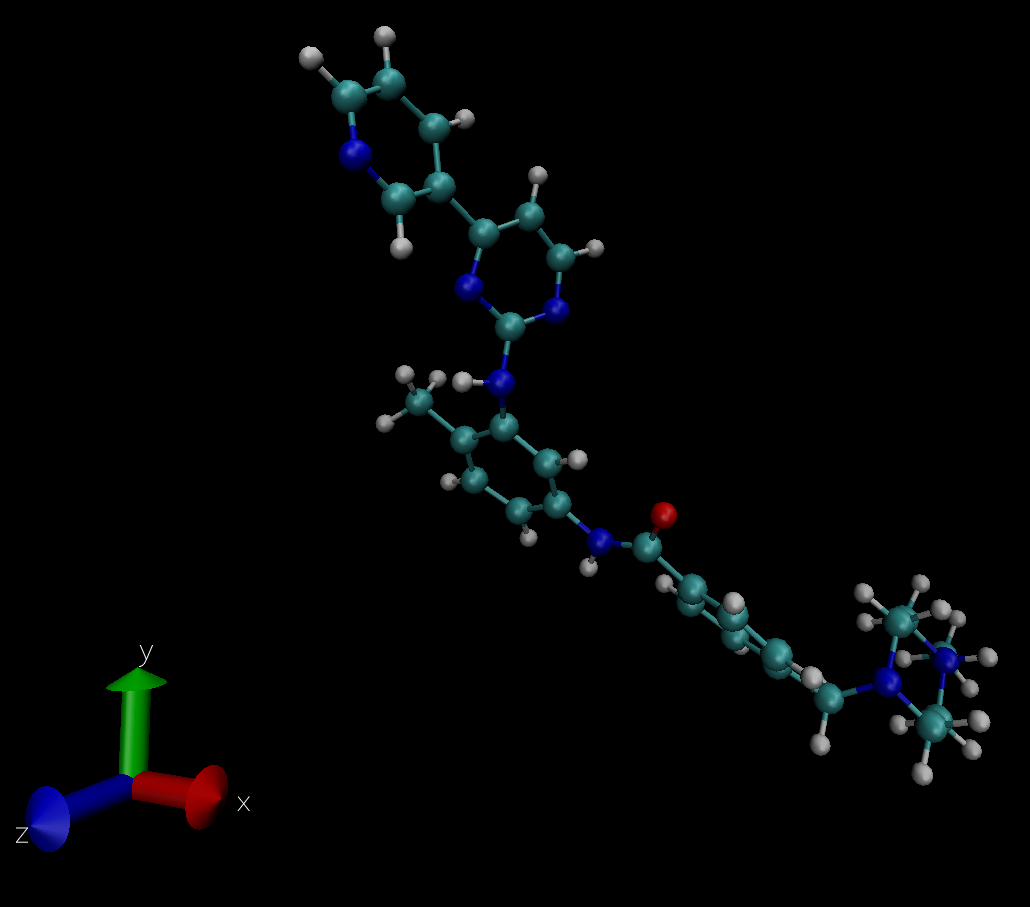


1. Let’s now investigate a hydrogen bond. One hydrogen bond that exists between protein and drug is between the oxygen atom on glutamic acid 286’s side chain and the hydrogen atom attached to the nitrogen in the bent portion of the molecule.
2. Let’s label this hydrogen bond. To do so, in the “Graphical Representation” window create a new representation by pressing “Create Rep”, and type “same residue as within 3.5 of chain B”. This will bring up all the residues that are within 3.5 angstroms of the drug (chain B). You can change the Drawing Method to Lines to help visualize the molecules better. Your OpenGL Display window should look like this:


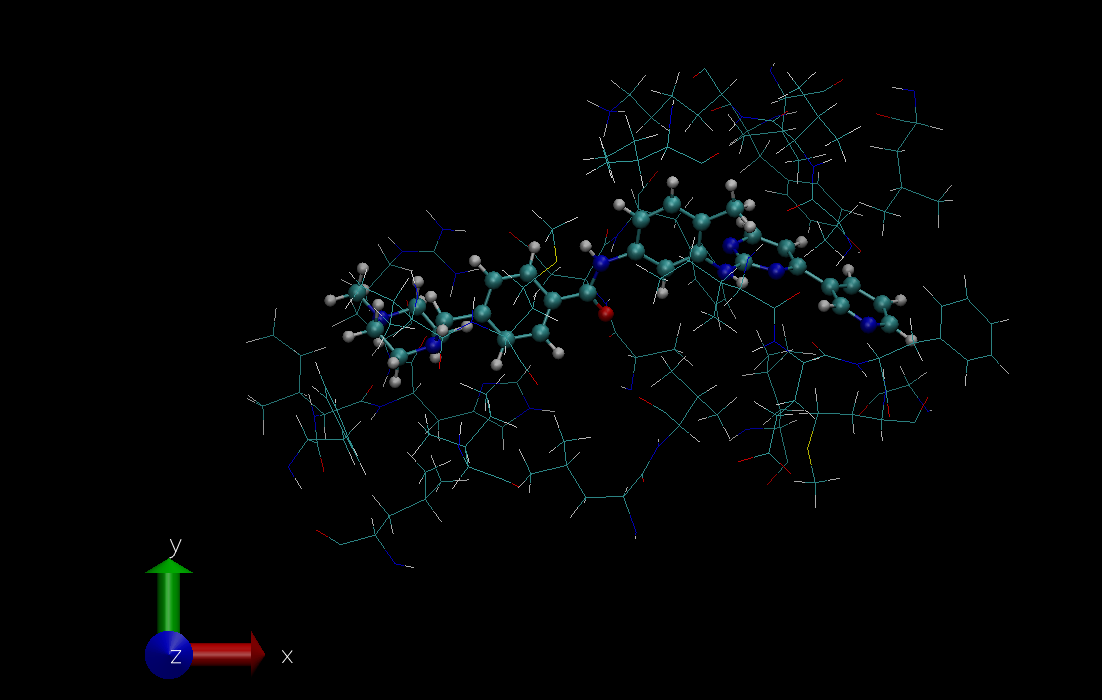


1. We now want to find the distance between the drug and this glutamic acid. To do so, find the nitrogen atom on the drug and the residues near it.
2. Press “2” and press on the hydrogen atom attached to the nitrogen on the drug and then press the oxygen of the glutamic acid molecule. You should get something that looks like this:


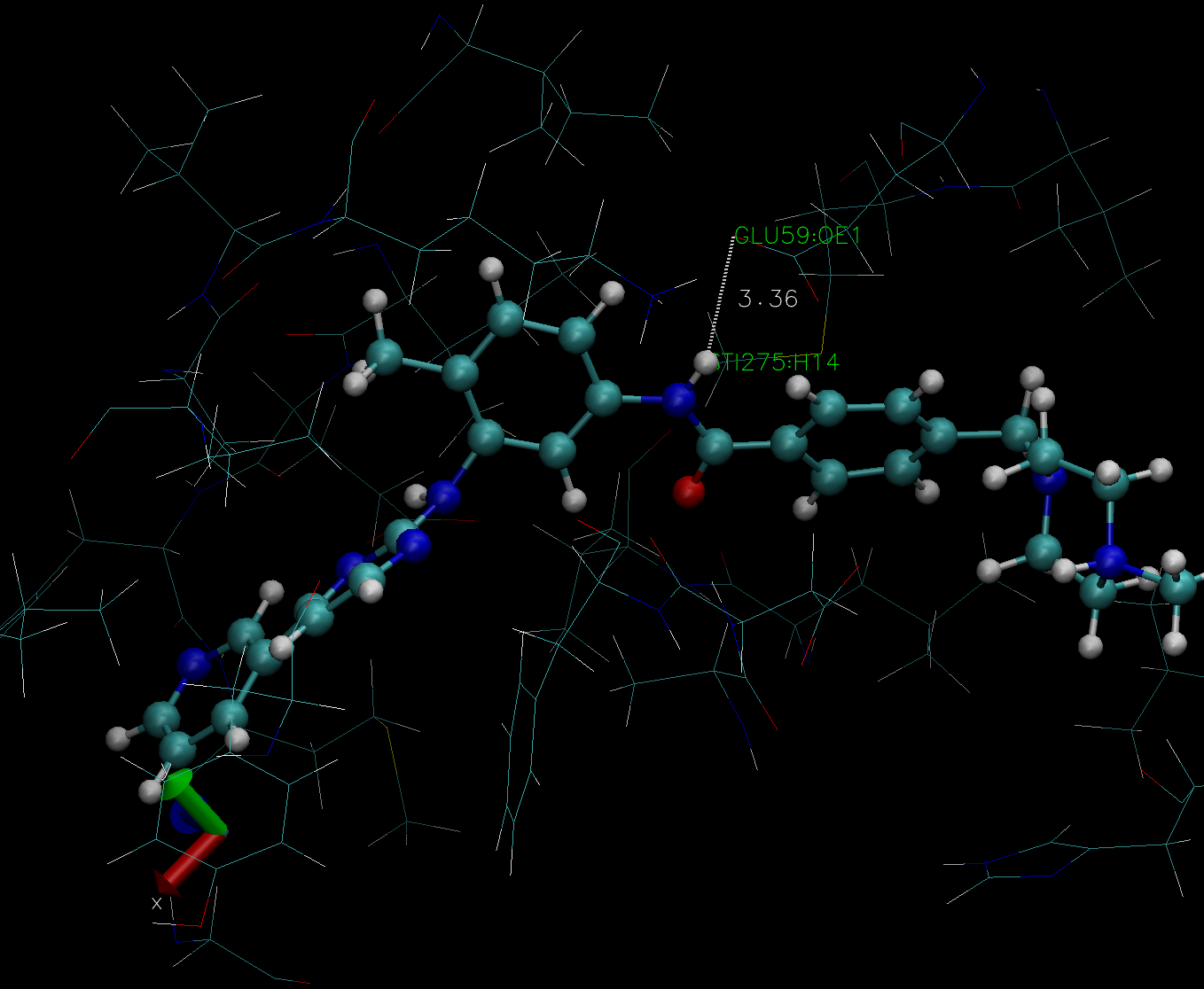


1. Let’s now observe what happens when we run the simulation. What do you predict will happen to the distance we just labeled between the protein and drug?

|  |
| --- |

1. To more easily observe what is going to happen, we are going to open a new window that displays the distances as they change over time.
2. In the VMD Main window press “Graphics” → “Labels”. A window will appear.
3. In this new “Labels” window, change “Atoms” to “Bonds” and then press on the bond you see there so it is highlighted in green. Your window should look like this:


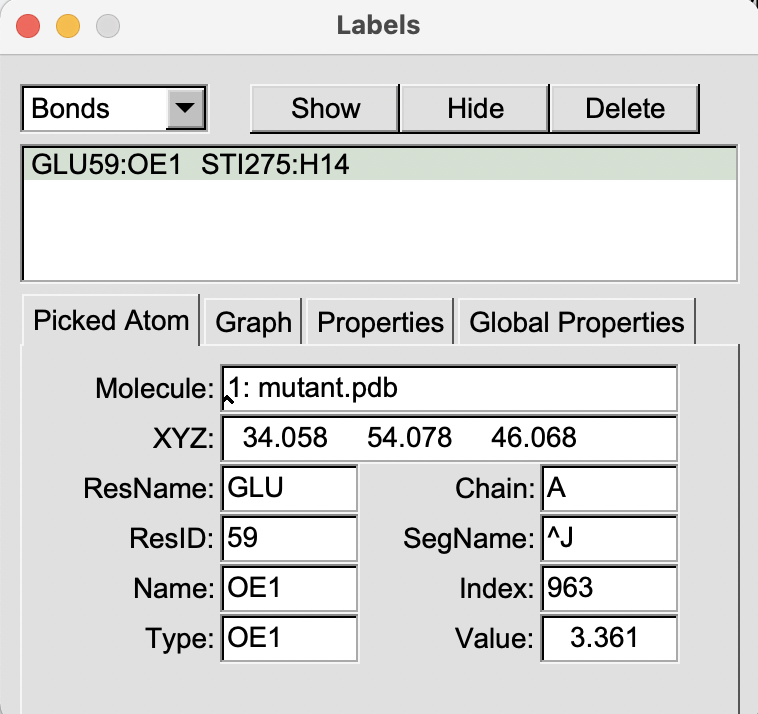


1. In the bottom right corner there is a field that says “Value”. This represents the distance we just identified between our protein and drug.


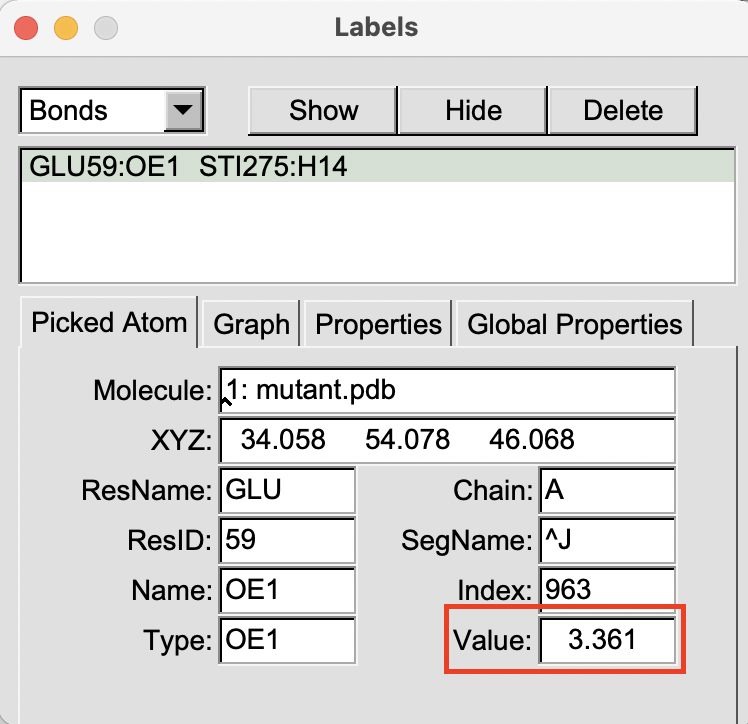


1. At this moment in time, the speed of our simulation is very fast so we are going to want to slow it down. In the “VMD Main” window slide the slider for speed all the way to the left.


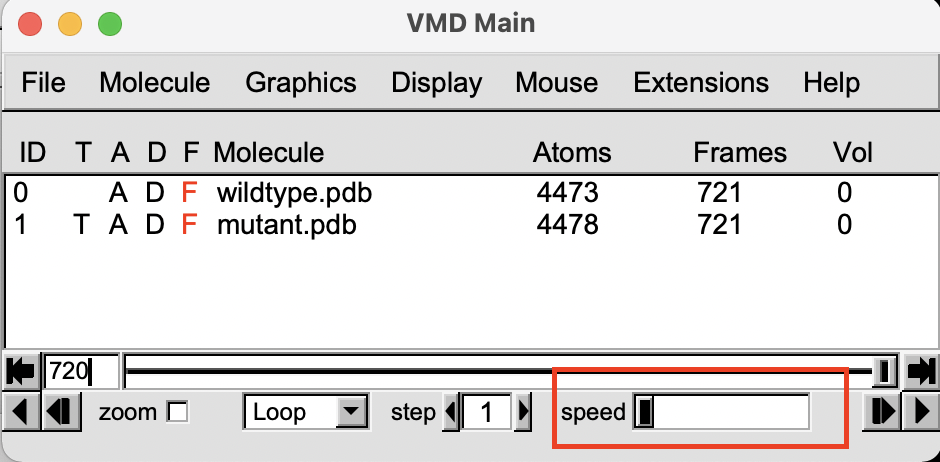


1. Now press the play button in the “VMD Main” window. Your simulation will start playing.


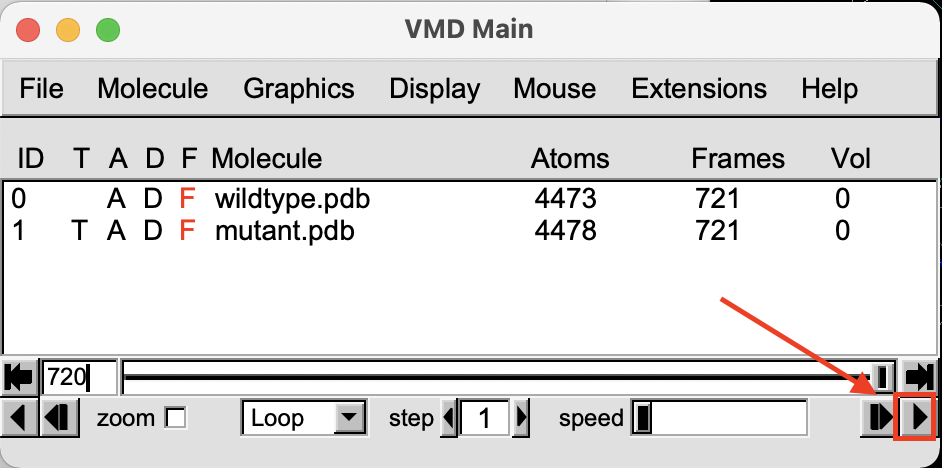


1. Return to the “Labels” window and observe the “Value” number. What do you see? Write down your observations.


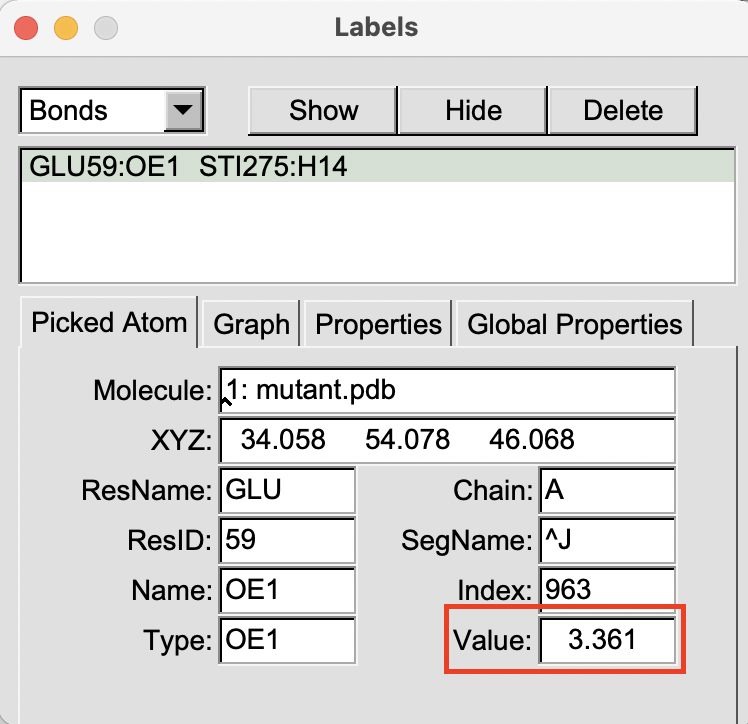


|  |
| --- |

1. We can also get a graph of what is happening to this bond over time. To do so, press on the “Graph” tab in the “Labels” window.


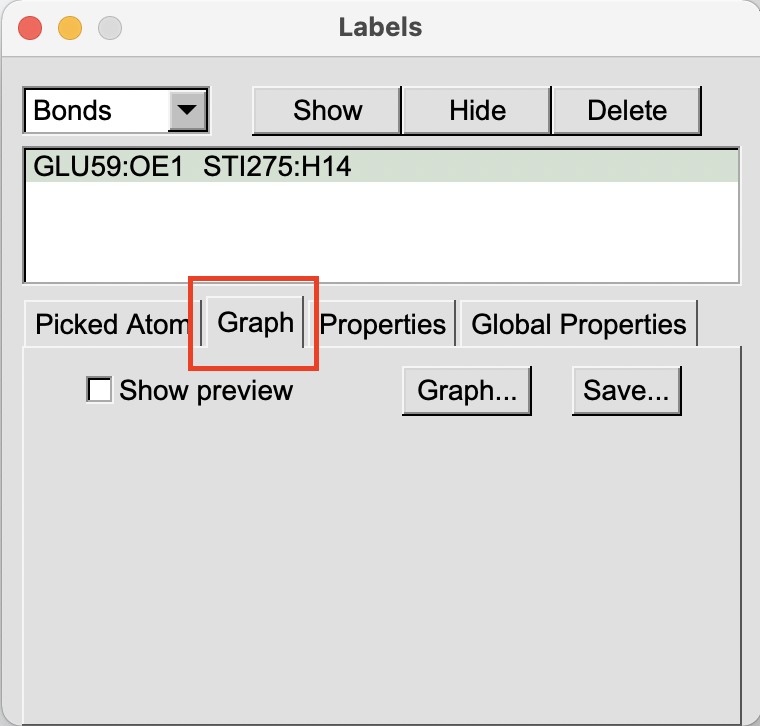


1. Press “Graph…”. A graph of the change in distance of the bond length over time will appear. Take a screenshot and paste it below.

|  |
| --- |

1. What observations can you make?

|  |
| --- |

1. Return to the “VMD Main” window and press the play button again so that the simulation stops playing.


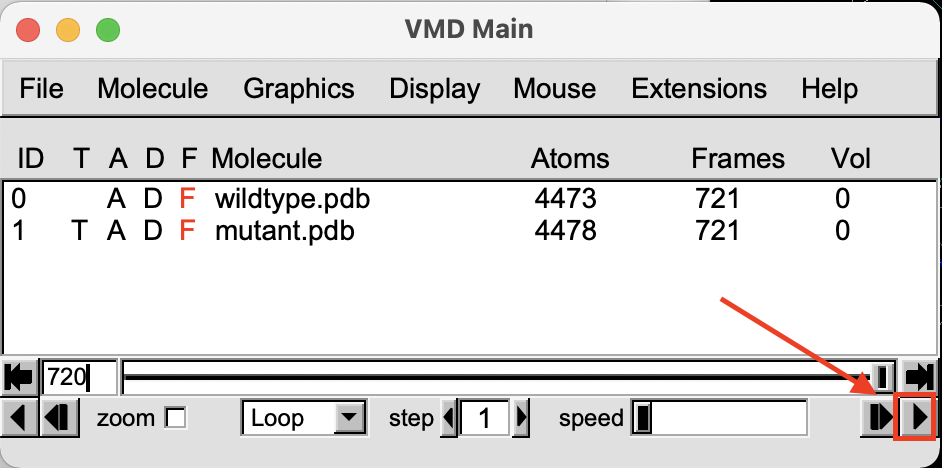


1. We can do a similar analysis of the wild type protein.
2. Close the graph you just generated.
3. In the “Labels” window we are going to delete all our labels. Start by deleting the bonds.


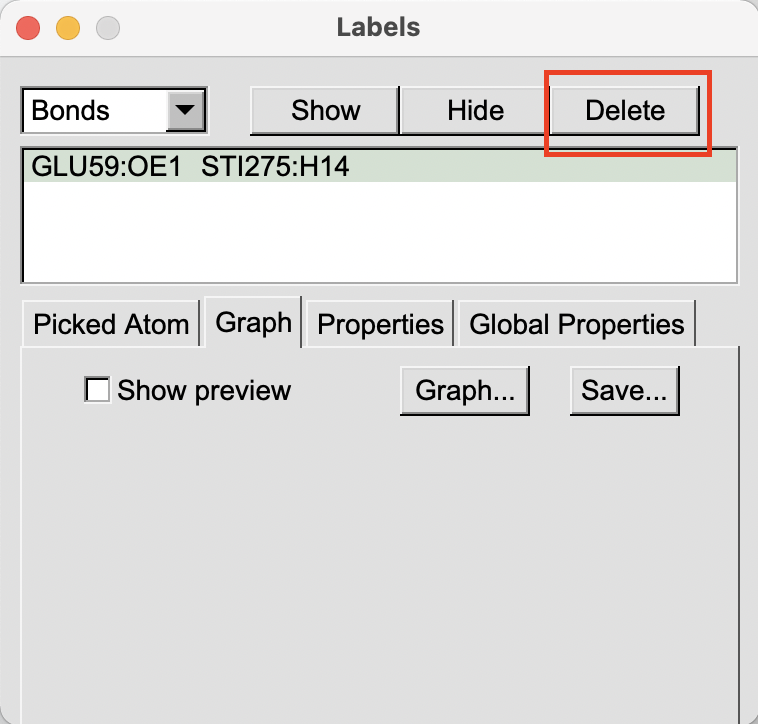


1. And then delete the atoms. In order to delete the atoms make sure they are highlighted in green.


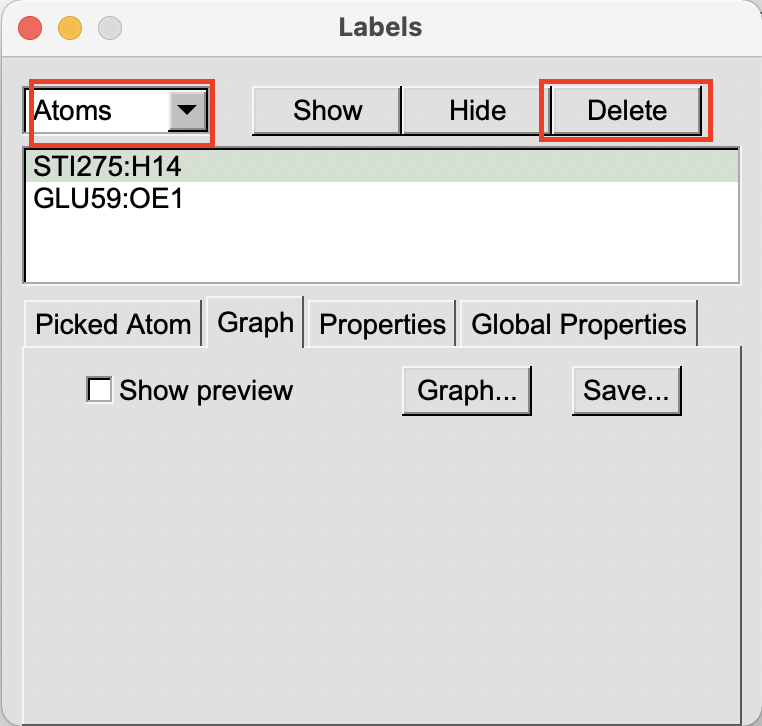


1. Now in the “Graphical Representations” window, delete all your representations.


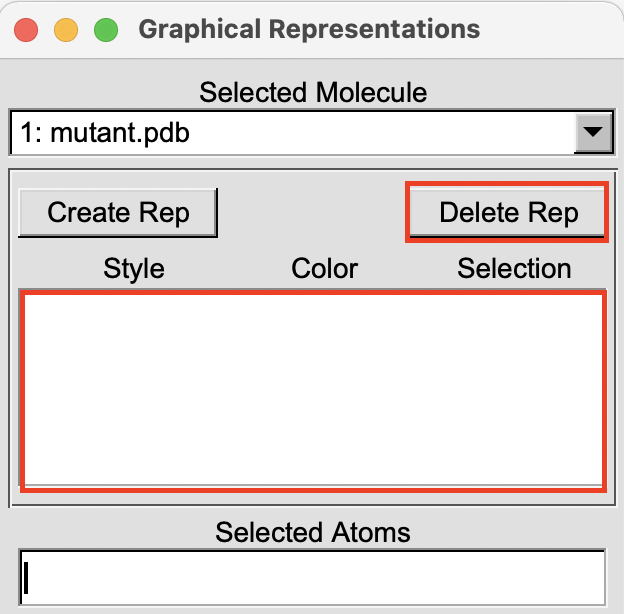


1. Now, in the “Graphical Representations” window, switch to wildtype.pdb.


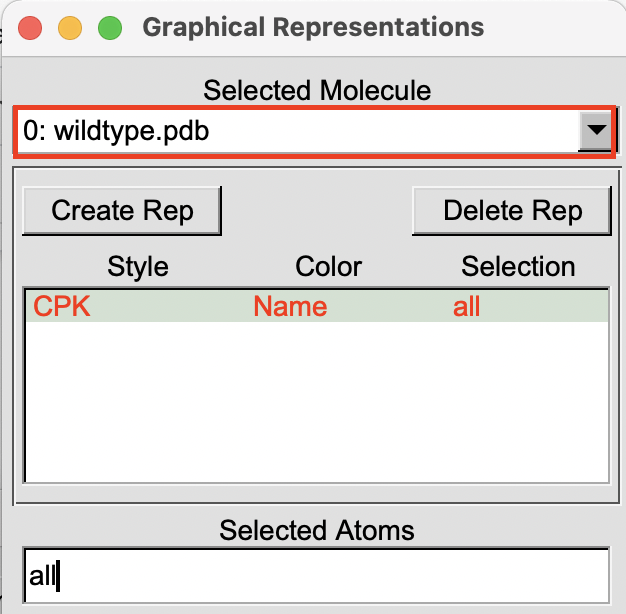


1. Double click on the red “all” and your wildtype Abl kinase and imatinib will appear in the “OpenGL Display” window. You can press “=” on your keyboard to recenter it.


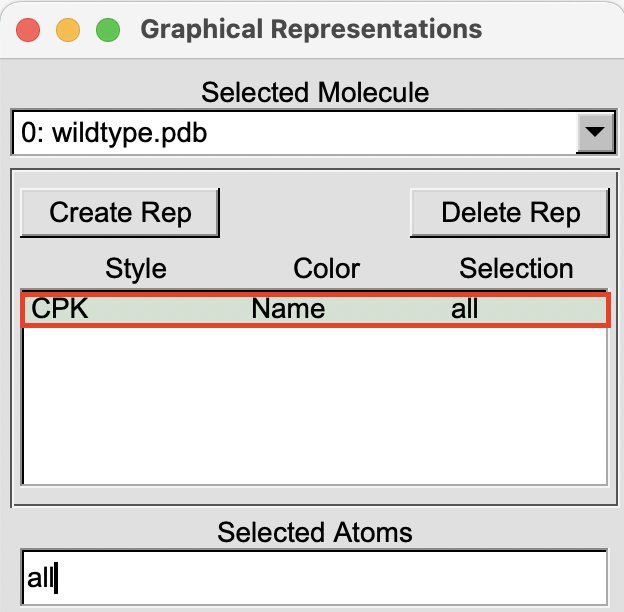


1. We are now going to similarly find the hydrogen bond between Glutamic acid 59 and the drug. To do so, we are going to type in the same commands as before. Make sure to create 2 different representations. Your “Graphical Representations” window should look like this. “Chain B” should be in CPK and “same residue as within 3.5 of chain B” should be in Lines.


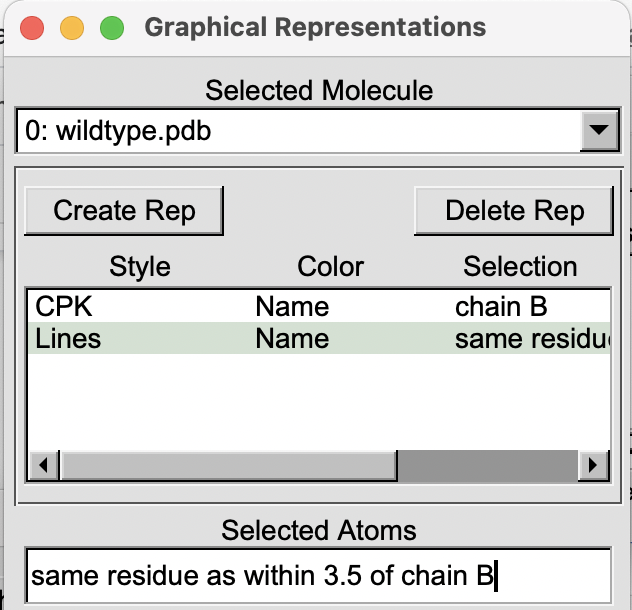

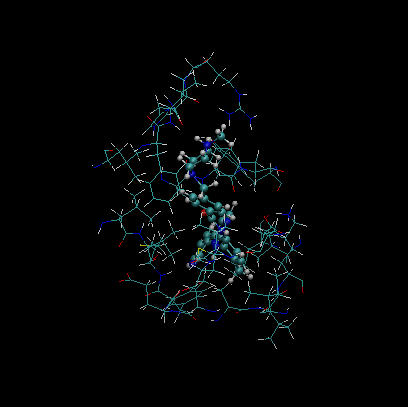


1. Now we are going to find the Glutamic acid residue and draw a bond between the oxygen on its side chain and the hydrogen attached to the nitrogen on the drug.


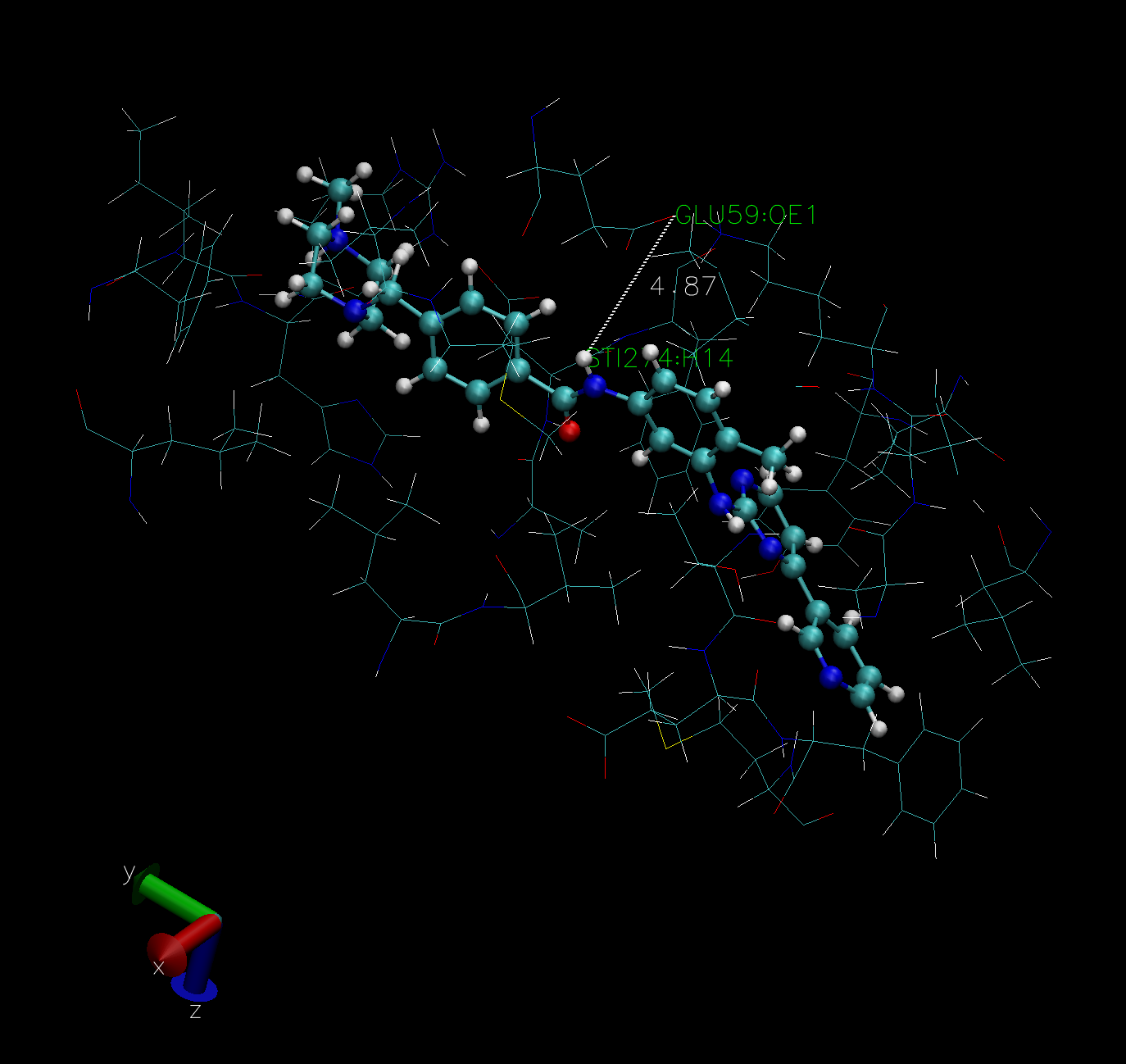


1. Next, return to the “Labels” window. If you closed the window, you can reopen it by going to the “VMD Main” window and pressing “Graphics” → “Labels”.
2. Go to “Bonds” and “Picked Atoms” and click on the bond so it’s highlighted in green.
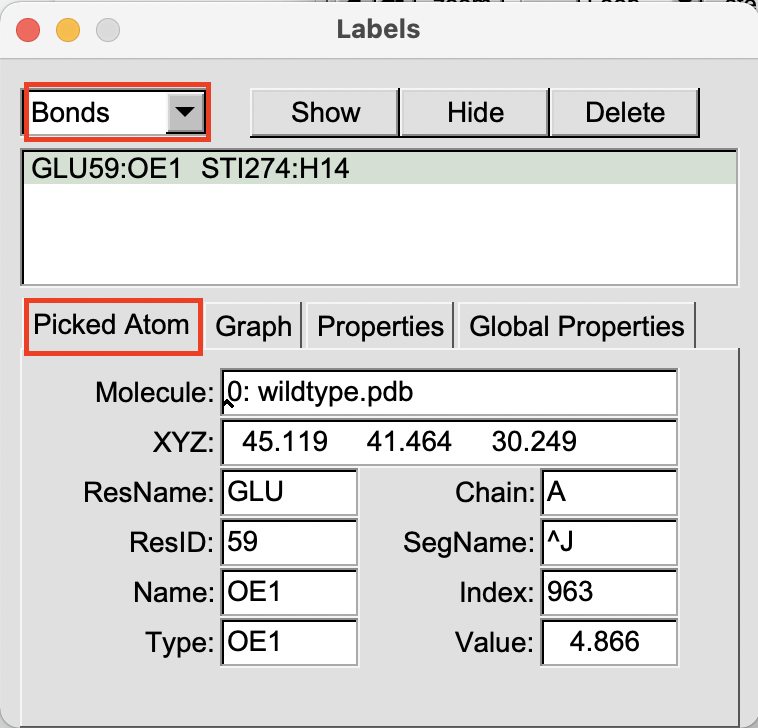

3. Return to the “VMD Main” window and click the play button.


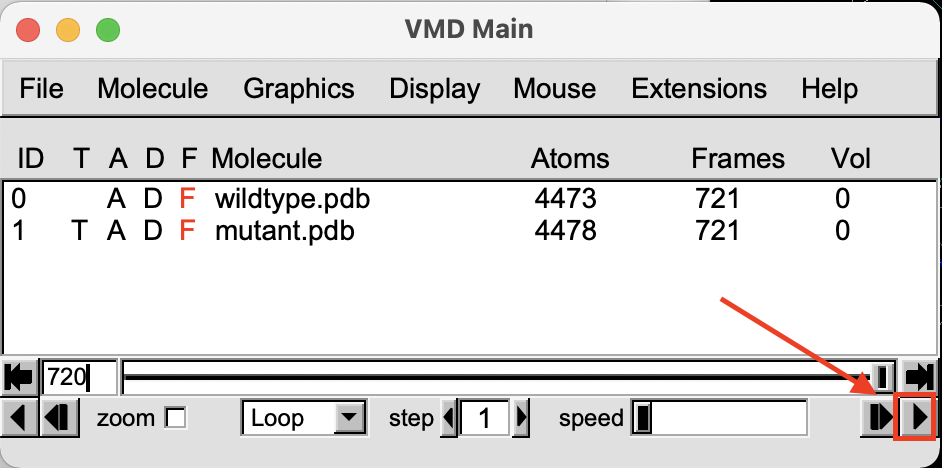


1. Return to the “Labels” window and observe the “Value” number. What do you see? Write down your observations.


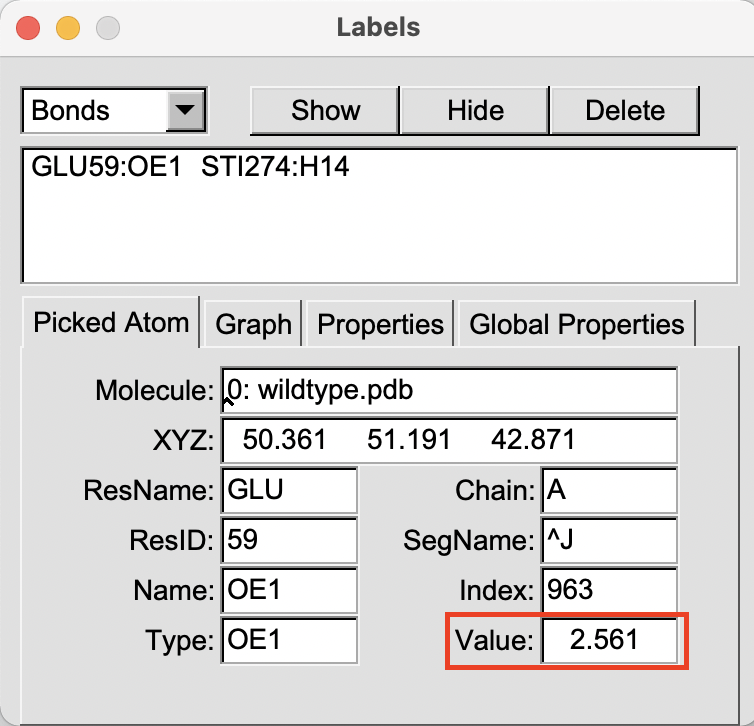


|  |
| --- |

1. Similarly to the mutant protein, we can generate a graph of how the distance this hydrogen bond changes over time. In the “Labels” window, make sure your bond is highlighted in green.


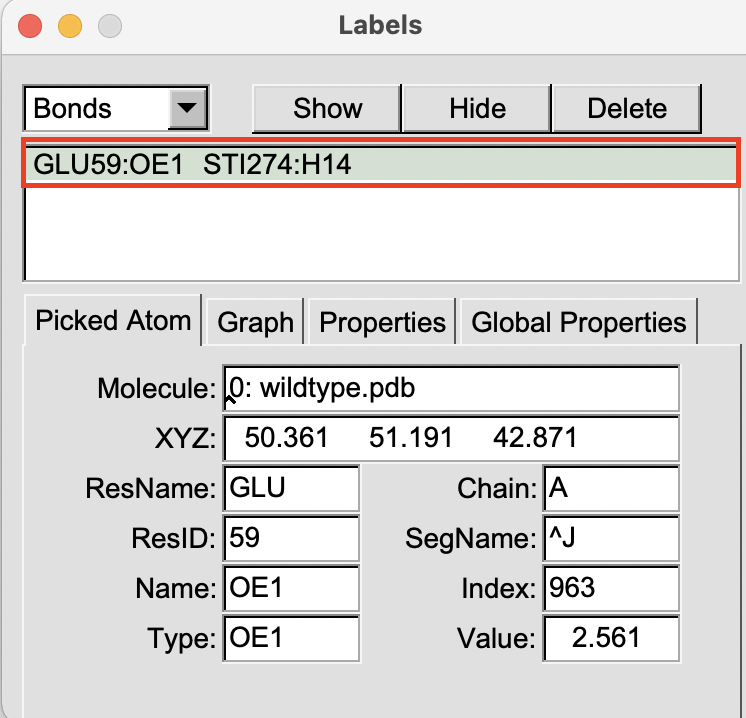


1. Go to the “Graph” tab and press “Graph…”. A graph of the change in distance of the bond length over time will appear. Take a screenshot and paste it below.

|  |
| --- |

1. What observations can you make?

|  |
| --- |

1. Comparing the wild type and mutant graphs, what similarities and differences do you see?

|  |
| --- |

As you have observed, the length of the hydrogen bond between the protein and drug changes over time. One important type of data analysis researchers conduct is called **root mean square fluctuation (RMSF) analysis**. ​​RMSF analysis shows researchers how much an atom or a group of atoms moves around, in other words fluctuates, over the course of the simulation. This in turn gives a measure of how stable an atom or group of atoms is during a given period of time. More fluctuations means less stability because the molecule is moving around more.

Let’s look at the RMSF graphs for the imatinib drug in the MD simulations we studied today.

The x-axis of our graph is the atom number while the y-axis is the distance the atoms traveled measured in nanometers.


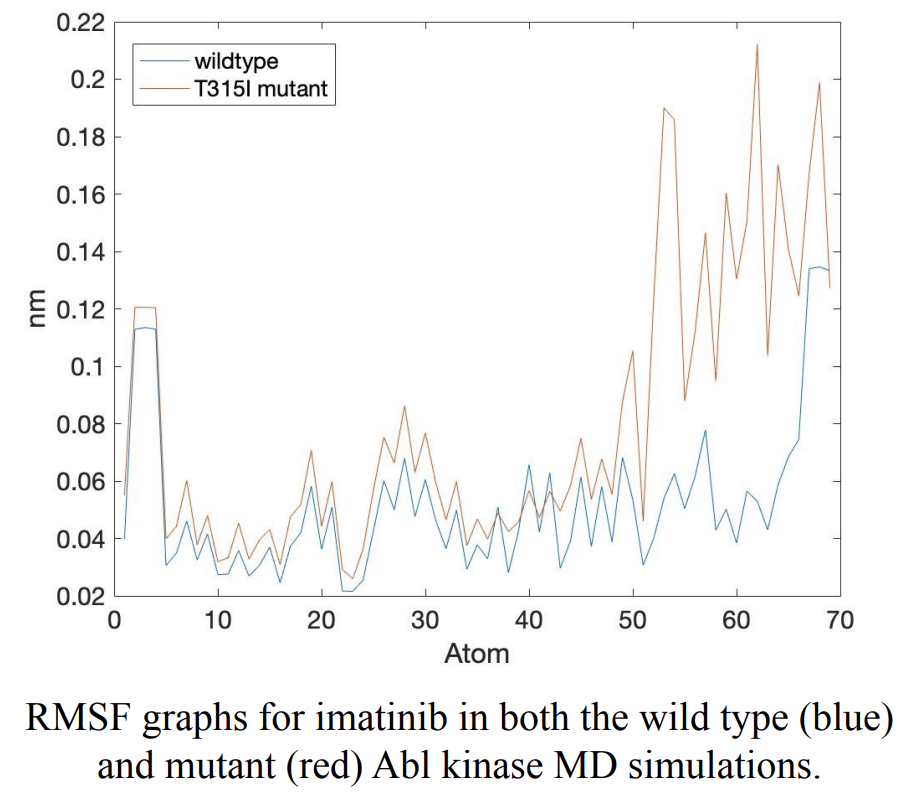


1. What similarities do you see between the two graphs? ____________________________
2. What differences do you see? _______________________________________________
3. Which one is more stable? __________________________________________________

In VMD we can map these RMSF values onto the molecule and view which parts of the molecules fluctuate the most.


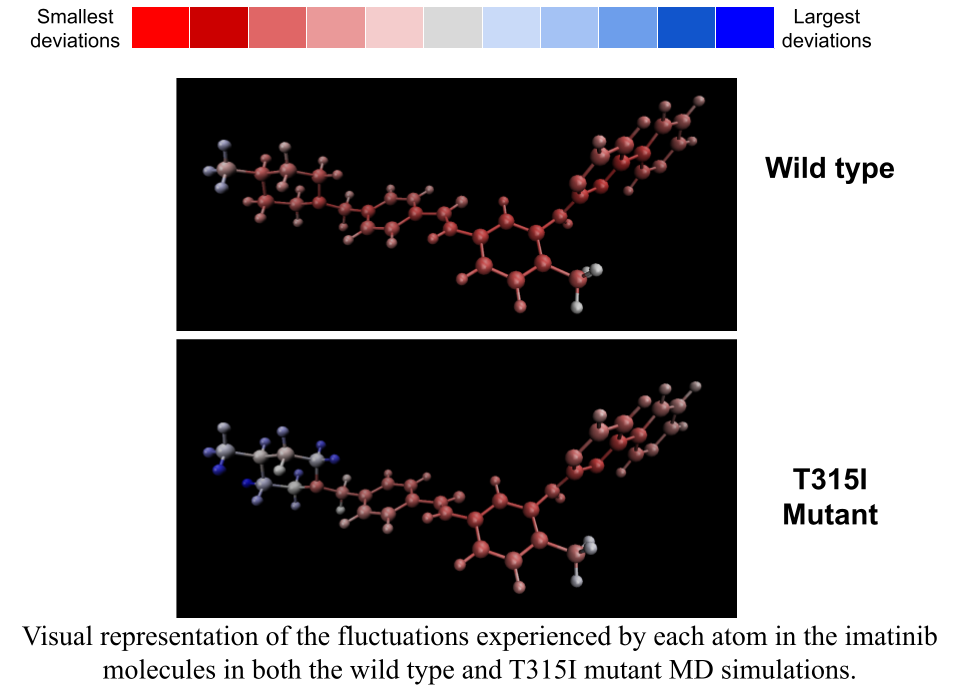


What do you notice about the different parts of the molecule when you compare the imatinib molecule in the wild type protein MD simulation compared to the imatinib molecule in the simulation with the T315I mutant protein?

|  |
| --- |

Now let’s look at the RMSF graphs for the Abl kinase protein in the MD simulations we studied.


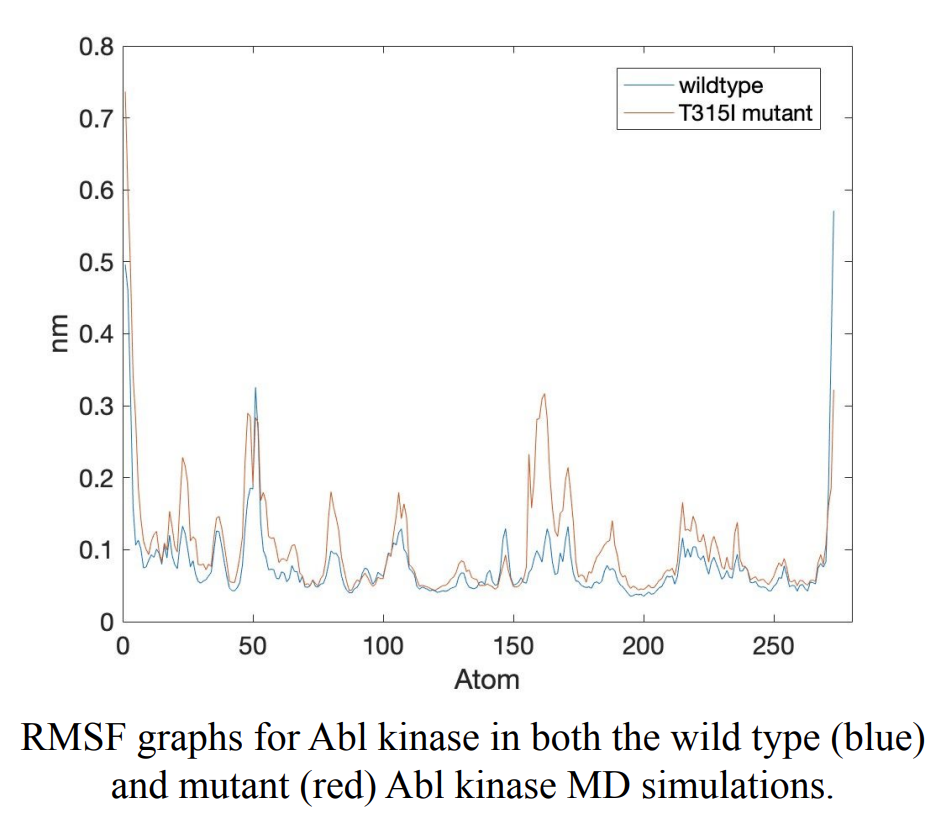


1. What similarities do you see between the two graphs? ____________________________
2. What differences do you see? _______________________________________________
3. Which one is more stable? __________________________________________________

Similar to above, we can also visualize these fluctuations in VMD.


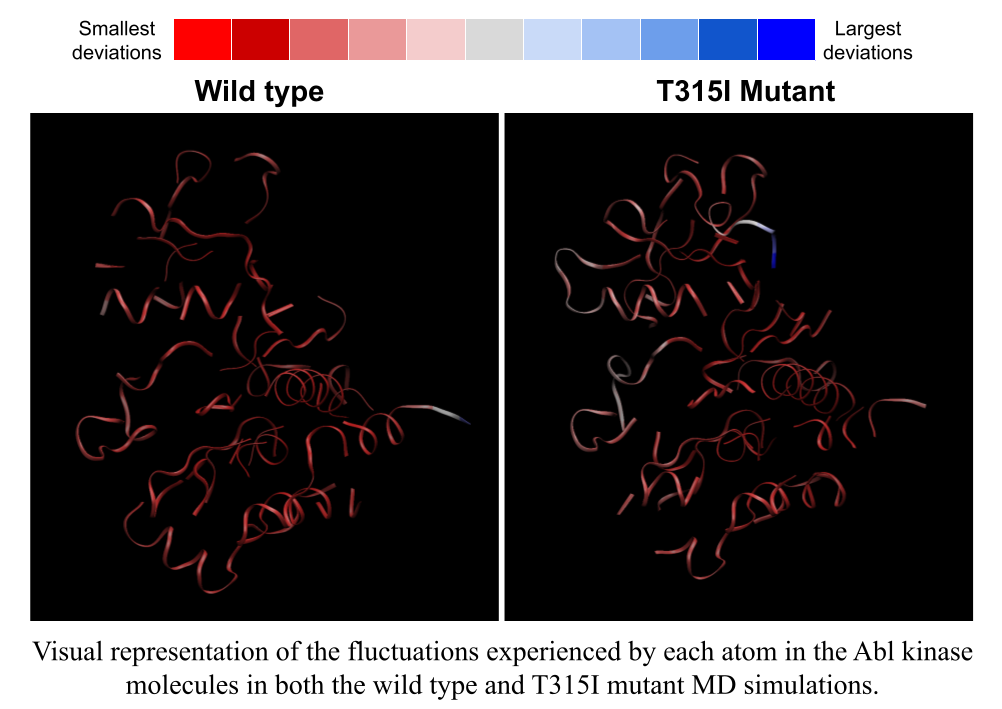


Here the protein in its secondary structure is shown. What observations can you make regarding similarities and differences between the wild type and T315I mutant proteins?

|  |
| --- |

**Discovery of Ponatinib**

With the advent of this T315I mutant, researchers began developing second and third generation tyrosine kinase inhibitors. The second generation TKIs were unable to overcome this T315I mutation but ponatinib, a third generation drug was! Ponatinib was the drug we worked with in the first few activities. Its structure is shown below. What is special about ponatinib is that it has a triple bond that “passes over” the T315I mutation, so whether the protein acquires this mutation or not, it doesn’t matter, because the drug is not relying on a hydrogen bond interaction with the threonine residue at position 315.

The other important group on this drug is the trifluoride group. This group is important because it induces strong Van der Waals interactions between the protein and drug. By maintaining multiple interactions with surrounding residues in the binding pocket, the effect that one mutation has on binding affinity decreases. So the drug is not relying on that mutating threonine to bind to the protein binding pocket.


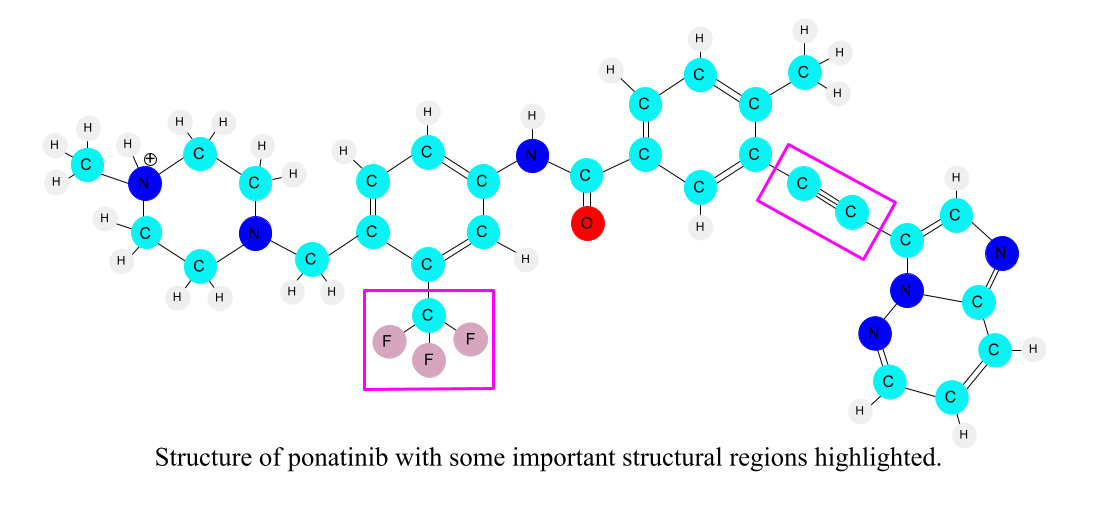


1. “What Is Chronic Myeloid Leukemia?: Leukemia Types.” *American Cancer Society*, 19 June 2018, https://www.cancer.org/cancer/chronic-myeloid-leukemia/about/what-is-cml.html. [↑](#footnote-ref-0)
2. Sriram, K., and Insel, P. A. (2018) G Protein-Coupled Receptors as Targets for Approved Drugs: How Many Targets and How Many Drugs? *Mol. Pharmacol. 93*, 251–258. [↑](#footnote-ref-1)
3. Tusa, I., Cheloni, G., Poteti, M., Silvano, A., Tubita, A., Lombardi, Z., Gozzini, A., Caporale, R., Scappini, B., Dello Sbarba, P., and Rovida, E. (2020) In Vitro Comparison of the Effects of Imatinib and Ponatinib on Chronic Myeloid Leukemia Progenitor/Stem Cell Features. Target. Oncol. 15, 659–671. [↑](#footnote-ref-2)
4. Druker, B. J., Gathmann, I., Kantarjian, H., Deininger, M. W. N., Goldman, J. M., Hochhaus, A., Rousselot, P., Hughes, T., Verhoef, G., Gratwohl, A., Simonsson, B., So, C., and Larson, R. A. (2006) Five-Year Follow-up of Patients Receiving Imatinib for Chronic Myeloid Leukemia. N Engl J Med 10. [↑](#footnote-ref-3)
5. Druker, B. J.; Sawyers, C. L.; Kantarjian, H.; Resta, D. J.; Reese, S. F.; Ford, J. M.; Capdeville, R.; Talpaz, M., Activity of a specific inhibitor of the BCR-ABL tyrosine kinase in the blast crisis of chronic myeloid leukemia and acute lymphoblastic leukemia with the Philadelphia chromosome. N. Engl. J. Med. 2001, 344 (14), 1038-1042. [↑](#footnote-ref-4)
6. Gorre, M. E.; Mohammed, M.; Ellwood, K.; Hsu, N.; Paquette, R.; Rao, P. N.; Sawyers, C. L., Clinical resistance to STI-571 cancer therapy caused by BCR-ABL gene mutation or amplification. Science 2001, 293 (5531), 876-880. [↑](#footnote-ref-5)
