# Supplementary material for: EMMAs: Implementation and Assessment of a Suite of Cross-Disciplinary, Case-Based High School Activities to Explore Three-Dimensional Molecular Structure, Noncovalent Interactions, and Molecular Dynamics
Source: J Chem Educ. 2024 May 10;101(6):2436–47. doi: 10.1021/acs.jchemed.4c00036 (PMC11171454; doi:10.1021/acs.jchemed.4c00036)
Supplement: Supplementary file 1 — ed4c00036_si_001.zip [file ed4c00036_si_001.zip › Kotsalidis_supporting_info_revisions/00 - Instructions for downloading and installing Visual Molecular Dynamics Software.docx]

**Instructions for downloading and installing Visual Molecular Dynamics Software**

**a)** Visit the [VMD download and installation website](https://www.ks.uiuc.edu/Development/Download/download.cgi?PackageName=VMD)

**b)** Choose the installation that matches your computer's operating system. For Mac users: you can find your operating system by clicking on the apple in the upper left hand corner of your Mac Desktop and clicking on "About this Mac". If your Mac is less than 2 years old, you probably should choose the VMD 1.9.4 option. Otherwise, choose the VMD 1.9.3 version. If you have a PC, you should probably choose the Windows OpenGL version under 1.9.3 (not the CUDA one).

**c)** Before the download proceeds, you will need to "register" -- simply enter in a username and a password of your choice to register and follow the instructions for the download.

**d)** Once the installer has been downloaded, click on it to do the install. Note that if you have a Mac, you will need to press "control" before you click on the installer to bypass the "firewall" that protects you from installing something that isn't an apple-verified product (but fear not, as this is a widely-used piece of software) [If control-clocking doesn't work, you might need to open up security settings and enable it to open from there -- this sometimes involves clicking on a button to open the application]

**e)** The product should install at this point, and it should create an icon that looks like a water molecule to click on to launch it. NOTE: If you're on a MAC and this icon doesn't end up in the applications folder, you will need to click and drag it into the applications folder for it to launch properly. You might also need to give permission for the software to use other programs, such as terminal – Please give permission. (we have found that this sometimes just involves opening up System Preferences/Privacy and Security and literally just having that window open when you ctrl-click on the VMD icon in the applications folder).
