# Supplementary material for: EMMAs: Implementation and Assessment of a Suite of Cross-Disciplinary, Case-Based High School Activities to Explore Three-Dimensional Molecular Structure, Noncovalent Interactions, and Molecular Dynamics
Source: J Chem Educ. 2024 May 10;101(6):2436–47. doi: 10.1021/acs.jchemed.4c00036 (PMC11171454; doi:10.1021/acs.jchemed.4c00036)
Supplement: Supplementary file 1 — ed4c00036_si_001.zip [file ed4c00036_si_001.zip › Kotsalidis_supporting_info_revisions/D - EMMAs State_NGSS_AP College Board Items Addressed.docx]

**Exploring Molecular Modeling through case-based Activities (EMMAs):**

**State Standards, NGSS, and AP* Chemistry Course and Exam Description Items Addressed**

[**2016 Massachusetts Science and Technology Engineering Framework**](https://www.doe.mass.edu/frameworks/scitech/2016-04.pdf)

[**Next Generation Science Standards**](https://www.nextgenscience.org/search-standards?keys=&tid%5B%5D=107&tid_3%5B%5D=97)

**HS-PS1-3**

**PS2.B Types of interactions**

**HS-PS1-3**

**PS1.A: Structure and Properties of Matter**

**PS2.B: Types of Interactions**

**HS-PS2-6**

[**AP* Chemistry Course and Exam Description**](https://apcentral.collegeboard.org/media/pdf/ap-chemistry-course-and-exam-description.pdf)

**Learning Objective SAP-5.A**

**SAP-5.A.1**

**SAP-5.A.2**

**SAP-5.A.3**

**SAP-5.A.4**

**SAP-5.A.5**
