# Supplementary material for: EMMAs: Implementation and Assessment of a Suite of Cross-Disciplinary, Case-Based High School Activities to Explore Three-Dimensional Molecular Structure, Noncovalent Interactions, and Molecular Dynamics
Source: J Chem Educ. 2024 May 10;101(6):2436–47. doi: 10.1021/acs.jchemed.4c00036 (PMC11171454; doi:10.1021/acs.jchemed.4c00036)
Supplement: Supplementary file 1 — ed4c00036_si_001.zip [file ed4c00036_si_001.zip › Kotsalidis_supporting_info_revisions/05 - Secret Code Form.docx]

**WORD 1**

________ ________ ________ ________ ________ ________ ________ ________ ________

**Clue #1 Clue #2 Clue #3 Clue #4 Clue #5 Clue #6 Clue #7 Clue #8 Clue #9**

**WORD 2**

________ ________

**Clue #1 Clue #2**

**WORD 3**

________ ________ ________ ________ ________ ________ ________ ________ ________ ________

**Clue #1 Clue #2 Clue #3 Clue #4 Clue #5 Clue #6 Clue #7 Clue #8 Clue #9 Clue #10**

**UNSCRAMBLED**

**WORD 1**

________ ________ ________ ________ ________ ________ ________ ________ ________

**WORD 2**

________ ________

**WORD 3**

________ ________ ________ ________ ________ ________ ________ ________ ________ ________
